# Preferential Amplification of CD8 Effector-T Cells after Transcutaneous Application of an Inactivated Influenza Vaccine: A Randomized Phase I Trial

**DOI:** 10.1371/journal.pone.0010818

**Published:** 2010-05-26

**Authors:** Behazine Combadière, Annika Vogt, Brice Mahé, Dominique Costagliola, Sabrina Hadam, Olivia Bonduelle, Wolfram Sterry, Shlomo Staszewski, Hans Schaefer, Sylvie van der Werf, Christine Katlama, Brigitte Autran, Ulrike Blume-Peytavi

**Affiliations:** 1 Institut National de Santé et de Recherche Médicale, INSERM U945, Paris, France; 2 Université Pierre et Marie Curie (UPMC - Univ Paris 06), Paris, France; 3 Assistance Publique – Hôpitaux de Paris (AP/HP), Laboratory of Immunity and Infection, Paris, France; 4 Clinical Research Center for Hair and Skin Science, Department of Dermatology and Allergy, Charite - Universitätsmedizin Berlin, Berlin, Germany; 5 INSERM U720, Paris, France; 6 Hôpital Pitié Salpêtrière, Service des Maladies Infectieuses, Paris, France; 7 Pasteur Institute of Paris, Université Denis Diderot – Paris VII, Centre National de Recherche Scientifique URA1966, Laboratoire de génétique moléculaire des virus respiratoires, Paris, France; 8 HIV Treatment & Research Unit, Department of Internal Medicine II, Hospital of the Johann Wolfgang Goethe-University Frankfurt am Main, Frankfurt am Main, Germany; 9 Centre d'Investigation Clinique at INSERM U945, Paris, France; University of Sao Paulo, Brazil

## Abstract

**Background:**

Current conventional vaccination approaches do not induce potent CD8 T-cell responses for fighting mostly variable viral diseases such as influenza, avian influenza viruses or HIV. Following our recent study on vaccine penetration by targeting of vaccine to human hair follicular ducts surrounded by Langerhans cells, we tested in the first randomized Phase-Ia trial based on hair follicle penetration (namely transcutaneous route) the induction of virus-specific CD8 T cell responses.

**Methods and Findings:**

We chose the inactivated influenza vaccine – a conventional licensed tetanus/influenza (TETAGRIP®) vaccine – to compare the safety and immunogenicity of transcutaneous (TC) versus IM immunization in two randomized controlled, multi-center Phase I trials including 24 healthy-volunteers and 12 HIV-infected patients. Vaccination was performed by application of inactivated influenza vaccine according to a standard protocol allowing the opening of the hair duct for the TC route or needle-injection for the IM route. We demonstrated that the safety of the two routes was similar. We showed the superiority of TC application, but not the IM route, to induce a significant increase in influenza-specific CD8 cytokine-producing cells in healthy-volunteers and in HIV-infected patients. However, these routes did not differ significantly for the induction of influenza-specific CD4 responses, and neutralizing antibodies were induced only by the IM route. The CD8 cell response is thus the major immune response observed after TC vaccination.

**Conclusions:**

This Phase Ia clinical trial (Manon05) testing an anti-influenza vaccine demonstrated that vaccines designed for antibody induction by the IM route, generate vaccine-specific CD8 T cells when administered transcutaneously. These results underline the necessity of adapting vaccination strategies to control complex infectious diseases when CD8 cellular responses are crucial. Our work opens up a key area for the development of preventive and therapeutic vaccines for diseases in which CD8 cells play a crucial role.

**Trial Registration:**

Clinicaltrials.gov NCT00261001

## Introduction

Inducing CD8 T cell-mediated protective responses would be beneficial in eliminating infected cells and limiting virus or cancer dissemination. Classical preventive vaccines, however, except for live viral vectors and multiple DNA immunizations, are designed to generate neutralizing antibodies. The use of live attenuated vaccines known to induce strong CD8 T cell responses is limited by the risk of uncontrolled virus dissemination in immunocompromised individuals (e.g., with HIV or elderly) as well as by vector or pathogen-specific pre-existing immunity that limits the efficacy of vaccine administration or readministration [1]–[4]. The development of successful vaccines against HIV, malaria, tuberculosis, and cancers will require efficient, potent, and durable T cell responses [5]–[8]. In some cases involving high virus variability, the cross-reactivity of CD8 responses may be beneficial for recall responses [7], [9]. Although there is still no clear definition of the quality of effector T cells required for protection, it is commonly accepted that one of its fundamental characteristics is the magnitude and the nature of T cell responses [10]. Recently, benchmarks were determined for primary CD8+ T cell responses in humans induced by two of the most effective vaccines ever developed, those against yellow fever and smallpox [11]. The importance of these responses has been shown in many viral diseases and cancers, in both mouse and human models [12]–[15], and their persistence has been observed in the absence of circulating antigens [16]–[19]. The generation of such immune cells is thus of crucial interest in studying long-term immune responses to pathogens and in vaccine development.

Recent advances in understanding the central role of antigen-presenting cells (APCs) in the skin have prompted numerous studies of this organ as an immunization route [20]–[23]. It has been suggested that differential targeting of epidermal or dermal APCs might also produce differential immune responses [21], [23]. The main routes of immunization in humans – the muscle and the subcutaneous layer – are low in dendritic cells (DCs), and vaccines injected by these routes generally require adjuvant to augment DC recruitment and activation and to improve their immunogenicity [24]. Moreover, recent reports of the involvement of epithelial DCs in CD8 cell cross-priming suggests that vaccination via the cutaneous route may help to induce cellular immune responses [25]–[27]. Numerous concepts for vaccine delivery to the skin have thus been developed, but have not yet met expectations.

Hence, strong evidence indicates that targeting vaccine to the skin should effectively induce cellular immune responses [24], [28]. Glenn and collaborators elegantly demonstrated the efficacy of transcutaneous (TC) immunization in inducing humoral immune responses in humans [25], [29]–[34]. Frerichs et al [35] recently introduced a skin preparation system for improved TC vaccine delivery based on skin surface abrasion with silicone carbide particles, eliciting humoral responses. However, the induction of T cell immune responses, so well documented in murine models after TC immunization, remains to be shown in human.

We previously demonstrated that penetration of topically applied nanoparticles increased after application of cyanoacrylate skin surface stripping (CSSS) to human skin explants: the particles entered epidermal Langerhans cells (LCs), possibly via hair follicles [36]. We recently proposed that the cellular responses we observed to vaccine compounds were induced by the vaccine's penetration through hair follicular ducts, which are surrounded by APCs (LCs and DCs) [36], [37]. In contrast to the interfollicular epidermis, the hair follicle infundibulum must be considered highly permeable, and skin DCs, including epidermal LCs, all residing in and around the hair follicles, may thus be highly accessible to topically applied vaccines [38]. In a first pilot study by our groups, we targeted an inactivated influenza vaccine at the hair follicles, with a protocol that used only a single CSSS procedure before the vaccine application. We found that TC vaccination after one CSSS procedure was safe and, most interestingly, effective in inducing cellular immune responses [37].

Questions nonetheless remain about how best to shape the nature and quality of human immune responses to vaccines. In particular, we wondered whether the site of antigen delivery would affect the nature and the quality of the immune response and whether this method of targeting the skin's APCs would be capable of inducing CD8 T cell responses to a conventional inactivated influenza vaccine designed to induce vaccine-specific antibodies. We thus conducted two randomized controlled phase I clinical trials simultaneously in two groups of healthy individuals and a group of HIV-infected patients. Because this strategy can be of major help for HIV vaccination, we proposed to include HIV patients in parallel to healthy subjects, for safety and immunogenicity of this new method of vaccination. The transcutaneous vaccination protocol used is based on a single CSSS procedure that allows the opening of the hair follicular duct prior to application of a combined tetanus and influenza (TETAGRIP®) vaccine, compared with the conventional intramuscular (IM) immunization. The safety of the TC vaccine application was confirmed. More importantly, we demonstrated for the first time that TC application of an influenza vaccine induced a significant increase in influenza-specific CD8 responses compared with the IM route. The effects of this application on the intensity and quality of the influenza–specific effector T cells were studied in detail.

## Results

### Safety of TC compared with IM vaccination by inactivated influenza/tetanus vaccine in healthy volunteers and HIV-infected patients

In this randomized, investigator-blinded comparative Phase I study, we first evaluated the safety of TC compared to IM administration of a licensed non-adjuvanted tetanus+inactivated influenza vaccine (TETAGRIP®) in healthy volunteers (cohort I) and in HIV-infected patients (cohort II). All 24 healthy individuals completed the study protocol (12 in each group). The HIV-infected cohort was terminated early due to the onset of influenza season, and only 14 patients completed the study protocol: 6 in the TC group and 8 in the IM group. Table 1 summarizes the demographic and baseline characteristics of each group.

**Table 1:** Demographic and Baseline Characteristics.

Cohort I		TC vaccination			IM vaccination	
Healthy Volunteers	n	median [range]	Q1–Q3	n	median [range]	Q1–Q3
Age	12	31 [22–40]	25–37	12	28 [18–40]	24–32
BMI*	12	22 [21–25]	21–23	12	22 [21–24]	21–23
Phototype II	8			8		
phototype III	4			4		
Phototype IV						

Cohort I		TC vaccination			IM vaccination	
HIV+ volunteers	n	median [range]	Q1–Q3	n	median [range]	Q1–Q3
Age	6	31[31–44]	33–44	8	40 [32–45]	37–45
BMI*	6	22 [21–26]	21–25	8	24 [21–25]	21–25
CD4 T cells						
Nadir (cells/ml)	6	308 [265–436]	274–365	8	266 [203–440]	226–347
baseline (cells/ml)	6	1022 [623–1257]	623–1257	8	724 [544–1082]	577–834
Baseline (%)	6	42 [35–51]	35–51	8	31 [20–40]	27–40
Vial Load (copies/ml)	6	<400		8	<400	
Phototype II	2			6		
phototype III	4			2		
Phototype IV						

*BMI Body mass Index.

The primary safety endpoint was clinical local and systemic tolerance to vaccine administration by each route. Safety analyses of all 24 healthy volunteers and all 14 chronically HIV-infected patients who completed the study showed that the TC mode of administration was well tolerated in both populations (Table 2). All reactions and adverse events during the follow-up period (D1 to D28) were recorded. No serious adverse events were reported in the 28 days after TETAGRIP® administration in any recipient – healthy volunteer or HIV+ patient, TC or IM administration. Moderate local reactions occurred in 3 TC vaccinated healthy individuals (erythema at D1 in one volunteer and erythema at D3 in another one, erythema and swelling in a third volunteer). Mild local reactions occurred in IM vaccinated subjects. Three moderate systemic reactions were recorded for cohort I subjects, as described for both IM and TC groups in Table 2. Axillary adenopathy occurred in one TC vaccinated subject at D7 and D14 (Table subjects 2).

**Table 2:** Summary of clinical safety.

		Cohort I - Healthy Volunteers			Cohort II - HIV+ volunteers		
	n	12	12	24	12	12	24
	Severity	TC	IM	Total	TC	IM	Total
Local reactions	None	2	7	10	0	5	7
(p = 0.05)	Mild	7	5	11	0	3	5
	Moderate	3	0	3*	6	0	6#
	Severe	0	0	0	0	0	0
Systemic events	None	6	4	10	3	3	6
(p = 0.07)	Mild	4	7	11	2	4	6
	Moderate	2	1	3**	1	1	2##
	Severe	0	0	0	0	0	0

Statistical significance was set at p<0.05.

*Moderate local reactions occurred in 3 TC vaccinated healthy individuals and included erythema at D1 in one volunteer and erythema at D3 in another one. The third volunteer experienced erythema and swelling at D1 and erythema at D3. No local reactions occurred in IM vaccinated subjects.

**Three moderate systemic reactions were recorded for cohort I subjects. The subject receiving IM vaccination experienced pain while breathing after sport accident on D7 and D14. Only volunteer experienced vomiting at D28 after TC vaccination. Axillary adenopathy occurred in one TC vaccinated subject at D7 and D14 and was the only systemic event, which was considered as related to the mode of administration by the investigator.

# Moderate local reactions occurred in 6 TC vaccinated HIV-infected individuals and included erythema at D3 in one volunteer, erythema at D14 in another volunteer as well as itching at D1 and D3 in two subjects. One volunteer experienced Itching at D1 followed by erythema at D3, D7, D14 and D21. One erythema and desquamation in D14, D21 and D28. No local reactions occurred in IM vaccinated subjects.

## Two moderate systemic reactions were recorded for cohort II subjects. Only volunteer reported an upper respiratory tract infection at D28 after IM vaccination. One TC vaccinated individual experienced malaise and myalgia at D1 after vaccination.

There was no significant increase in local adverse events, including erythema, itching, pain, swelling, and axillary node enlargement, or in the incidence of systemic (grade ≥3) adverse events (e.g., fever, myalgia, and diarrhea) in either cohort after TC compared to IM administration.

In Cohort II composed of HIV-infected individuals, moderate local reactions occurred in 6 TC vaccinated subjects. Two moderate systemic reactions were recorded for cohort II subjects, which are all detailed in Table 2.

Overall, the tolerance of the investigational TC route of vaccine administration was good.

### Defect in induction of influenza-specific neutralizing Ab responses by the TC but not by IM vaccination route

Influenza vaccines are designed to induce neutralizing antibody (NAb) responses after IM or subcutaneous (SC) administration in humans. NAb responses are therefore the reference criteria for evaluating the efficacy of influenza and tetanus vaccines. NAb responses were evaluated by an independent National Influenza reference center. Serum samples from D0 to D28 were simultaneously tested for strain-specific inhibition of HAI after TETAGRIP® administration by both routes to the 24 healthy subjects (Table 3) and the 14 HIV+ patients (Table 4).

**Table 3:** Anti-influenza specific neutralizing antibodies in healthy individuals.

	A/NEW CALEDONIA			A/CALIFORNIA		
	TC	IM	*p value*	TC	IM	*p value*
**Prior vaccination – Day 0**						
GMT	15.5	23.1	*0.31*	20	36.8	*0.14*
Seroconversion rate	2/12 (17%)	4/12 (33%)	*0,64*	3/13 (25%)	8/12 (67%)	*0.10*
**Post vaccination - Day 28**						
GMT	15.5	123.8	*<0.001*	20	208.2	*0.001*
Mean GMT increase >2.5	1.0	5.36	*<0.001*	1.0	5.66	*0.003*
Seroconversion rate >70%	2/12 (17%)	10/12 (83%)	*0.003*	3/12 (25%)	9/12 (75%)	*0.003*
Seroconversion rate	0/8 (0%)	5/7 (71%)	*0.007*	0.6 (0%)	1/4 (25%)	*0.40*
Significant increase in Ab titers	0/4 (0%)	3/5 (60%)	*0.017*	0/6 (0%)	6/8 (75%)	*0.01*
Seroconversion rate or significant Increase in Ab titers	0/12 (0%)	8/12 (67%)	*0.001*	0/12 (0%)	7/12 (58%)	*0.005*

Statistical significance was set at p<.05.

**Table 4:** Anti-influenza specific neutralizing antibodies in HIV-infected individuals.

	A/NEW CALEDONIA			A/CALIFORNIA		
	TC	IM	*p value*	TC	IM	*p value*
**Prior vaccination – Day 0**						
GMT	13.4	10.1	*ns*	29.0	19.2	*ns*
Seroconversion rate	0/6 (0%)	0/8 (0%)	*ns*	3/6 (50%)	3/8 (38%)	*ns*
**Post vaccination - Day 28**						
GMT	14.0	49.6	*<0.001*	26.8	129.1	*<0.001*
Mean GMT increase >2.5	1.0	4.9	*<0.001*	0.9	6.7	*0.003*
Seroconversion rate >70%	1/6 (17%)	5/8 (63%)	*0.005*	2/6 (33%)	6/8 (75%)	*0.005*

Statistical significance was set at p<0.05.

Results are reported for the geometric mean titers (GMT) of the A/CALIFORNIA/7/2004 H3N3 and A/NEW CALEDONIA/20/99 H1N1 strains at D14 and D28 in healthy volunteers (Cohort I, Table 3). There were no significant differences at baseline for antibody titers between the two study arms. IM vaccination induced the expected influenza-specific NAb responses to hemagglutinin with 75% seroprotection and seroconversion rates together with a significant increase in the antibody titers at D14, while TC vaccination did not. Indeed, TC vaccination did not induce any detectable influenza-specific NAb responses.

There were no significant differences at baseline for antibody titers between the two study arms in HIV-infected individuals and we found similar results among the cohort of HIV-infected volunteers with the absence of NAb responses in the TC group compared to IM group (Cohort II, Table 4).

Similar differences between the TC and IM groups were observed for the induction of NAb responses against tetanus toxin, as reported in supplemental Table S1. The two study arms (TC versus IM) thus differed significantly for all the NAb parameters we measured.

Thus, we found a striking absence of humoral responses to TC compared with IM vaccination.

### Selective amplification of CD8 T cell responses after TC compared with IM vaccination in healthy individuals

**Figure 1:**
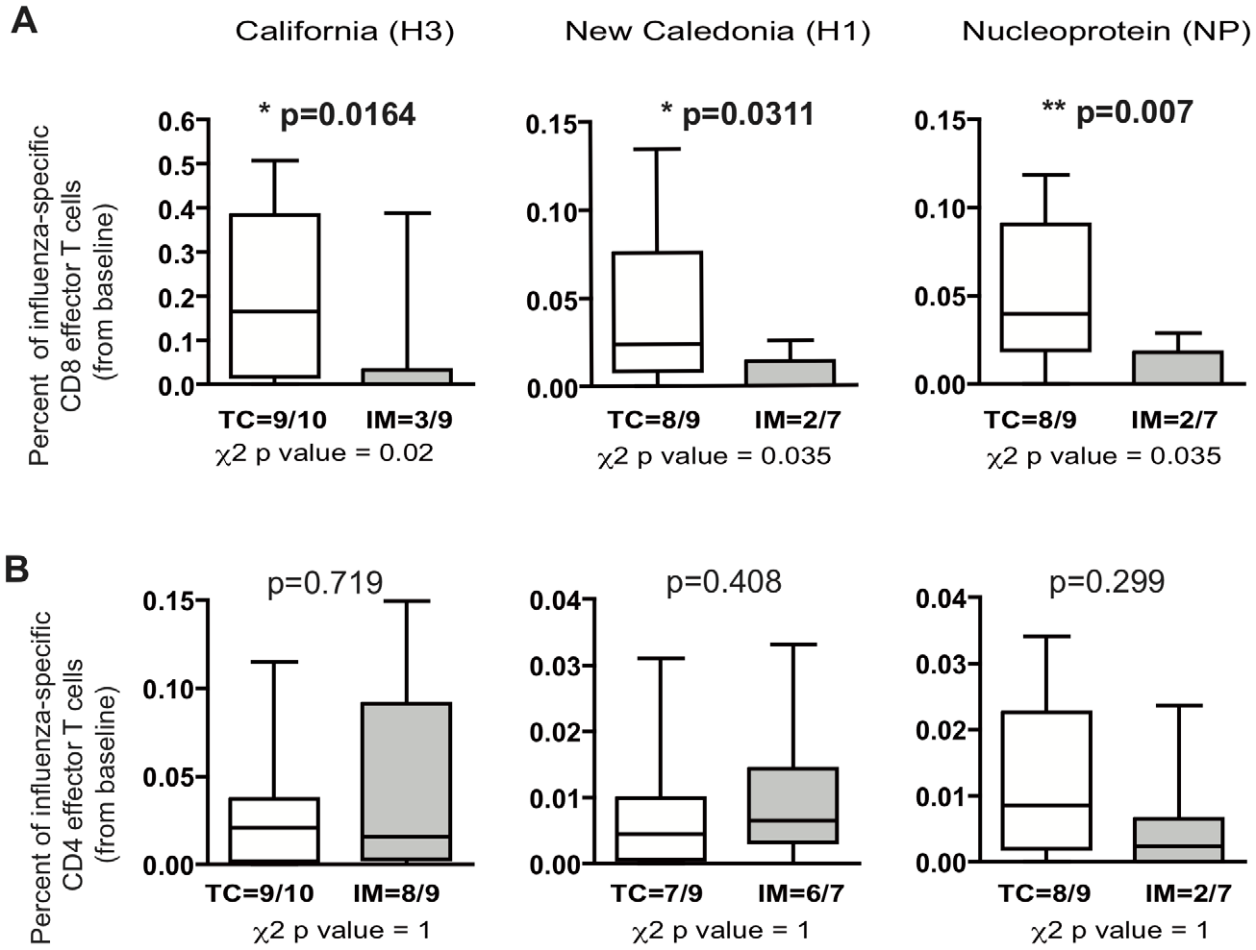
Differential induction of CD4 and CD8 T cell responses after TC vaccine application compared to IM immunization in healthy individuals. Intracytoplasmic cytokine staining (ICS) of influenza-specific effector CD4 and CD8 responses was performed on frozen PBMC samples from vaccinated individuals: 10/12 from the TC group and 7–9/12 from IM group with 90% cell viability after thawing. Three million cells were stimulated with the overlapping peptide covering H3, H1, and NP for 12 hours at 37°C. Brefeldin A was added 4 h before harvesting. ICS was performed by flow-cytometric assays on CD3+CD4+ (left panels) and CD3+CD8+ T cells (Right panels). At least 1,000,000 live events according to forward and side scatter parameters were accumulated and analyzed (M&M section). The expression of IFN-γ, TNF-α, and/or IL-2 (triple+double+single cytokine positive cells) by influenza-specific T cells was analyzed with the Boolean gating function of FlowJo software. Results are shown as percentages of cytokine-producing T cells (Δ Day 28- Day 0) after subtracting the unstimulated cell background. Mann-Whitney test was used to compare continuous variables between the groups. Significance was set at p<0.05. Responders are determined when (Δ Day 28-Day 0) were >0. The χ2 test was used to define categorical variables between TC and IM groups.

**Figure 2:**
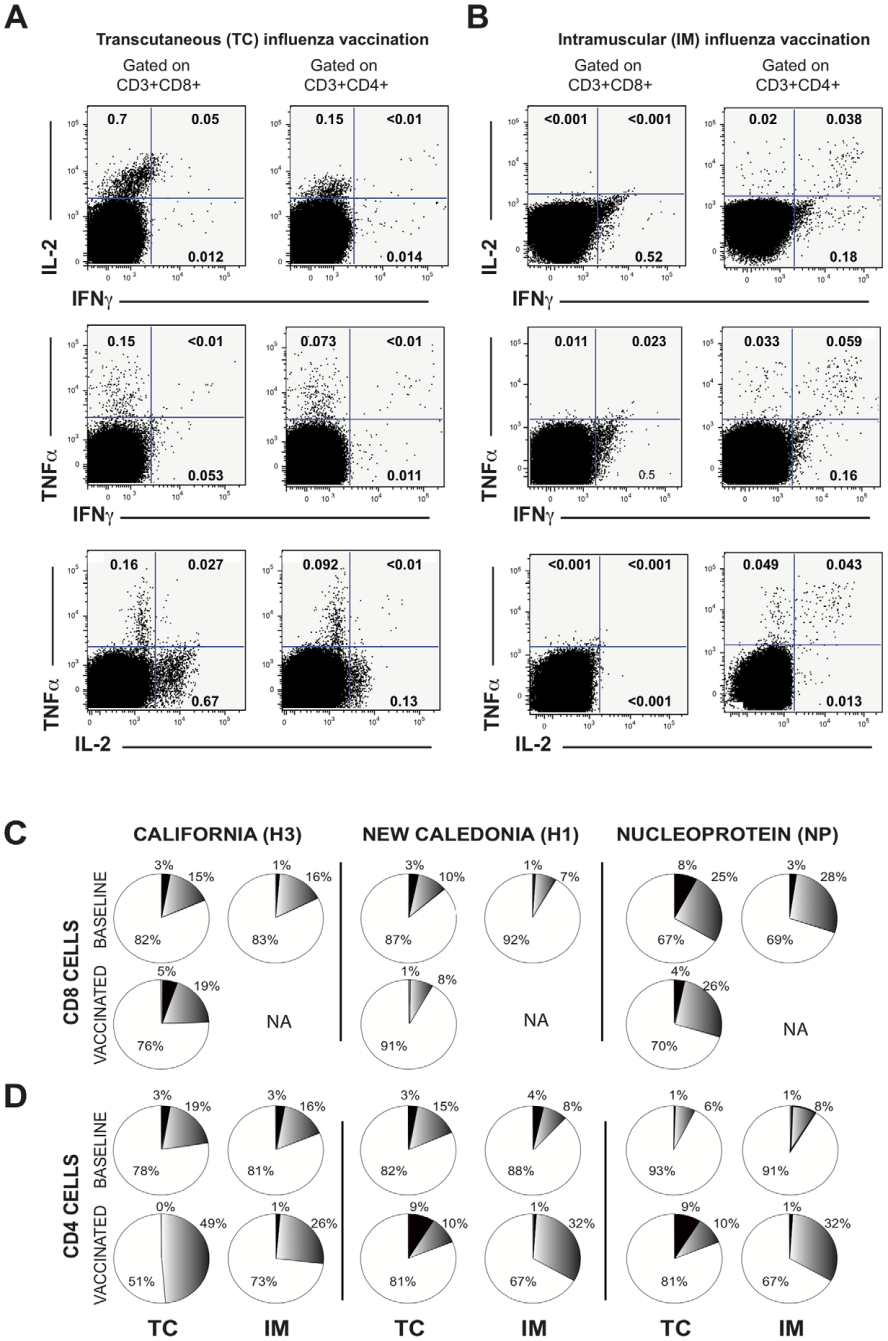
Flow cytometric representation of influenza-specific T cell response at day 28 post-vaccination by TC and IM routes. A, B) Representative flow cytometric analysis of cytokine-producing influenza-specific effector CD4 and CD8 responses. Experiments were performed on frozen PBMCs from individuals vaccinated by TC and IM routes as described in figure 1. Results are shown for a representative healthy individual with a TC route (A) and an IM route (B) vaccination at day 28. C, D) Pie chart analyses of single (white), double (gray) and triple (black)-cytokine positive cells for CD4 (C) and CD8 (D) effector cells specific for the indicated influenza protein. The expression of IFN-γ, TNF-α, and/or IL-2 (triple+double+single cytokine positive cells) by influenza-specific T cells was analyzed with the Boolean gating function of FlowJo software. NA: not applicable.

The epidermal route of immunization described in our study is intended to target mostly epidermal LCs [36]. These APCs have been shown to be more potent in inducing CD8 cells in *in vitro* studies [20]–[23]. In addition, effector CD4 and CD8 cells both provide cellular immune responses and can secrete multiple cytokines that reflect the quality of the effector-cell compartment of the immune responses. Multiparametric flow cytometry assays were performed to determine the relative importance of the subpopulations of influenza-specific CD3+CD4+ and CD3+CD8+ T cells that produce IL-2, IFN-γ, and TNF-α. Overlapping peptides were designed for three major influenza antigens included in the seasonal vaccine: i) H3, a recent strain not contained in seasonal influenza vaccines in Europe over the past five years, ii) H1, repeatedly present in influenza seasonal vaccines over the past five years, and iii) NP. We further analyzed IL-2, IFN-γ, and TNF-α production by CD4 and CD8 cells after *ex vivo* stimulation of T cells with overlapping 20-mer peptides of H3, H1, and NP. Because of the high variability of baseline influenza-specific T cells in healthy individuals, we measured the course of influenza-specific T cell responses from D0. Strikingly, we found that the frequencies of H3-, H1-, and NP-specific CD8 cells producing cytokines (IL-2, IFN-γ, and TNF-α) were significantly higher after TC than after IM vaccination (H3 *p = 0.0164*, H1 *p = 0.031*, NP *p = 0.007*), mainly because the levels of CD8 responses after IM administration was extremely low (Figure 1, upper panels). Influenza-specific CD4 responses were similar for both routes (H3 *p = 0.719*, H1 *p = 0.408*, NP *p = 0.299*). In addition, we found a higher proportion of positive responders after TC compared with IM immunization, as depicted in Figure 1 (upper panels) (χ2 test, H3 *p = 0.02*, H1 *p = 0.035*, NP p = 0.035) for influenza-specific CD8 but not CD4 responses (Fig 2, lower panels). Note the very high frequencies of CD8+cytokine+ cells against H3 proteins after TC vaccination. Overall, we observed preferential induction of CD8 responses against all three protein compounds included in the inactivated influenza vaccine when administered by the TC but not the IM route.

Representative flow cytometric analyses are shown for H3-specific CD3+CD8+ and H3-specific CD3+CD4+ cells by each vaccination route (Figure 2A, 2B). Besides the absence of any increase in influenza-specific CD8 cells after IM vaccination, we also observed significant differences in the distribution of single (SP), double (DP) and triple (TP) cytokine producing CD4 cells on D28 according to the vaccination route. Pie chart analyses summarize the differential distribution of influenza-specific CD4 cells and underline the impact of vaccination route on the quality of vaccine-specific effector T cells (Figure 2C and 2D).

In addition, a tetanus-specific cellular response was tested by IFN-γ-ELISPOT assays, as shown in supplemental Figure S1. Because the cellular response level was below the detection level of the ELISPOT assay, we did not perform further analysis of the quality of the tetanus-specific T cell responses.

**Figure 3:**
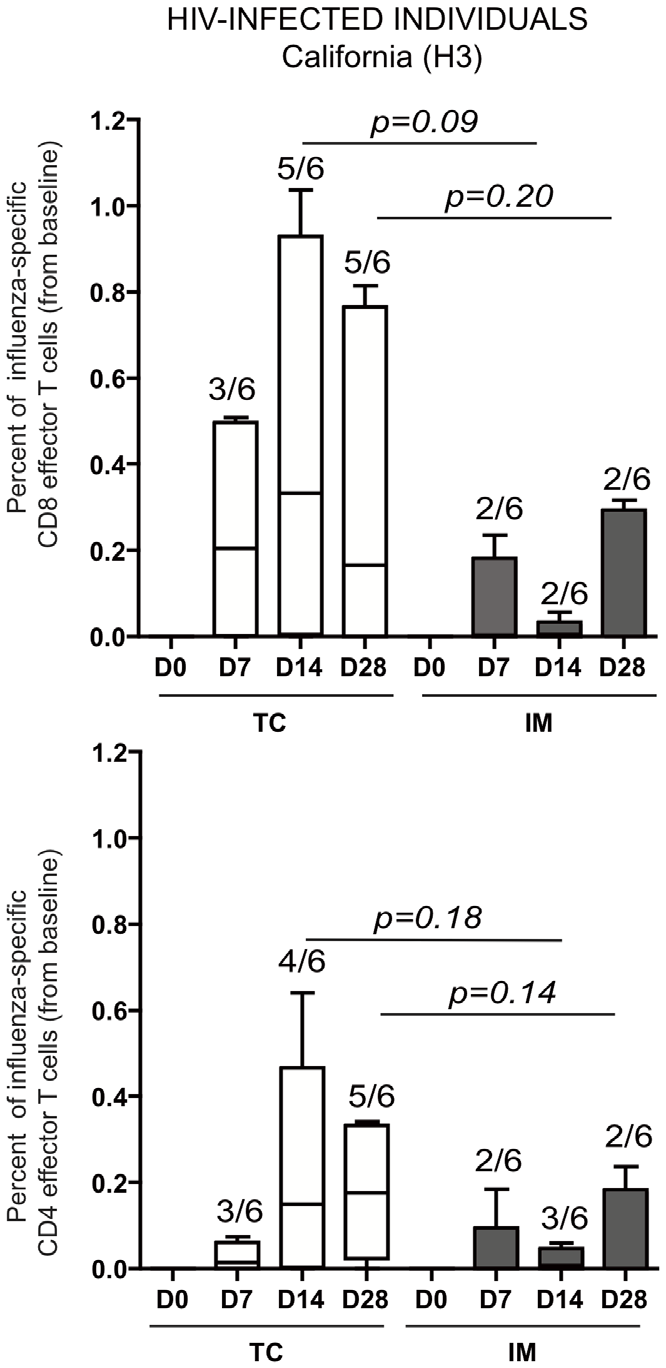
Differential induction of CD4 and CD8 T cell responses after TC and IM vaccine application in HIV-infected volunteers. Intracytoplasmic cytokine staining (ICS) of influenza-specific effector CD4 and CD8 responses was performed on frozen PBMCs from vaccinated individuals. Experiments were performed on 12 HIV-infected individuals for whom cell sample viability was at least 90% after thawing. Three million cells were stimulated with the overlapping peptide covering H3, H1, and NP for 12 hours at 37°C. Brefeldin A (5 µg/ml) was added 4 h before harvesting. ICS was performed for IFN-γ by flow-cytometric assay on CD3+CD4+ (left panels) and CD3+CD8+ T cells (Right panels). At least 1,000,000 live events according to forward and side scatter parameters were accumulated and analyzed (M&M section). The expression of IFN-γ, TNF-α, and/or IL-2 (triple+double+single cytokine positive cells) by influenza-specific T cells was analyzed with the Boolean gating function of FlowJo software. Results are shown as percentages of cytokine-producing T cells (Δ Day 28-Day0) after subtracting the unstimulated cell background. Mann-Whitney tests were used to compare continuous variables between the groups. Significance was set at p<0.05.

Finally, we analyzed the quality of cellular immune responses in HIV+ volunteers (cohort II) after *ex vivo* stimulation of peripheral blood cells with the same overlapping peptides covering H3 (Figure 3), H1, and NP (data not shown). Intracellular cytokine staining assays were performed to assess the production of IL-2, IFN-γ, and TNF-α in CD4 and CD8 cells. We found that H3-specific CD8 cells tended to be slightly more frequent after TC than after IM vaccination in these volunteers (p = 0.09 at day 14 and p = 0.2 at day 28) (Figure 3). This was not the case for H3-specific CD4 responses (p = 0.59), although the small number of individuals tested made it very difficult to obtain significant results (Figure 3). Similar results were observed for H1 and NP proteins (data not shown). Note that the number of HIV+ volunteers able to be tested was reduced because of the short period available until the flu season.

These observations suggest that the effect of the TC route on preferential amplification of vaccine-specific CD8 cells in HIV+ individuals was similar to that in healthy individuals. They need, however, to be confirmed in a larger sample of such subjects. Overall, this result demonstrates for the first time that the route of immunization affects the quality of immune responses and in particular the quality of T cells.

## Discussion

We showed that hair follicular targeting of an inactivated influenza vaccine by the trancutaneous route induced preferentially CD8 effector T cells rather than humoral immune responses when compared to a conventional IM route, in a randomized, controlled Phase I clinical trial in healthy volunteers. Our work is the first demonstration that the immunization route in humans affects the magnitude and quality of CD8 T cell responses as well as the intrinsic quality of cytokine-producing CD4 effector cells after TC and IM vaccination with a seasonal influenza vaccine. We also found similar results after influenza vaccination in HIV-infected patients.

The importance of anti-viral CD8 cells as well as recent influenza pandemics (H5N1 and H1N1swine) have raised the need of re-evaluating cellular responses [39]–[41]. Numerous reviews and studies have highlighted the importance of respiratory virus-specific effector and memory T cell responses in humans and mice [41] that participate to the accelerated viral clearance as well as enhanced survival after viral challenge [41]–[43]. In addition, according to current concepts in HIV vaccine design, a broadly targeting vaccine-specific CD8 response would restrict acute HIV replication [44]–[47]. The HIV-infected population would constitute one of the key populations that would benefit from such novel vaccination strategies.

The aim of this approach is not yet to propose a vaccination strategy for a seasonal influenza epidemic, but rather a proof-of-concept study that the route of immunization will help in directing immune responses towards a larger pool of CD8 responses against infectious diseases. Conventional inactivated influenza vaccine was used as an example for vaccination because of its ease to use and wide acceptability by volunteers for clinical trials. The strength of our work resides on the use of this type of vaccine, that has been designed for antibody responses and not CD8 responses, rather the use of live viruses.

Vaccines are classically injected into a muscle, where the local deposit of vaccine compound must be captured by locally present and recruited APCs. Muscles, however, have very few DCs, and vaccines generally require adjuvant to improve their immunogenicity. Because our method of vaccine application results in opening the hair follicle, it should make skin DCs, including epidermal LCs, which reside in and around the hair follicles, highly accessible to topically applied vaccines [36], [48]. Recent studies suggest that DC subsets play a role in generating adaptive immune responses of quantitatively and qualitatively distinct types [49], [50]. The elicitation of T cell responses by epicutaneous immunization [34], [51], [52] suggests that LCs play a role. It was recently shown that LCs cross-present exogenous antigen to CD8+ T cells in murine models [51], [52]. Dermal DCs (in the dermis) and LCs (in the epidermis) have different migratory paths through the lymphoid tissues [53]. In addition, LCs generated *in vitro* and derived from CD34+ precursors can cross-present exogenous antigen to CD8+ cells [54] more potently than either dermal or monocyte-derived DCs [55]. The efficacy of cellular immune responses also appears to rely on strong antigen-induced priming of T cells by efficient targeting and activation of professional APCs [6], [56].

In this study, vaccine was applied by a two-step protocol that included a single CSSS procedure on the upper arm followed by topical application of a total dose of a conventional influenza/tetanus vaccine. The safety and tolerability of this CSSS procedure in human volunteers was consistent with our previous findings in a pilot study [37] of a limited number of healthy volunteers, where we used CSSS and an anti-influenza vaccine (not combined with any other product). Our first published pilot study was conducted to define the surface area required for successful vaccination by this method [37]. Previous studies have already demonstrated the success of CSSS application in penetration of human skin [57], [58]. This trial, however, is the first to demonstrate the safety of this CSSS procedure in HIV-infected patients. Skin physiology measurements confirmed that the skin parameters we measured did not differ significantly between the two cohorts (healthy volunteers and HIV-infected patients) or the two study arms (TC versus IM administration). The TC vaccination protocol was also safe in this specific patient population.

In recent years, various studies have recognized the feasibility of TC vaccination in mice and even in humans (for review see [24]). Safety and humoral responses to adjuvanted or live-inactivated vaccines have been widely studied in humans, but not the induction of cellular responses. In Phase I/II studies of a live-attenuated measles vaccine in humans, Etchard et al. [59] recently showed that TC – but not subcutaneous – immunization failed to induce serum antibodies and induced only limited mucosal IgA and IFN-γ responses. Yagi and colleagues explored the induction of T cell immune responses in five melanoma patients [60]. They showed that five percutaneous immunizations (monthly application of HIV and melanoma MHC-Class I restricted peptides) allowed the induction of peptide-specific CD8 cells. In that study, CSSS procedures led to complete removal of the stratum corneum, on skin areas as large as 100 cm^2^. That study also used substantially more than our single vaccine application; however, we showed here that one application of influenza vaccine is sufficient to induce cellular responses by the TC route. Further dose-dependent vaccination needs to be performed in the future.

We previously explored the induction by TC and IM vaccination of cellular immune responses in 11 healthy volunteers (n = 6 TC and n = 4 IM) by a seasonal anti-influenza vaccine [37]. Interestingly, TC vaccination induced both CD4 and CD8 T cell responses, whereas IM injection induced only effector CD4 T cells. Nonetheless, the small sample size prevented effective statistical analysis. The Phase I trial reported here showed a significant difference according to route of administration in the magnitude of the CD8 effector T cell population directed against three distinct influenza vaccine proteins: hemagglutinins H3 and H1 and NP. CD4 and CD8 cell response against the influenza virus have been thoroughly described. It has been shown that CD8 cells make it possible to eliminate the virus [5], [61]–[64] as well as to control secondary infection by a lethal influenza strain in the absence of B cells and antibodies [65], [66]. CD8 cells specific for influenza proteins may recognize viral epitopes such as nucleoprotein (NP), polymerase acid (PA), matrix protein (M), and nonstructural proteins [67]–[71]. In influenza infections in murine models, protection by CD8 T cells has been shown to be derived from restricting the dissemination of influenza A as well as of influenza variants [72]–[76]. In elderly humans, cellular immune responses against influenza correlated with protection against influenza virus and thus indicated the limitations of using serum antibody responses alone to measure vaccine efficacy [77], [78].

Beyond the importance of CD8 immune responses in pandemic influenza and vaccination in the elderly and other immunocompromised individuals, the magnitude of antigen-specific T cell responses is a measure of vaccination efficacy against viral diseases and also cancers. Efforts to improve vaccination efficacy will help in the fight against these diseases. However, it does not reflect the functional abilities that can be analyzed for an antigen-specific T cell population. Currently flow cytometric techniques allow the analysis of multiple functions. IFN-γ, TNF, and IL-2 are analyzed most often to assess cellular immunity to infectious diseases [79], [80]. It has been suggested that the quality of T cells is crucial for determining the outcome of infectious diseases [10]. However, quality is a critical point that has not yet been defined in terms of control of viral diseases. Most reports of the multifunctionality of T cells are based on studies of HIV infection or vaccination, and it remains difficult to expand or generalize these results to other infectious diseases or vaccinations. Combinations of markers can paint a more detailed picture of antigen-specific T cells. We found that the quality of polyfunctional CD8 and CD4 effector/memory cells against influenza proteins (H3, H1, and NP) was similar at baseline in both arms of the study and thus confirmed the relative homogeneity of influenza-specific effector/memory cells before vaccination. The TC and IM routes of vaccination that induced differential cytokine profiles that suggested that the quality of CD4 cell stimulation by these routes might differ. The induction of CD8 responses after IM vaccination was extremely limited and did not allow further analysis.

Nevertheless, vaccination by the TC route did not induce NAb responses. This result may also be related in part to the strength of immunization or the differential quality of the APCs, which can dictate immunological outcome.

A major obstacle to skin vaccine delivery is the stratum corneum, an important constituent of the skin barrier. Multiple approaches have explored ways of overcoming it. Interestingly, Fan et al. found that topical vaccination requires the presence of intact hair follicles, which are the most relevant physiological breaks in the skin barrier. They also highlighted the operation of efficient mechanisms within the follicle for the induction of immune responses against DNA vaccines [81]. Numerous studies have recognized the importance of hair follicles in percutaneous penetration processes [36], [38], [82], [83]. The CSSS technique facilitates follicular penetration by removing cellular debris and sebum from the hair follicle openings [82], [83]. In contrast to previous approaches, however, CSSS as it was used in this study had two important effects on the skin. It removed approximately 30% of the stratum corneum, inducing mild barrier disruption without damaging the viable epidermis and its associated cell populations (LCs), and it removed cellular debris from hair follicles, hereby increasing the number of hair follicles available for penetration. This idea of utilizing the reservoir function of the hair follicle for TC vaccination strategies is perfectly in line with recent reports by Naito *et al*, who found that prolongation of antigen presence increased the efficacy of TC immunization in mice [84]. While it remains difficult to evaluate the amount of antigen to which the immune system is actually exposed, this study supports the experimental evidence from many studies that TC vaccination may in fact allow dose sparing with equivalent T cell responses, self-administration, and immune enhancement, even in elderly patients with less responsive immune systems [85], [86].

## Materials and Methods

### Study Design

The primary objective of this study was to evaluate the safety of a newly developed protocol for TC vaccine application in healthy volunteers (cohort I) and HIV-infected individuals (cohort II) by using a commercially available tetanus/influenza vaccine (Tetagrip®), in comparison to conventional IM injections. The secondary objectives included first a comparison of the immunogenicity of TC and IM vaccination by assessing the tetanus- and influenza-specific antibody titers and cellular immune responses, and second, an evaluation of skin physiology in the patients in the TC groups. The study included two cohorts of volunteers composed of healthy individuals (cohort I, n = 24), and HIV-infected patients (cohort II, n = 14), each randomized in two arms *i.e.* TC versus IM vaccination, of 12 subjects per arm, as defined by the Data Management and Statistics Center, according to an SAS procedure plan. The volunteers were recruited at two investigational centers (Department of Dermatology and Allergy, Charite – Universitaetsmedizin Berlin, Germany, and HIV Treatment & Research Unit, Department of Internal Medicine II, Johann Wolfgang Goethe University Hospital, Frankfurt, Germany). After vaccination at D0, safety data were collected at D1, D3, D14, D21, D28, and blood samples were taken for immunological analyses at indicated time points (D0, D14 and D28). Skin physiological measurements were performed before and after vaccination at indicated time points. The protocol for this trial and supporting CONSORT checklist are available as supporting information (Checklist S1, Flowchart S1 and Protocol S1).

### Ethics committee approval, health authorities

The trial was conducted in accordance with the latest Declaration of Helsinki, GCP, and ICH regulatory guidelines. The study protocol, its first amendment, and the informed consent and patient information forms were reviewed and approved by the independent Ethics Committee of Charite – Universitaetsmedizin Berlin, Campus Mitte, and submitted to the Ethics Committee of Johann Goethe University, Frankfurt, Germany. Approval was further obtained from the Paul-Ehrlich Institute, Germany (Federal Agency of Sera and Vaccines). Written informed consent was obtained from each volunteer before study entry. The clinical trial has the following identification number NCT00261001.

### Study population

Volunteers from both cohorts had to meet the following eligibility criteria: men aged 18–45 years, body mass index (BMI) 21–26, skin phototype I–IV, clinical examination and interview for medical history, no tetanus vaccination within the past four years, no influenza vaccination within the past year, no psychological, familial, sociological or geographic condition that might impede compliance with the study protocol, and written informed consent. Men with excessive terminal hair growth on the investigational sites were excluded from the study. Further exclusion criteria were any acute or chronic illness, skin conditions or allergies, or local or systemic treatments that might interfere with the study protocol, and past or planned sessions of ultraviolet or sun exposure within six weeks of study entry. Healthy volunteers with a negative HIV test within three months of study entry were included in cohort I. Inclusion in cohort II (HIV-infected patients) required HIV-infection, positive HIV serology, CD4 cell nadir >200/mm3, effective antiretroviral treatment with a minimum of three drugs for <1 year prior to study entry with a plasma HIV RNA <400cp/ml during the 6 months before the inclusion and CD4+ counts >350 cells/mm3 for 12 months.

### Vaccine

TETAGRIP® is a commercially available influenza/tetanus vaccine (Sanofi-Pasteur, France). The vaccine is provided as injectable suspension of one vaccine dose containing tetanus anatoxin and inactivated influenza virus type A and type B fragments of influenza antigens equivalent to 15 µg of 2 type-A (A/CALIFORNIA/7/2004 H3N3 and A/NEW CALEDONIA/20/99 H1N1) and 1 type-B (B/SHANGAI/361/2002) virus hemagglutinin subunits in saline solution. For TC and IM vaccination, 0.5 ml of the vaccine was used, as provided by the manufacturer.

### TC and IM vaccination

TC vaccination was performed as described elsewhere, with a newly developed standard operating procedure based on CSSS [36], [37]. Briefly, two investigational sites of 4×4 cm each were delimited on the external part of the upper left arm with a permanent skin marker (Skin marker H7003 Falc). The investigational sites and the surrounding skin (2 cm on the top and bottom of each investigational site and 1 cm on both sides) were lightly shaved with a dry razor (Disposable razor, Art.-No. 182 H, Wilkinson Sword GmbH, Germany). One CSSS was performed with 190 mg cyanoacrylate (Superglue, UHU GmbH & Co. KG, Germany), which was spread evenly on the skin surface with a microscope slide. Adhesive tape (6×5 cm, Art.-No. 571176-00000, Tesa Beiersdorf, Germany) was then applied and massaged with a rubber roll to improve adhesion (10 times). After the glue hardened for 20 minutes, tape and glue were removed from the skin surface. A silicone barrier was placed to protect the area around the investigational sites (Window-Colourpaste, Art.-No. 4469/ko, Max-Bringmann GmbH & Co., Wendelstein, Germany) to avoid spreading the vaccine. TETAGRIP® vaccine (250 µl) was applied in droplets from the original syringe provided by the manufacturer onto the skin surface of each investigational site (16 drops per investigational site, each drop approximately 16 µl) and then gently massaged with gloved fingertip (care & serve®) presaturated with vaccine for one minute. After an incubation time of 20 minutes, a protective hydrocolloid bandage (Comfeel® Plus Transparent 9×14 cm Art.-Nr.: 3542, Coloplast A/S, Denmark) was applied to the dried surface to protect the investigational sites for 24 h. The volunteers were instructed not to take a shower or bath and to avoid any activity that caused sweating or mechanical stress to the investigational site, e.g., physical exercise, for these 24 hours.

For the IM group, TETAGRIP® vaccine (0.5 ml) as provided by the manufacturer was injected intramuscularly into the deltoid muscle of the left arm after careful disinfection, following the most recent ICH Good Clinical Practices.

### Clinical safety

After each immunization the volunteers remained under medical supervision for at least 30 minutes. Safety data (local and systemic reactions, adverse events) were assessed and recorded on D1, D3, D7, D14, D21, and D28. Each visit included an interview, review of the diary cards, and a physical examination of the volunteer. Local reactions were graded 0–3 (0 = none, 1 = mild, 2 = moderate, 3 = severe) according to the occurrence of erythema, pruritus, burning, or desquamation. Systemic reactions (e.g., rash, pain, fever, headache, shivering, diarrhea, or malaise) were similarly graded 0–3. Severe adverse events were graded 1–4 for each organ system according to clinical and laboratory parameters defined in standard toxicity tables.

### Skin physiology measurements

Transepidermal water loss (TEWL), stratum corneum hydration, skin pH, and sebum production were assessed before vaccination on D0 with a Multi Probe Adapter MPA® (Courage-Khazaka, Cologne, Germany). All skin measurements were performed according to the manufacturer's recommendation.

### HAI titers

Serum antibody against influenza was measured at the Institut Pasteur, the French national reference for influenza (Centre National de Référence de la Grippe) by a standard microtiter hemagglutination inhibition (HAI) assay, as previously described [87]. HAI antibody titers were determined before and after influenza vaccination in all volunteers. Serum samples from D0, D14, and D28 were simultaneously tested for strain-specific HAI Results are reported for the A/CALIFORNIA/7/2004 strain. The humoral response to this strain contained in the influenza vaccine was assessed by calculating the geometric mean titers before vaccination and at D14 and D28, and the fold increases in the titer at D14 and D28.

### Synthetic peptide design

Fifty-six overlapping 20-mer peptides covering the entire H1 hemagglutinin protein (strain A/NEW/CALEDONIA/20/99) – included in European influenza vaccines over the past five years – were synthesized (Altergen, France), and a super pool was generated. Overlapping 20-mer peptides [88] (32 for H3 and 7 for nucleoprotein, NP) covering MHC Class I and II peptides of the H3 hemagglutinin protein (strain A/CALIFORNIA/7/2004) (not included in European influenza vaccines over the past five years) and NP (strain A/NEW/CALEDONIA/20/99) were designed based on epitopes described in the literature for 48 H3 and 44 NP peptides. To visualize the protein region rich in T cell epitopes, sequences were aligned (Multalin® Software, INRA, France) between the H3 hemagglutinin protein (strain A/CALIFORNIA/7/2004) and all related T cell epitopes, thus refining the 20-mer overlapping peptides. All peptides were synthesized by Eurogentec, France.

### Intracellular cytokine staining

Immunomonitoring studies were performed for the number of individuals indicated below, that is, for the subjects whose frozen blood samples were properly stored and had a cell viability superior to 90% on thawing. All experiments were performed blinded to study arm. Experiments were performed on frozen PBMC samples from most subjects in both cohorts: i) in cohort I, 10/12 members of the TC group and 7 to 9/12 members of the IM group and ii) in cohort II, 6/6 of the TC group and 6/8 of the IM group. Frozen PBMCs were thawed in RPMI (Life Technologies, France) containing 5% FCS (Seromed, Germany), 2 mmol/l L-glutamine (Gibco BRL, Life Technology, Scotland), and antibiotics (1000 UI/ml penicillin sodium, 1 mg/ml streptomycin sulfate, and 250 ng/ml amphotericin B). Cells were stimulated with three different pools of 20-mer peptides (2 µg/ml) for H1, H3, and NP antigens. Brefeldin A (5 µg/ml) (Sigma Chemical Co., France) was added to the well four hours before harvesting to detect intracellular cytokines. Then cells were stained in PBS 1× for 10 min at RT, and membrane markers were added for 20 min at 4°C. Next 100 µl of Fix and Perm Medium A (Caltag, France) was added to each sample for 10 min at RT. Cells were washed, resuspended with 100 µl of Fix and Perm Medium B (Caltag, France), and incubated with intracellular monoclonal antibodies (Abs) specific for cytokine detection for 20 min at RT. The following panel of eight Abs was used: CD4-AmCyan, CD8-PacificBlue, CD27-APC, CD45RA-ECD, IL2-FITC, IFN-γ-Alexa700, TNF-α-PE-C7. Flow cytometric analyses were done with LSRII flow cytometers (Becton Dickinson, Immunocytometry Systems). Analyses were performed with FlowJo software (Tree Star). The live lymphocyte gate (at least 1,000,000 live events) was set based on forward and side scatter for further analysis. The expression of IFN-γ, TNF-α, and IL-2 (triple+double+single cytokine-positive cells) by influenza-specific T cells was analyzed with the Boolean gating function of FlowJo software as described previsouly [89], [90], [91]. Results are shown as percentages of cytokine-producing T cells (Δ Day 28-Day 0) after subtracting the background unstimulated cells. Responders are determined when (Δ Day 28-Day 0) were superior to 0.

### Statistical Analysis

The data analysis consisted of a comparison of safety and immunogenicity data between study arms. All statistical analysis was performed using SSPS 11 software or Prism 4.0c for Mac OS X for data handling and graphic representation. The analysis variables consisted of baselines variables, primary endpoints (safety variables), and secondary endpoints (immunogenicity variables). For baseline variables, descriptive analyses were performed (%, median, interquartiles, ranges) by arm in each cohort. For safety and immunogenicity the two arms were compared using non-parametric tests: Fisher exact test for qualitative variables, Mann-Whitney tests for continuous variables and χ2-test for categorical variables. Statistical significance was set at p<0.05.

## Supporting Information

Table S1 Supplemental Data Table.
(0.04 MB DOC)

Figure S1 Tetanus-specific cellular immune responses after TC and IM vaccination.
(0.12 MB TIF)

Protocol S1 Trial Protocol.
(0.65 MB PDF)

Checklist S1 Consort Checklist.
(0.19 MB DOC)

Flowchart S1 Flowchart cohort I and cohort II.
(0.11 MB PDF)
