# Supplementary material for: Preferential Amplification of CD8 Effector-T Cells after Transcutaneous Application of an Inactivated Influenza Vaccine: A Randomized Phase I Trial
Source: PLoS One. 2010 May 26;5(5):e10818. doi: 10.1371/journal.pone.0010818 (PMC2877091; doi:10.1371/journal.pone.0010818)
Supplement: Table S1 — Supplemental Data Table. (0.04 MB DOC) [file pone.0010818.s001.doc]

# Supplemental Data

**Table S1: Anti-Tetanus-Specific Neutralizing Antibodies in Healthy Individuals After TC and IM Vaccination**

| Neutralizing Abs  Anatoxin Titer (UI/ml)  n (% responders) | Transcutaneous | Intramuscular | Transcutaneous | Intramuscular |
| --- | --- | --- | --- | --- |
|  | D0 | | D28 | |
| Weak (vaccination is advised) | 0 (0%) | 2 (16.7%) | 0 (0%) | 0 (0%) |
| Good Titer (2 years control) | 3 (25%) | 4 (33 .3%) | 2 (16.7%) | 0 (0%) |
| Good Titer (5 years control) | 9 (75%) | 6 (50.0%) | 10 (83.3%) | 12 (100%) |
| Good Titer (7 years control) | 0 (0%) | 0 (0%) | 0 (0%) | 0 (0%) |
| *P value** | *0.25* | | *0.48* | |

Antibodies against Tetanus toxoid were measured by ELISA at the Laboratoire de Microbiologie (Hôpital Henri Mondor, France) as previously described (Comparison of Enzyme-Linked Immunosorbent Assay and Passive Hemagglutination Method for Quantification of Antibodies to Lipopolysaccharide and Tetanus Toxoid in Rats. J. G. Vos, J. Buys, J. G. Hanstede, and A. M. Hagenaars). *Mann-Whitney tests were used to compare continuous variables between TC and IM groups. Statistical significance was set at p<0.05.
