# Supplementary figures and images for: Preferential Amplification of CD8 Effector-T Cells after Transcutaneous Application of an Inactivated Influenza Vaccine: A Randomized Phase I Trial
Source: PLoS One. 2010 May 26;5(5):e10818. doi: 10.1371/journal.pone.0010818 (PMC2877091; doi:10.1371/journal.pone.0010818)

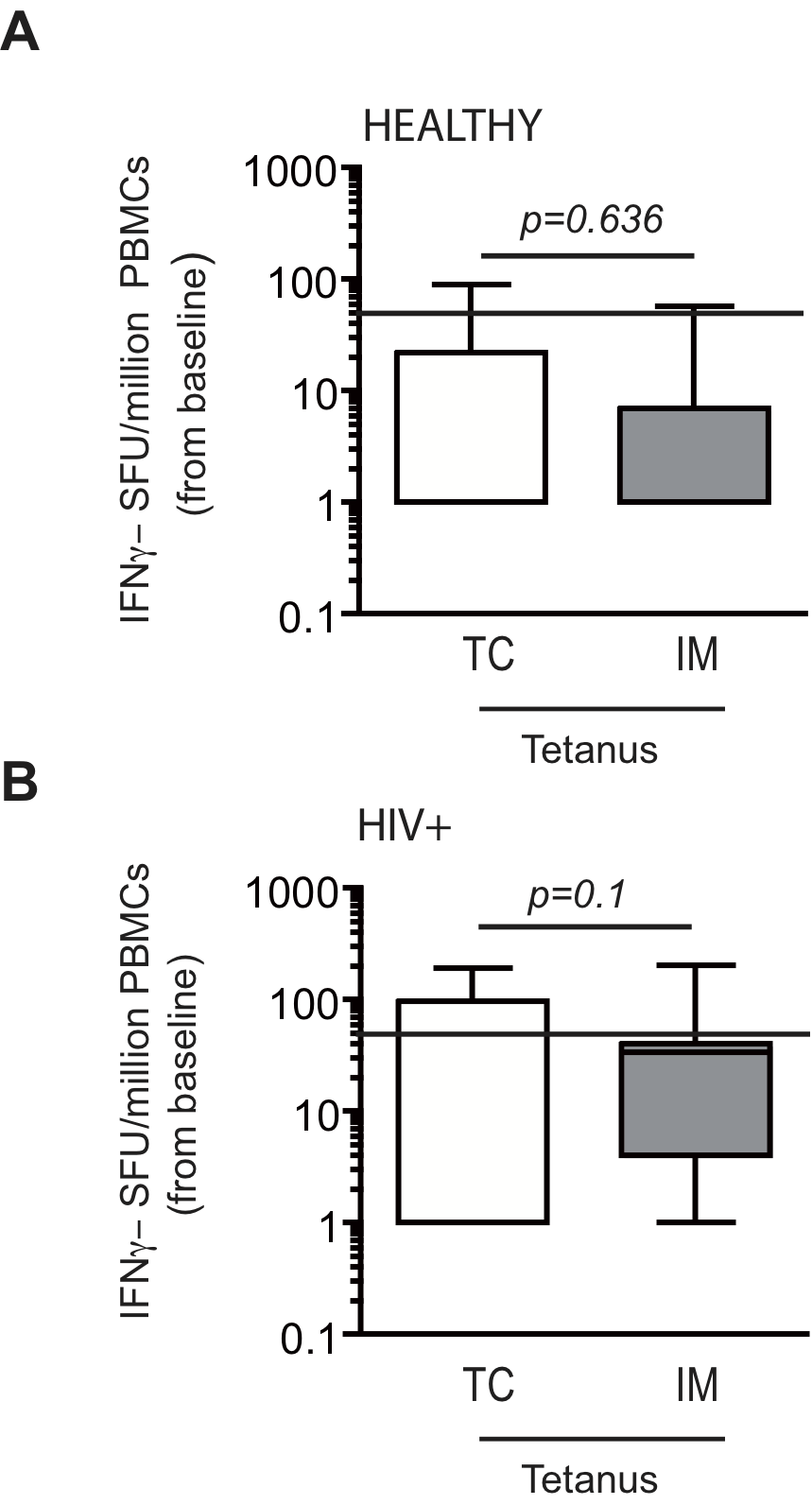

Supplement: Figure S1 — Tetanus-specific cellular immune responses after TC and IM vaccination. (0.12 MB TIF) [file pone.0010818.s002.tif]
