# Supplementary material for: Preferential Amplification of CD8 Effector-T Cells after Transcutaneous Application of an Inactivated Influenza Vaccine: A Randomized Phase I Trial
Source: PLoS One. 2010 May 26;5(5):e10818. doi: 10.1371/journal.pone.0010818 (PMC2877091; doi:10.1371/journal.pone.0010818)
Supplement: Protocol S1 — Trial Protocol. (0.65 MB PDF) [file pone.0010818.s003.pdf]

Objectif Recherche Vaccins SIDA
Hôpital Pitié-salpêtrière
47-83, boulevard de l'hôpital
75651 paris

CLINICAL STUDY PROTOCOL

MANON 05 – CUTAVAC

A RANDOMIZED COMPARATIVE PHASE I STUDY TO EVALUATE AND TO COMPARE THE SAFETY AND IMMUNOGENICITY OF A TRANSCUTANEOUS MODE OF ADMINISTRATION OF A LICENSED TETANUS / INFLUENZA VACCINE TO THE CONVENTIONNAL INTRAMUSCULAR ROUTE OF VACCINE ADMINISTRATION IN HEALTHY VOLUNTEERS AND HIV-INFECTED PATIENTS

EUDRACT NUMBER:

**Protocol Chairman**	Pr Christine KATLAMA, MD
**Protocol Co-Chairman**	Pr Brigitte AUTRAN, MD PhD
**CLINICAL CENTERS IN GERMANY**	
**Study Principal Investigator**	Pr Ulrike BLUME-PEYTAVI, MD PhD
**Study Investigator**	Pr Schlomo STASZEWSKI, MD
**CLINICAL CENTER IN FRANCE**	
**Study Principal Investigator**	Pr Christine KATLAMA, MD
**Immunology Study Coordinator**	Pr Brigitte AUTRAN, MD PhD
**Data Management and Statistics**	Dominique COSTAGLIOLA, PhD
**Project Leader**	Norma WINCKER, PhD
**Sponsor**	ORVACS

Final Protocol Version date: August 08th, 2005

The information contained in this document is confidential and is the property of the sponsor. This information is given for the needs of the trial and must not be disclosed without prior written consent of the sponsor. Persons to whom this information is given for the needs of the trial must be informed that it is confidential and must not be disclosed.

PROTOCOL AUTHORS AND SPONSOR SIGNATORIES

**PROTOCOL AUTHORS**	**Date**	**Signature**
**PROTOCOL CHAIRMAN AND SPONSOR REPRESENTATIVE**		
C KATLAMA, MD	______________	____________________
**PROTOCOL CO-CHAIRMAN AND IMMUNOLOGY STUDY COORDINATOR**		
B. AUTRAN, MD PHD	______________	____________________
**CLINICAL STUDY PRNCIPAL INVESTIGATOR IN GERMANY**		
U.BLUME-PEYTAVI, MD PHD	______________	____________________
**CLINICAL STUDY INVESTIGATOR**		
S STASZEWSKI, MD	______________	____________________
**CLINICAL STUDY PRINCIPAL INVESTIGATOR IN FRANCE**		
C KATLAMA, MD	______________	____________________
**PROJECT LEADER**		
N. WINCKER, PHD	______________	____________________
**DATA MANAGEMENT AND STATISTICS**		
D. COSTAGLIOLA, PHD	______________	____________________
**SPONSOR SIGNATORIES**		
PRESIDENT: GILLES BRUCKER, MD	______________	____________________
CLINICAL MANAGER: CHRISTINE KATLAMA, MD	______________	____________________

SIGNATURE FORM

PROTOCOL MANON 05-CUTAVAC

Sponsor's representative:

Christine Katlama, MD Date: ___________ Signature: _________________

Investigator :
I have read the protocol of the following study:

A randomized comparative Phase I study to evaluate and to compare the safety and immunogenicity of a transcutaneous mode of administration of a licensed Tetanus / Influenza vaccine to the conventional intramuscular route of vaccine administration in healthy volunteers and HIV-infected patients/ **Protocol MANON 05 – CUTAVAC**

I agree:

- to conduct the study as outlined in the protocol and in compliance with GCPs and with applicable regulatory requirements;
- to provide the protocol and all investigational drug and administration mode information relating to pre-clinical and prior clinical experience, if any furnished to me by the sponsor, to all physicians responsible to me who participate in this study. I will discuss all information with them to assure that they are fully informed regarding the administration mode, drug and the conduct of the study;
- to appropriately direct and assist the staff under my control, who will be involved in the study;
- to use the trial material including drug supplies only according to the instructions of the protocol;
- to permit monitoring, auditing and inspection;
- to retain the trial-related essential documents until the Sponsor informs me that these documents are no longer needed;

I have been informed that certain regulatory authorities require the Sponsor to obtain and supply details about the investigator's ownership interest in the Sponsor or the experimental mode of administration or the study drug, and more generally about his/her financial ties with the Sponsor. ORVACS will use and disclose the information solely for the purpose of complying with regulatory requirements.

Hence I:

Agree to supply ORVACS with any information regarding ownership interest and financial ties connected with the study or with ORVACS.

Agree to promptly update this information if any relevant changes occur during the study; and

Agree that ORVACS may disclose this information about such ownership interests and financial ties to regulatory authorities.

Investigator name _______________________

Investigator signature _______________________ Date: ____________

SIGNATURE FORM

PROTOCOL MANON 05-CUTAVAC

Sponsor's representative:

Christine Katlama, MD **Date:** ___________ **Signature:** _________________

Co-Investigator : (*member of the clinical team designed and supervised by the investigator to perform critical trial-procedures and/or to make important trial-related decisions)*

I have read the protocol of the following study:

A randomized comparative Phase I study to evaluate and to compare the safety and immunogenicity of a transcutaneous mode of administration of a licensed Tetanus / Influenza vaccine to the conventional intramuscular route of vaccine administration in healthy volunteers and HIV-infected patients/ **Protocol MANON 05 – CUTAVAC**

I agree:

- to conduct the study as outlined in the protocol and in compliance with GCPs and with applicable regulatory requirements;

- to provide the protocol and all investigational drug and administration mode information relating to pre-clinical and prior clinical experience, if any furnished to me by the sponsor, to all physicians responsible to me who participate in this study. I will discuss all information with them to assure that they are fully informed regarding the administration mode, drug and the conduct of the study;

- to appropriately direct and assist the staff under my control, who will be involved in the study;

- to use the trial material including drug supplies only according to the instructions of the protocol;

- to permit monitoring, auditing and inspection;

- to retain the trial-related essential documents until the Sponsor informs me that these documents are no longer needed;

I have been informed that certain regulatory authorities require the Sponsor to obtain and supply details about the investigator's ownership interest in the Sponsor or the experimental mode of administration or the study drug, and more generally about his/her financial ties with the Sponsor. ORVACS will use and disclose the information solely for the purpose of complying with regulatory requirements.

Hence I:

Agree to supply ORVACS with any information regarding ownership interest and financial ties connected with the study or with ORVACS.

Agree to promptly update this information if any relevant changes occur during the study; and

Agree that ORVACS may disclose this information about such ownership interests and financial ties to regulatory authorities.

Co-investigator name _______________________

Co-investigator signature _______________________ Date: ____________

INVESTIGATIONAL SITE I
Cohort I

Charité – Universitätsmedizin Berlin
Department of Dermatology and Allergy
Clinical Research Center for Hair and Skin Physiology
Schumannstr. 20/21
10117 Berlin - GERMANY

Principal Clinical Investigator
Ulrike BLUME-PEYTAVI, MD PhD
Tel. : 00 49 30 450 518 229
Fax: 00 49 30 450 518 952
e-mail: ulrike.blume-peytavi@charite.de

Co-Investigators
Annika VOGT, MD
Gregor SCHÄFER, MD
Tel. : 00 49 30 450 518 122
Fax: 00 49 30 450 518 952
e-mail: annika.vogt@charite.de
e-mail: gregor.schaefer@charite.de

Study Technician
Sabrina HADAM

Study Nurse
Martina SCHULTE

INVESTIGATIONAL SITE II
Cohort II

Hôpital Pitié Salpêtrière
Service des Maladies Infectieuses
Pavillon Laveran
47-83 Bd de l'hôpital
75651 Paris - FRANCE

Principal Clinical Investigator
Christine KATLAMA, MD
Tel : 00 33 1 42 16 01 42
Fax : 00 33 1 42 16 01 26
e-mail:christine.katlama@psl.ap-hop-paris.fr

Study Technician
Michèle PAUCHARD

INVESTIGATIONAL SITE III
Cohort II

Hospital of the Johann Wolfgang Goethe-University Frankfurt am Main
HIV Treatment & Research Unit
Department of Internal Medicine II
Theodor-Stern-Kai 7
60590 Frankfurt am Main -GERMANY

Clinical Investigator
Schlomo STASZEWSKI, MD
Tel.: 0049 69 6301 7688
Fax: 0049 69 6301 5712
e-mail : stasz@hivcenter.de

Co-Investigators
Carsten ROTTMANN, MD
Nari OH, MD
Tel.: 0049 69 6301 7680
Fax: 0049 69 6301 5712
e-mail: rottmann@hivcenter.de

Study Technician
Anja VON NESSEN

Study Nurse
Stefan MERTENS

IMMUNOLOGICAL ANALYSIS SITE

Laboratoire d'Immunologie
Cellulaire et Tissulaire
Université Pierre et Marie Curie
Bat. CERVI Hôpital Pitié Salpêtrière,
83 Bd de l'Hôpital,
75013 Paris - FRANCE

Immunology Study Coordinator
Brigitte Autran, MD, PhD
Tel : 00 33 1 42 17 74 81 /03
Fax : 00 33 1 42 17 74 90
e-mail :
brigitte.autran@psl.ap-hop-paris.fr

Immunology Investigators
Béhazine Combadière, PhD
Brice Mahé, MS

Immunology Technicians
Sophie HILPERT
Cecile GAMEIRO

STUDY MONITORING AND DATA MANAGEMENT

Study Monitor
ORVACS
Tchadie BOMMENEL
Université Paris VI - U-720-INSERM
56 Bd. V.Auriol, BP 335
75625 Paris – FRANCE
Tel : 00 33 1 42 16 42 59
Fax : 00 33 1 42 16 42 61
e-mail: tchadie.bommenel@ccde.chups.jussieu.fr

Data Management and Statistics
Dominique COSTAGLIOLA, PhD
Université Paris VI - U-720 INSERM
56 Bd. V.Auriol, BP 335
75625 Paris – FRANCE
Tel : 00 33 1 42 16 42 82
Fax : 00 33 1 42 16 42 61
email:Dominique.Costagliola@ccde.chups.jussieu.fr

SPONSOR REPRESENTATIVES

President
Gilles BRUCKER, MD
Institut de Veille Sanitaire
12, rue du Val d'Osnes
94415 St Maurice cedex
Tel : 00 33 1 41 79 67 05
Fax : 00 33 1 41 79 67 67
e-mail:g.brucker@invs.sante.fr

Clinical Manager
Christine KATLAMA, MD
Hôpital Pitié Salpêtrière
Service des Maladies Infectieuses
Pavillon Laveran
47-83 Bd de l'hôpital
75651 Paris Cedex 13
Tel : 00 33 1 42 16 01 42
Fax : 00 33 1 42 16 01 26
e-mail:christine.katlama@psl.ap-hop-paris.fr

Project Leader/Study Manager
Norma WINCKER, PhD
Hôpital Pitié Salpêtrière
Pavillon Laveran
47-83 Bd de l'hôpital
75651 Paris Cedex 13
Tel : 00 33 1 44 24 23 98
Fax : 00 33 1 44 24 17 96
e-mail:norma.wincker@psl.ap-hop-paris.fr

TABLE OF CONTENTS

Protocol authors and sponsor signatures ……………………………………………………….........2
Investigator Signature Form ……………………………………………………………………........3
Co-Investigator Signature Form ………………………………………………………………........4
Study Personal and Sponsor Representatives ……………………………………………........6
Table of Contents ……………………………………………………………………………….........8
List of Tables ……………………………………………………………………………………........11
List of Appendices ……………………………………………………………………………........12
Abbreviations and Definitions ………………………………………………………………........13
Study Synopsis ……………………………………………………………………………….........14

1. **Introduction** …………………………………………………………………………….........20
   1.1. Background ……………………………………………………………………………........20
   1.2. Rationale for transcutaneous vaccination ……………………………………………........21
      1.2.1. Transcutaneous penetration of compounds …………………………………........21
      1.2.2. Preclinical efficacy of transcutaneous immunization ……………………….........22
      1.2.3. Human preclinical and clinical data experience ……………………………........22
   1.3. Study Rationale ……………………………………………………………………….........23
2. **Study Design** ………………………………………………………………………………........23
3. **Study Objectives** ………………………………………………………………………….........25
   3.1. Primary Objective ……………………………………………………………………….....25
   3.2. Secondary Objectives ………………………………………………………………….......25
4. **Study Population** ………………………………………………………………………….......25
   4.1. Inclusion Criteria ……………………………………………………………………….......26
   4.2. Exclusion Criteria ……………………………………………………………………….......26
   4.3. Number of Patients ……………………………………………………………………........27
5. **Vaccine Immunization** ……………………………………………………………………......27
   5.1. Study vaccine …………………………………………………………………………….....27
   5.2. Study Drug Administration ……………………………………………………………….....28
   5.3. Study Treatment Procedures ……………………………………………………………....28
      5.3.1. Randomization of vaccine mode of administration ……………………………......28
      5.3.2. On site drug accountability and dispensing ……………………………………......29
6. **Concomitant Medication** ………………………………………………………………….......29
   6.1. Prohibited Treatments ……………………………………………………………………......29
   6.2. Present Therapy …………………………………………………………………………......29
7. **Study Plan** ……………………………………………………………………………………..30
   7.1. Screening Period (Day-7) ……………………………………………………………….30
   7.2. Study Entry – Randomization ………………………………………………………….30

7.3. Day 0 – Vaccine Administration ……31
7.4. Evaluation during the treatment period ……32
7.4.1. D1 ……32
7.4.2. D3 and D21 ……32
7.4.3. D7 and D14 ……32
7.4.4. End of Study Treatment Evaluation: D28 ……33
7.5. Premature Study Discontinuation ……35
7.5.1. Premature discontinuation from the study ……35
7.5.2. Subject withdrawal or Discontinuation ……35
7.6. Lost to follow-up ……35
7.7. Subject Replacement ……35
7.8. Technical Procedures ……36
7.8.1. Medical examination ……36
7.8.2. Assessment of the Immune Response ……36
7.8.3. Subjects instructions ……36
7.8.4. Procedure for blood collection and shipment ……37
8. **Study Endpoints** ……37
8.1. Primary Endpoints ……37
8.2. Secondary Endpoints ……37
9. **Statistical Consideration** ……38
9.1. General design issues ……38
9.2. Analysis Plan Overview ……38
9.3. Sample Size Calculations ……38
9.4. Statistical Analysis ……38
9.4.1. Baseline ……38
9.4.2. Primary Endpoints (Safety) ……39
9.4.3. Secondary endpoints (Immunogenicity analysis) ……39
10. **Adverse Events and Serious Adverse Events** ……39
10.1. Adverse Events – Definition ……39
10.1.1. Grading of adverse events ……39
10.1.2. Reporting of adverse events ……39
10.2. Serious Adverse Events - Definition ……40
10.2.1. Grading of serious adverse events ……41
10.2.2. Reporting of serious adverse events ……42
10.2.3. Follow-up of Adverse Event ……42
11. **Data Collection and Study Monitoring** ……43
11.1. Data Collection ……43
11.1.1. CRF ……43
11.1.2. Source documents ……44
11.2. Study Monitoring ……44

12. **Data Management** 45
12.1. Data Entry 45
12.2. Data Review 45
12.3. Data Storage 45
12.4. Data Freezing 45
13. **Ethical Considerations** 46
13.1. Ethical Conditions 46
13.2. Independent Ethics Committee and Health Authorities 46
13.3. Patient Information and Consent 47
13.4. Confidentiality 47
13.5. Insurance Policy 47
13.6. Compensation 47
14. **Administrative Procedures** 47
14.1. Protocol Amendment 47
14.2. Audit 47
14.2.1. Source document requirement 47
14.2.2. Inspection by regulatory agencies 48
14.3. Publications 48
14.4. Clinical Study Report 48
14.5. Source Documents, Investigator's File Storage 48
15. **References** 49

List of Tables

Table 1: Study Flow Chart ………………………………………………………………..19

Table 2: Study design ……………………………………………………………………..24

Table 3: Study procedures at screening period …………………………………………31

Table 4: Study procedures during the study ……………………………………….…34

LIST OF APPENDICES

**Appendix I :**	**Subject Information Sheet and Consent Form**
**Appendix II :**	**Declaration of Helsinki**
**Appendix III :**	**Insurance certificate**
**Appendix IV:**	**Skin physiology measurements**
**Appendix V:**	**Transcutaneous application of Tetagrip® vaccine**
**Appendix VI**	**Blood collection and shipment**
**Appendix VII:**	**Immunological analysis of the immune response against Tetagrip® vaccine**
**Appendix VIII :**	**Randomization Form**
**Appendix IX :**	**Severity of Vaccines reaction**
**Appendix X:**	**SAEs Grading table**
**Appendix XI :**	**SAE declaration Form**
**Appendix XII:**	**Tetagrip® Vaccine Accountability Form**

ABREVIATIONS

AE: Adverse Event

APC: Antigen-Presenting Cell

BMI: Body Mass Index

CRF : Case Report Form

CSSS: Cyanoacrylate Skin Surface Stripping

ELISA: Enzyme-Linked Immunosorbent Assay

ELISPOT: Enzyme-Linked Immunosorbent Spot Assay

GCP : Good Clinical Practice

GMT: Geometric Mean Titers

ICH : International Conference of Harmonisation

IEC : Independent Ethics Committee

I.M: Intramuscular

SAE : Serious Adverse Event

SOP: Standard Operating Procedure

TEWL: Trans-Epidermal Water Loss

SCH: Stratum Corneum Hydration

STUDY SYNOPSIS

**NAME OF SPONSOR:**	**ORVACS**<br>Hôpital Pitié-Salpêtrière, Pavillon Laveran<br>47-83 bd de l'hôpital, 75651 paris 13, cedex
**Name of the new tested vaccine administration mode**	**TRANSCUTANEOUS VACCINATION**
**Name of the investigational product**	**Tetagrip® vaccine**
**Study code**	**MANON 05 - CUTAVAC**
**Title of the study**	A randomized comparative Phase I study to evaluate and to compare the safety and immunogenicity of a transcutaneous mode of administration of a licensed Tetanus / Influenza vaccine to the conventional intramuscular route of vaccine administration in healthy volunteers and HIV-infected patients.
**Protocol Chairman**<br>**Protocol Co-Chairman**<br>**Cohort I**<br>**Clinical Study Center I**<br>**Principal Clinical Investigator**<br>**Co-Investigators**<br>**Cohort II**<br>**Clinical Study Center II**<br>**Principal Clinical Investigator**	**Pr.Christine KATLAMA, MD,**<br>**Pr. Brigitte AUTRAN, MD PhD**<br>Charité – Universitätsmedizin Berlin, Department of Dermatology and AllergyAllergy, Clinical Research Center for Hair<br>and Skin Physiology, Germany<br>**Pr. Ulrike BLUME-PEYTAVI, MD PhD**<br>Dr. Annika VOGT, MD<br>Dr. Gregor SCHÄFER, MD<br>Hôpital Pitié Salpêtrière<br>Service des Maladies Infectieuses<br>Pavillon Laveran<br>47-83 Bd de l'hôpital<br>75651 Paris Cedex 13<br>**Pr.Christine KATLAMA, MD,**

**Clinical Study Center III**	Hospital of the Johann Wolfgang Goethe-University<br>Frankfurt am Main<br>HIV Treatment & Research Unit<br>Department of Internal Medicine II<br>Theodor-Stern-Kai 7<br>60590 Frankfurt am Main -Germany
**Clinical Investigator**	**Pr Schlomo STASZEWSKI, MD**
**Co-Investigators**	Dr. Carsten ROTTMANN, MD<br>Dr. Nari OH, MD
**Immunology Study Center**	**Laboratoire d'Immunologie Cellulaire et Tissulaire**<br>Université Pierre et Marie Curie<br>Hôpital Pitié Salpêtrière,<br>83 Bd de l'Hôpital,<br>75013 Paris - FRANCE
**Immunology Investigator**	**Brigitte AUTRAN, MD, PhD**
**Immunology Co-Investigators**	Béhazine COMBADIERE, PhD<br>Brice MAHE, MS
**Methodology and Data Management**	**Dominique COSTAGLIOLA, PhD**
**Project leader and Study Manager**	**Norma WINCKER, PhD**
**Clinical Phase**	Phase I
**Methodology**	Phase I, open label, randomized, multicenter, two cohorts, two-arm study comparing a transcutaneous mode of administration to a intramuscular administration of the Tetagrip® vaccine in healthy voluunteers (Cohort I) and in HIV-infected patients (Cohort II)
**Investigational mode of administration, investigational product, and dose**	Tetagrip® vaccine will be administered on two skin areas of 4x4 cm each (32 cm^2) on the external part of the upper left arm transcutaneously and will be compared to a conventional intramuscular injection.<br>The selected dose is 0.5 ml that corresponds to one vaccine dose of tetanus toxin and 15 μg of two type-A (H3N3 and H1N1) and one type-B virus subunits (This information will be updated as soon as they will become available).
**Number of subjects**	48 in two cohorts of :<br>Cohort I: 24 Healthy volunteers and<br>Cohort II: 24 HIV chronically infected patients under efficient antiretroviral treatment with baseline CD4+ counts $\geq$ 350 cells/mm3 since at least 1 year and a CD4 nadir >200/mm3

**Study period**	5 weeks per participant
**Study duration**	The duration of the study will be approximately 3 months<br>September 2005 / December 2005
**Randomization**	In each of the two cohorts subjects will be randomized on the mode of Tetagrip® vaccine administration to one of the two arms. Subjects fulfilling the inclusion criteria will be randomly assigned in equal numbers (12 per arm) to one of the two arms as described below :<br><br>ARM / Administration mode / Volume / Number of subjects<br>A / Transcutaneous / 0.5 ml / 12<br>B / Intramuscular / 0.5 ml / 12<br><br>Randomization will be performed using the SAS procedure by the Data Management and Statistics Center.
**Vaccine schedule**	Each subject will receive one single administration 0.5 ml of Tetagrip® vaccine either transcutaneously on two sites of 4x4 cm skin area (total area of 32 cm^2) on the deltoid region or via conventional intramuscular injection.
**Objectives**	***Primary Objective:***<br>• To evaluate the safety of a transcutaneous mode of tetanus/influenza vaccine administration of Tetagrip® vaccine in healthy volunteers and in HIV infected patients.<br>***Secondary objectives:***<br>To compare in each of the two cohorts, the two arms between baseline and day 7 (D7), day 14 (D14) and day 28 (D28) post vaccination:<br>• Specific antibodies: Geometric titers (GMT) with 95% confidence interval,<br>• The increase in tetanus and influenza specific antibody titers,<br>• The pre/post vaccination ratios of specific antibodies titers,<br>• The increase in tetanus and influenza specific T cells (CD4 and CD8),<br>• To characterize -specific effector and memory tetanus and influenza T cells,<br>• Proportion of participants who responded (50% increase in titers)

**Inclusion Criteria**	• **Cohort I**<br>- Healthy male volunteers<br>- Negative HIV test within the last 3 months<br>- Clinical examination and an interview on their medical history and possible current therapies<br>• **Cohort II**<br>- HIV infected males<br>- Positive HIV-serology<br>- Efficient antiretroviral treatment, with baseline $CD4^+$ counts $\geq$ 350 cells/mm^3 since at least 1 year and a CD4 nadir >200/mm^3<br>- Clinical examination and an interview on their medical history and possible current therapies<br><br>For both cohorts the same following inclusion criteria will be meet :<br><br>• Age between 18 and 45 years<br>• BMI between 21 - 26<br>• Phototype I-IV<br>• Absence of *tetanus* vaccination within the last 4 years<br>• Absence of *influenza* vaccination in the past year<br>• Absence of any psychological, familiar, sociological or geographical condition potentially hampering compliance with the study protocol schedule.<br>• Written informed consent

**Main Exclusion Criteria**	• Excessive terminal hair growth on the investigational skin area<br>• Phototype V-VI<br>• Any skin affection which may interfere with the trial assessment on the injection or application site<br>• Any allergy or hypersensibility to one of the components of the Investigational Product (e.g. egg products, neomycin)<br>• Medical history of allergy or hypersensitization to any ingredient of colorant used in the transcutaneous mode of vaccination.<br>• Medical history of skin cancer<br>• Acute illness, e.g. fever, infection at Screening and/or D_0<br>• Any acute or chronic illness which may expose the subject or interfere with results of the trial<br>• Use of any topical treatment on the injection or application site within the last four weeks<br>• Continuous topical and systemic treatment that would interfere with assessment and/or investigational treatment (e.g. Immunosuppressors or any Immunomodulator agent)<br>• Prevision of UV sessions or sun exposure 4 weeks prior to the study or during the study period<br>• Subject being in the exclusion period of a previous clinical trial (1 month),
**Study endpoints**<br>**Primary endpoint:**<br>**Safety assessment**<br><br><br><br>**Secondary endpoints:**<br>**Immunogenicity assessments**	Safety will be assessed at each visit by recording clinical local and general tolerance :<br>• Clinical examination,<br>• Local lymph nodes examination,<br>• Questioning of the patients immediately after injection or application and afterwards at each visit,<br>• Patient diary card observations,<br>• Reporting of any adverse events according to the grading table in appendix .<br><br>Immunogenicity will be evaluated as the specific immune response including detection of:<br>• Tetanus and influenza specific antibody titers<br>• Tetanus and influenza specific T cells (CD4 and CD8)<br>• Effectors and memory T cells differentiation
**Statistical methods and Analysis**	Safety will be assessed during all the study by recording any Adverse Event (AE) graded ≥ 3 according to toxicity criteria (Appendices IX, and X) .<br>Immunogenicity will be assessed for each subject at D7, D14 and D28. Groups will be compared by ITT and per protocol analysis using non parametric tests.

MANON 05

Table 1: Study Flow Chart

Timing	*Screening Period*	*Treatment Period (Vaccine administration + follow-up)*						*End of Study treatment evaluation*
	D-7	*D0*	*D1*	*D3*	*D7*	*D14*	*D21*	*D28*
Required Assessments								
Written Informed Consent	x							
Medical History / Demography /	x							
Concomitant medication	x							
Physical examination	x							
Weight	x							
BMI	x							
Vital Signs	x							
Blood withdrawal for Immunology assays		x			x	x		x
Skin physiological measurements		x						
Randomization		x						
Administration of Tetagrip® vaccine		x						
Cohorte II: Monitoring of HIV HIV-1 PCR, CD4		**x**						**x**
Safety								
Local and general tolerance		x	x	x	x	x	x	x
Diary Card to patient		x						
Removal of patch in Arm A			x					
Diary patient data reporting onto the CRF			x	x	x	x	x	x
Immunogenicity								
Tetanus and Influenza antibodies		x			x	x		x
T cells assays		x			x	x		x

1. INTRODUCTION

1.1. Background

Classical prophylactic vaccines aim at preventing infection by inducing in naive individuals a strong immune memory that will control pathogen dissemination at entry in the organism. Another concept is to use vaccines as therapeutic tools during an established infection by re-inforcing or broadening defenses when specific immune responses are unable to do so during the natural course of the disease and when a conventional antimicrobial therapy is not available or efficacious enough (Autran 2003). This strategy is currently being evaluated in HIV infection where therapeutic vaccines aim at limiting costs and toxicity of a lifelong antiretroviral therapy and at preventing disease progression in the absence of treatment by re-inforcing immunity to HIV.

However, although conventional vaccines have generated major successes in the control of infectious diseases, several obstacles remain in the development of vaccines against pandemic chronic diseases such as HIV against which the current candidate vaccines are still too weak to ensure protection (McMichael 2003), or in the access to vaccines in developing countries which require safe, well tolerated and easy to use vaccines. In addition when HIV-infected patients initiate antiviral therapy at advanced stages of the HIV disease, immune reconstitution, though potent at restoring most immune defenses agianst pathogens (Autran 1997) might not be strong enough to ensure optimal responses to conventionnal vaccines (Lange 2003).Therefore progresses have to be made to improve the efficacy of these still relatively weak preventative vaccines in the development phase and to overcome the multiple obstacles opposed to the development of therapeutic immunization against HIV or of preventative vaccines in immune suppressed individuals.

Vaccines are efficient at priming strong and protective immune responses to pathogens by targeting and activating dendritic cells (DCs) which play a unique role as primers of antigen-specific immune responses. Vaccines are classically injected into muscles or subcutaneous tissues where local depots of vaccine compounds have to be captured by immature DC and promote DC maturation and migration to the draining lymph nodes where they prime the naive vaccine-specific T cells. Those tissues are however very poor in DC and vaccines generally require adjuvants to augment DC recruitment and activation and to potentiate their immunogenicity. In addition, part of the vaccine is bound unspecifically to connective tissue or undergoes degradation. Furthermore, conventional i.m. or s.c. injections raise some psychological or cultural difficulties (fear of needle), injection procedure with the risk of injury and blood transmission and, exhibit further practical disadvantages and hazards including infection caused by unsterile equipment or unsterile reconstitution, instability of the vaccine preparation and injury due to improper injection techniques (Jodar 2001).

A future goal for vaccine design is therefore to increase their efficiency by reaching the highest number of antigen-presenting cells (APCs) possible and to achieve the high local concentrations required to induce a potent immune response, as well as to use non-invasive

modes of administration. A more efficient targetting of vaccines toward DC would help at enhancing their immunogenicity among which the transcutaneous route of administration appears to be the most promising.

1.2. Rationale for transcutaneous vaccination

Transcutaneous vaccine delivery offers an advantageous mode of immunization due to the unique ability of cutaneous immune cells, especially Langerhans cells, to present antigens to the immune system. Langerhans cells, upon activation, migrate to the regional lymph nodes and lead to the generation of systemic and mucosal immune responses. Indeed, the rationale for transcutaneous vaccination is strong: DCs can be found at high densities in the epidermis and the dermis of human skin, a fraction of which are the epidermis Langerhans cells (LC). It is known that strong and efficient immune responses can be induced by targeting vaccines to skin APCs (Glenn 2003, Partidos 2003), e.g. by epicutaneous application of smallpox vaccine on scarified skin.

1.2.1. Transcutaneous penetration of compounds

Thus dermal or transdermal delivery of vaccines has become a great challenge. Several obstacles however prevent vaccines from attaining sufficiently high and free concentrations in these target skin DC. In intact skin, the percutaneous absorption of epicutaneously applied compounds is first limited by the stratum corneum which forms a highly structured and tight barrier on the skin surface, very effective in preventing entrance of compounds into the skin. Under physiological conditions, active compounds in solutions or ointments spread rather homogenously on the skin surface which leads to small local concentrations of the topically applied active compound and augments the unspecific binding of active compounds to skin proteins, which further reduces their free concentration. The passage through the horny layer, , occurs via a very slow diffusion and larger molecules including proteins hardly overcome this barrier at all. Secondly, the upper living layer of the skin, the epidermis, maintains continuous renewal and sheds cells off, thereby rejecting bound active compounds to the skin surface. Thirdly, a dense microvasculature system below the epidermis rapidly and removes active compounds from the skin to the systemic compartment.

Recent investigations by our own group and others revealed that the penetration of topically applied compounds is considerably lower in hairless skin as compared to haired skin, suggesting that hair follicles are important entry pathways for epicutaneously applied compounds skin (Hueber 1994, Tenjarla 1999, Schaefer 2001). Under physiological conditions, however, only one third of the hair follicles in glaborous skin are open to penetration. The rest can be opened by cyanoacrylate skin surface stripping (CSSS), which removes debris from the follicular openings (“follicular biopsy”) (Lademann 2001) and hereby improves the percutaneous penetration of epicutaneously applied active compounds.

Cutaneous DC are in fact particularly concentrated in the infrainfundibulum of the hair follicles where they should be a target of choice for vaccines (Taira 2002, Christoph 2000).

1.2.2. Preclinical efficacy of transcutaneous immunization

Transcutaneous immunization can be achieved in animal models by using different procedures described below and could provide some protection against influenza virus. Recent studies further revealed that vaccination works through intact skin. Robust and specific immune responses to hepatitis B surface antigen could be induced by topical application of naked plasmid DNA in aqueous solution on untreated skin of mice (Fan 1999).

Likewise, other group could recently induce strong immune responses to HIV in naive rhesus macaques who were immunized through a dermal application of a naked DNA plasmid vaccine encoding for HIV. After the topical application onto a large skin surface ($40cm^2$) , transduced DC were detected in the draining lymph nodes. This topical ex vivo DC-based vaccination induced SIV-specific CD4 helper and CD8 memory T cells comparable to the immune responses obtained via the i.m. route in control animals (Lisziewicz 2005).

We have developed a successful new technique for specific vaccine delivery to skin professional antigen-presenting cells (LC and dermal DC) in murine models. Indeed, we have shown that when proteins are delivered through the transcutaneous mode of administration, they directly target dermal dendritic cells that activate and migrate to the proximal lymph node. This method allows the concentration of vaccinal compound into the professional antigen-presenting cells. The arrival of such APC loaded with vaccinal antigens then initiates the immune response by activating T and B cells. Furthermore vaccine delivery through transcutenous administration triggers specific cell proliferation and activation, cytotoxic and helper functions as well as humoral immune responses in C57/B6 mice. These results have been obtained using protein, DNA or conventional vaccine (i.e TETAGRIP®) administration. Immune responses induced in a mice model of transcutaneous immunization were protective when mice were challenged with vaccine antigen-expressing tumor cell line (B Combadiere & B Mahe, manuscript in preparation).

1.2.3. Human preclinical and clinical data experience

In a first attempt to immunize humans via transcutaneous vaccination, Glenn et al. induced robust immune responses against heat-labile enterotoxin from E. coli in human volunteers using a patch system (Glenn 2000).

Kenney et al. have recently shown that intradermal multipuncture administration of only one fifth of the standard i.m. dose of an influenza vaccine elicited an immunogenicity similar or better than that elicited by common i.m. injection (Kenney 2004). This procedure remains however invasive and hazardous. The classical approach of injecting vaccines intradermally or subcutaneously in saline, buffer etc., though bypassing the cutaneous barrier, does not fulfil any of the criteria mentioned above in a satisfactory manner. In contrast to previous vaccination studies by other groups, the application protocol proposed in this study includes skin preparation with CSSS to obtain a maximal penetration of the vaccine preparation.

Cyanacrylate Skin Surface Stripping (CSSS) removes keratinized material, lipids and other cell debris from the follicular openings and approx. 30% of the stratum corneum. Occasionally hair

fibres, especially vellus hairs, are removed as well (Mills 1983). The remaining stratum corneum and the viable epidermis are left intact. This technique is routinely used in our laboratory and also by other groups to improve the percutaneous penetration of topically applied compounds and to determine the amount of substance which enters the follicular reservoir (Hueber 1994, Schaefer 2001).

1.3. Study rationale

The proposed study aims to translate our current knowledge about vaccinology, immunology of the skin and on transcutaneous penetration of epicutaneously applied active compounds, into the development of more efficient and well tolerated vaccines, and to progress toward an easy-to-apply patch system for transcutaneous application of vaccines.

To that purpose we aim at testing the safety and immunogenicity of a new transcutaneous route of vaccine administration. We propose to test this new route first with a well-known, safe and highly immunogenic vaccine i.e. anti-influenza and tetanus vaccine which is licensed for sub-cutaneous and intra-muscular routes, and to compare the vaccine-specific immune responses induced after transcutaneous administration to the conventional intramuscular (i.m) injection. Seasonal vaccination against influenza is recommended for all individuals at risk for severe flu, including persons above 60 years of age or suffering from chronic dieases and for medical personal. It is also highly recommended at any age to prevent influenza. In addition vaccination against tetanus is mandatory in childhood and requires recall injections every 5 years to protect against the lethal disease caused by the tetanus toxin.

We hypothesize that the transcutaneous application of a licensed anti-influenza-tetanus vaccine in the commercially available standard preparation of 0.5 ml should be capable to induce at least similar antibody and CD4 and/or CD8 T cell responses to both the tetanus and the flu vaccinal antigens.

Tetagrip® vaccine is an approved and commercially available vaccine manufactured by Sanofi-Pasteur, administered in one injection for preventive vaccination of adults against tetanus and influenza. The Tetagrip® vaccine therefore represents a safe and approved test vaccine to evaluate safety and immunogenicity of the mode of administration under investigation.

The long term goal of this strategy is to improve the efficacy of vaccines that are currenly encountering major obstacles such as the HIV vaccines, and to develop a non invasive mode of vaccine administration. Results from this study will help establish a standardized study protocol for the application of HIV-vaccines in future clinical trials.

2. STUDY DESIGN

This Phase I, open label, randomized study is designed to evaluate and to compare the safety and immunogenicity of a transcutaneous mode of Tetanus / Influenza vaccination to the conventional i.m. route of vaccine administration in two cohorts: The cohort I constituted of healthy volunteers and the cohort II of HIV-infected patients in whom the virus is stably

controlled by antiretroviral therapy, ensuring an immune competence and a capacity to respond to vaccines.

This trial will be conducted in three clinical centers:

Cohort I:

Charité – Universitätsmedizin Berlin
Clinical Research Center for Hair and Skin Physiology
Department of Dermatology and Allergy
Schumannstr. 20/21
10117 Berlin, Germany.

Cohort II :

Hôpital Pitié Salpêtrière
Service des Maladies Infectieuses
Pavillon Laveran
47-83 Bd de l'hôpital
75651 Paris Cedex 13 – France

Hospital of the Johann Wolfgang Goethe-University Frankfurt am Main
HIV Treatment & Research Unit
Department of Internal Medicine II
Theodor-Stern-Kai 7
60590 Frankfurt am Main -Germany

Twenty-four subjects will be accrued in each cohort. Cohort I's 24 individuals will be recruited in the Berlin center. Patients from Cohort II will be accrued in Paris and Frankfurt centers (12 in each center).

Participants will be enrolled concurrently for both arms, and will randomly assigned to one of the two arms and will receive a single dose of 0.5 ml Tetagrip® vaccine either transcutaneously (Arm A) or via i.m. injection (Arm B) at D0 (see table 1).

Table 2: Study design

ARM	Administration mode	Volume	Number of subjects
A	Transcutaneous	0.5 ml	12
B	Intramuscular	0.5 ml	12

Patient accrual (time between first and last inclusion) shall be completed within 4 weeks from the beginning of the study planned in September 2005 until December 2005.

Study duration per patient is 5 weeks (1 week for screening plus 4 weeks of follow-up) including immunogenicity evaluation.
The clinical trial will be completed by the end of 2005. Therefore, non-responders will have the possibility to receive conventional i.m. injections before the influenza season 2005/2006 .

3. STUDY OBJECTIVES

3.1. Primary objective :

To evaluate the safety of a transcutaneous mode of a tetanus/influenza vaccine (Tetagrip®) administration in healthy volunteers (Cohort I) and in treated HIV-infected patients(Cohort II).

Safety will be assessed by recording adverse events (including cutaneous reactions) at each post-inclusion visit.

3.2. Secondary objectives:

To evaluate in the two arms of the two Cohorts between baseline and day 7 (D7), day 14 (D14) and day 28 (D28) post vaccination:

- Protective tetanus and influenza specific antibodies Geometric titters (GMT) with 95% confidence interval,
- The increase in tetanus and influenza specific antibody titters,
- The pre/post vaccination ratios of specific antibodies titters,
- The increase in tetanus and influenza specific peripheral blood T cells (CD4 and CD8) numbers,
- The vaccine -specific effectors and memory tetanus and influenza peripheral blood T cells,
- Proportion of participants who responded (50% increase in titters)

4. STUDY POPULATION

4.1. Inclusion criteria

Cohort I

- Healthy male volunteers
- Negative HIV test within the last 3 months

Cohort II

- HIV infected males
- Positive HIV-serology
- $CD4^+$ counts $\geq$ 350 cells/mm^3 over the last year
- CD4 cells nadir >200/mm^3

- Plasma HIV RNA < 400cp/ml over the last 6 months
- Efficient antiretroviral treatment with a minimum of three drugs since at least one year

In addition Patients from both cohorts must meet the following criteria to be eligible for the study:

- Age between 18 and 45 years,
- BMI between 21 – 26,
- Phototype I to IV
- Clinical examination and an interview on their medical history and possible current therapies
- Subjects able to receive vaccine administration by any of the two administration routes,
- Absence of tetanus vaccination within last 4 years,
- Absence of influenza vaccination in the last year,
- Absence of any psychological, familiar, sociological or geographical condition potentially hampering compliance with the study protocol schedule.
- Written informed consent

4.2. Exclusion criteria

In both cohorts, if any of the following apply, the subject cannot enter the study:

- Excessive terminal hair growth on the two investigational skin areas used for the transcutaneous mode of vaccination.
- Phototype V-VI
- Acute illness, e.g. fever, infection at screening and/or D_0
- Any acute skin affection which may interfere with the trial assessment on the injection site,
- Any allergy or hypersensibility to one of the components of the Investigational Product (egg products, neomycin),
- Medical history of allergy or hypersensitization to any ingredient of colorant used in the transcutaneous mode of administration,
- Medical history of skin cancer,
- Any acute or chronic illness which may expose the subject or interfere with results of the trial,
- Use of any topical treatment on the injection site within the last four weeks,
- Use, within the past 3 months, of any topical and systemic treatment that would interfere with assessment and/or investigational treatment (anti-inflammatory drugs, immunosuppressors or any immune modulator agent),
- Prevision of UV sessions or sun exposure 6 weeks prior to the study or during the study period,
- Subjects with current alcohol or illicit drug use which, in the opinion of the investigator, may interfere with the subject’s ability to comply with the dosing schedule and protocol evaluations.
- Subject being in the exclusion period of a previous clinical trial (1 month.)

In addition in each cohort:

Cohort I:

- HIV infection

Cohort II:

- Any acute infectious event within 30 days prior to enrolment
- Subjects who have participated in an investigational HIV vaccine trial or any immunomodulatory trial in the last 6 months
- Subjects suffering from serious medical conditions, which, in the opinion of the investigator, would compromise the safety of the subject.
- Patients having received IL2 or any other immunomodulator drug in the last 6 months

4.3. Number of subjects

A total of 48 individuals will be recruited.
24 subjects (12 per study arm) in each cohort.

Cohort I:
24 healthy volunteers (HIV negative)

- 12 subjects in Transcutaneous Arm (Arm A)
- 12 subjects in Intramuscular Arm (Arm B)

Cohort II:
24 HIV infected patients

- 12 subjects in Transcutaneous Arm (Arm A)
- 12 subjects in Intramuscular Arm (Arm B)

5. VACCINE IMMUNIZATION

5.1. Study vaccine

- The immunization will use Tetagrip® a commercially available vaccine, at the recommended dosage.
- The selected vaccine Influenza/Tetanus vaccine is registered as Tetagrip® trademark by Sanofi-Pasteur MSD, France.
  - Presentation: Injectable suspension presented as 0.5 ml refilled syringe.
  - Composition: Each 0.5 ml contains: One vaccine dose of tetanus anatoxin plus inactivated influenza virus type A and type B fragments analogous to the influenza antigens equivalent to 15 µg of 2 type-A (H3N3 and H1N1) and 1 type-B virus hemagglutinine subunits in saline solution. (This information will be updated as soon as they will become available for the flu season 2005/2006).

The composition of the product will not be modified for study purposes.

The influenza virus was grown on chicken egg for vaccine subunits production. Other components of the vaccine are sodium chloride, sodium phosphate, potassium chloride, potassium phosphate and, injectable water.

- Shelf-life and storage conditions: The expiry date is indicated on labels on the box. The vaccine should be stored under refrigeration (2 to 8 °C), protected from light and must not be used if coloration is observed.
- Commercial Tetagrip® vaccine will be supplied by ORVACS to the hospital pharmacist. The specific form "Drug Accountability Form" will be used in order to collect information regarding Tetagrip® vaccine itself (quantity, batches and expiry dates).

5.2. Study drug administration

Tetagrip® vaccine, 0.5 ml will be administered transcutaneously on two skin areas of 4x4 cm on the deltoid muscle and will be compared to a conventional intramuscular injection.

- ***Transcutaneous mode of vaccine administration (Arm A)***

Tetagrip® vaccine will be applied as described in the *Standard Operating Procedure*, see Appendix VIII. Briefly, the two investigational skin areas of 4x4 cm each will be delimitated on the external part of the upper left arm. After shaving of the skin, cyanacrylate skin surface stripping (CSSS) will be performed. 0.25ml Tetagrip® vaccine will be applied on each investigational skin area. A silicone barrier will limit the spreading of the vaccine to the investigational skin area. At the end of the procedure, a hydrocolloid bandage will be applied to the investigational skin areas for the following 24 hrs.

- ***Intramuscular injection (Arm B)***

Volunteers who receive Tetagrip® vaccine via intramuscular injection will receive the vaccine (0.5 ml) into the deltoid muscle according to Good Clinical Practice after careful disinfection.

5.3. Study treatment procedures

5.3.1. Randomization of vaccine mode of administration

After verification of the eligibility criteria by the statistical department, healthy volunteers (Cohort I) or HIV-infected patients (Cohort II) will be randomly assigned to one of the two administration routes. Randomization will be based on SAS procedure plan. The randomization will be performed by the Data Management and Statistics Center. The procedure is described in **section 7.2.**
After validation of the inclusion criteria, subjects will be randomized on the administration mode of Tetagrip® vaccine to one of the two study arms:

Arm A: Transcutaneous mode of vaccination
Arm B: Intramuscular injection

5.3.2. On site drug accountability and dispensing

The hospital pharmacist (Center II) or the investigator (Center I, Center III), are responsible for adequate storage of the study medication according to the manufacturer recommendations and for dispensing the treatment to the study participants.

Tetagrip® vaccine must be used in accordance to the protocol and only by the investigator.

The investigator and/or the pharmacist must maintain adequate and accurate records including batch number of Tetagrip® vaccine. For this purpose, the Drug Accountability Form should be used. This includes the patient's identification, the date of dispensing, each quantity dispensed, and the identification of the dispenser. The original will be kept by the Sponsor and the copies will be left: one in the pharmacist's file and one in the investigator's file.

6. CONCOMITANT MEDICATION

All treatments given in addition to the study vaccine at subject's study entry (D-7) and/or during the study period are regarded as concomitant treatments and will be documented on the appropriate pages in the CRF.

6.1. Prohibited treatments

The following medications are not allowed during the entire trial period:

- Any topical treatment of the investigational site within the past 4 weeks,
- Systemic treatment (drug or cosmetic), which may interfere with the trial assessment (anti-inflammatory drugs, immunosuppressors or any immune modulator agent)

6.2. Present therapies

All the concomitants therapies needed by the subjects will be recorded in the CRF throughout the study.

Patients of cohort II will continue their antiretroviral treatment for the entire duration of the study. Names, dosage and dosing intervals of the antiretroviral drugs will be recorded in the CRF throughout the study. Furthermore, any change in their antiretroviral treatment need to be recorded in the patient's CRF.

7. STUDY PLAN

Subjects will be informed about the study, both verbally and by reviewing the patient information sheet and consent form at day of screening. The subject must be given the opportunity to ask questions and given time to consider his participation. The investigator and the subject will both sign and personally date the consent form as confirmation of consent.

7.1. Screening Period (Day -7)

The screening time is the interval preceding registration, and includes 7 day period for performing screen assessments.

Written informed consent will be obtained from the volunteer before any study specific procedure is undertaken.

Once written consent has been obtained, subjects will undergo a baseline assessment:

- Verification of all inclusion/non inclusion criteria
- Interview of subjects on their medical history and vaccinations
- General medical examination
- Concomitant medications
- Identification code

Afterward, the investigator can ask for subject randomization by faxing the Registration form (Appendix VIII) to the Methodology/Data management:

Tchadie Bommenel
INSERM U720
Fax number: (33) 1 42 16 42 61 / 42 67

7.2. Study entry – Randomization

All inclusion/exclusion criteria will be checked during the randomization procedure. The study monitor or study manager will notify the investigator by fax within two working days. In case of controversy on patient eligibility, or absence of any required assessment (see table 2), the investigator shall be contacted for discussion. In any case test results and assessments required to establish eligibility must be obtained prior to the administration of the vaccine.
Randomization of the subject to one of the two study arms (if all inclusion criteria are filled) will be performed by the Methodology/Data management department. .
The Methodology/Data management department will assign a registration number to the patient and allocate the administration mode of Tetagrip® vaccine. Each patient will receive a 4 digit number followed by subject initials. The first two digits represent the center and the last two digits represent the subject within the center. This subject code must be reported on all CRF pages and in any study document.

Randomization represents the starting point of the study. It is recommended to perform vaccination within no more than **one week**. In any case all events occurring after the registration must be recorded onto the CRF and will be taken into account in the analysis, whether the patient received the treatment or not.

Table 3: Study procedures at screening period

Study procedure	Timing - Comments
Written informed consent	**To be obtained and signed both by the subject and the investigator prior to any study specific procedure**
Demography, Medical History,<br><br>Vaccination procedure:	To be done once at baseline<br><br>To be done once at D0
**Complete physical examination**<br>Height, weight, blood pressure and pulse, temperature,	Within 7 days prior to Tetagrip® vaccine administration
Concomitant treatments	To be recorded in the CRF from D -7 prior to Tetagrip® vaccine administration and during all the study period
Confirm eligibility according to the Inclusion/Exclusion Criteria	**Before randomization**
**Patient randomization: Mandatory before starting study drug administration in any arm**	

7.3. Day 0 - Vaccine administration

Subjects will be definitely included in the study at D0.

Procedures corresponding to this visit are:

- Blood withdrawal for immunological determinations to be done in Paris immunology laboratory
- Tetagrip® vaccine administration according randomization:

<u>ARM A: TRANSCUTANEOUS ADMINISTRATION</u> (see Appendix V for details)

- Identification of the injection site under investigation site: 4x4 cm on the deltoid region.
- Shaving of the injection site
- Delimitation of the investigational site by a permanent skin marker
- Assessment of skin physiological paramerters (pH, TEWL, SCH, sebum production, see Appendix VII for details)
- Transcutaneous application of 0.5 ml Tetagrip® vaccine **.** The transcutaneous application of Tetagrip® vaccine will be done by the same study nurse to maximize the standardization and the reproducibility of the procedure.
- At the end of the procedure a hydrocolloid bandage will be applied for 24 hours

<u>ARM B: INTRAMUSCULAR</u>

- Desinfection of the injection site
- Intramuscular injection of 0.5 ml Tetagrip® vaccine in the deltoid muscle

7.4. Evaluation during the treatment period

The study period begins with randomization and continues until 30 days after study treatment administration.

The same methods for assessment used at baseline must be used throughout the study to ensure comparability.
All adverse events which occurred between 2 visits must be assessed and documented using the toxicity guide. Subjects should be asked for recording any sign and symptom onto the patient diary card
The following assessments need to be completed throughout the treatment period:

7.4.1. D1

- Removal of the patch *(to be specified after our preliminary investigations)*
- Medical examination of the injection site, examination of the local (axillary) lymph nodes
- Review of symptoms and toxicities: Adverse Events / SAEs
- Concomitant medication,

7.4.2. D3 and D21

- Medical examination of the investigational site, examination of the regional (axillary) lymph nodes,
- Review of symptoms and toxicities: Adverse Events / SAEs,
- Concomitant medications

7.4.3. D7 and D14

- Medical examination of the investigational site, examination of the regional (axillary) lymph nodes,
- Review of symptoms and toxicities: Adverse Events / SAEs,
- Concomitant medications,
- Blood withdrawal for immunology determinations.

7.4.4. End of study treatment evaluation: D28

A clinical work-up will be performed at D28 after vaccine administration in both arms. It will include the following assessments (see also table 3):

- Physical examination: weight, vital signs (blood pressure, pulse, temperature)
- Medical examination of the investigational site, examination of the local (axillary) lymph nodes
- Review of symptoms and toxicities: Adverse Events (AE) / Serious AEs,
- Concomitant medication
- Blood withdrawal for immunological determinations.

Table 4: Study procedures during the study

Assessment	Timing - Comments
Identification of the injection or application site, delimitation of the two investigational skin areas by permanent skin marker	To be done only at D0
Assessment of skin physiological parameters:<br><br>pH, TEWL, SCH, sebum production	To be done at once at baseline.<br><br>See Appendix VII for details
Vaccine administration	To be performed at D1
Concomitant treatments	To be recorded from D-7 to the end of the study
**Safety**	
Local tolerance: Examination of the Injection site and axillary lymph nodes	To be performed immediately after vaccine administration and throughout the study at each visit (D1, D3, D7, D14, D21, D28)
General tolerance: Review of symptoms and toxicities: Adverse events/SAE<br>The severity of local and systemic reactions to vaccine and/or the administration route is graded according to the Toxicities table (Appendix IX)	To be performed immediately after vaccine administration and throughout the study at each visit (D1, D3, D7, D14, D21, D28)
Complete physical examination<br>Height, weight, blood pressure and pulse, temperature	To be done only at the end of the treatment period (D28)
**Immunogenicity**	
Immunogenicity Determinations*<br><br>Tetanus/Influenza specific antibodies + Tetatus/Influenza specific T cells (CD4/CD8)	To be done at Screening, D0, D7, D14 and D28
**Other study procedures**	
Specimen collection shipment<br>Cryopreservation and storage of specimens	To be done at D0, D7, D14 and D28

* Total blood volume drawn from each subject will be 160 ml (40ml per sample)

7.5. Premature study discontinuation

7.5.1. Premature discontinuation from the study

A subject may voluntarily discontinue his participation in this study at any time. The investigator may also, at his/her discretion; discontinue the subject from participation in this study at any time. If a subject is prematurely discontinued for any reason, the investigator must perform the assessments outlined in section 8.5 (End of study treatment evaluation).

These data should be recorded, as they comprise an essential evaluation that should be done prior to discharge of any subject from the study. When the subject is discontinued from the study due to an AE or SAE (as defined in section 10) the procedures stated in section 9 must be followed.

7.5.2. Subject withdrawal or Discontinuation

Subjects will be withdrawn from the trial by the investigator at any time for any of the following administrative or medical reasons:

- For safety reasons: when the investigator feels that it would be detrimental for the subjects to continue the trial, for example, in case of the occurrence of a Serious Adverse Event/toxicity directly related to the investigational route and/or investigational product(s).
- For patient non-compliance reasons: poor compliance with the requirements of the study protocol (e.g. avoidance of sun exposure during the study period).
- For scientific reasons: in case of any event which is known to affect the efficacy criteria: in case, for example, of systemic disease onset (without any relation to the investigational products but occurring during the trial) for which a therapy by corticoids would be requested.
- Investigator non-compliance: Any significant deviation from the protocol without prior agreement of the Sponsor.
- Any other reason to be documented

  The primary reason for withdrawal will be clearly documented in the subject's medical records and in the CRF. A final evaluation will be completed at the time of discontinuation from the study.

7.6. Lost to follow-up

If a subject misses one visit, the investigator will to try at least twice to contact him. Without any success, a written letter will be sent to the subject. Without any response from the subject, he will be considered lost to follow-up. All of these actions will be documented in the subject's medical file as well as in the Case Report Form.

7.7. Subject replacement

To insure the availability of 10 evaluable cases per study arm we plan the inclusion of 12 cases per study arm to replace discontinuing or withdrawn subjects.

7.8. Technical procedures

7.8.1. Medical examination

The medical examination prior to the inclusion, will include a medical history interview and a clinical examination, to make sure of the eligibility of the subject .A medical examination will also be performed on study termination date.
These examinations will be performed by the investigator.

- ***Local clinical examination***

The local clinical examination includes a careful inspection of the investigational site for local tolerance (see 7.8.4 for details) and an examination of the local (axilllary) lymph nodes.
All the clinical assessments to be performed in one subject will always have to be done by the same physician, except in case of absolute necessity. This should then be documented in the Case Report Form.

- ***Local tolerance***

The investigator will assess local tolerance at Assessments Periods D_1 to D_{28}, by direct evaluation for erythema and desquamation, and through subject's interview for pruritus and burning accordingly to appendix IX.

7.8.2. Assessment of the Immune Response

Evaluation of the Tetanus and Influenza vaccine-specific :

- Antibody GMT: will be measured using a refernced method in two reference laboratories (Influenza: Centre National de Reference de la grippe, Institut Pasteur, Paris; Tetanus toxoid: Lab Microbiologie, Hôp Henri Mondor, Créteil, France)
- Peripheral blood CD4 and CD8 T cells will be measured by ELISpot-IFNgamma and intra-cellular staining (IFN-g and IL-2) in the Laboratoire d'Immunologie Cellulaire in Paris, according to a Standard Operating Procedure (See Appendix VII for SOP)

7.8.3. Subject's Instructions

During the trial, subjects won't be allowed to :

- Use any topical drug or cosmetic on the investigational sites (except usual cleaning products),
- Use any topical or systemic treatment, (drug or cosmetic), which may interfere with the trial assessment (anti-inflammatory drugs or immunomodulators)
- Perform intensive sport,
- Have sun exposure or UV sessions

- After the application of Tetagrip® vaccine, the volunteer will be instructed not to take a shower or bath and to avoid any activity which causes sweating or mechanical stress to the investigational site, e.g. physical exercise, during the following 24 hours.

7.8.4. Procedure for collection and shipping of blood samples

To be performed according to the Standard Operating procedure described in Appendix VI.

8. STUDY ENDPOINTS

8.1. Primary Endpoints

Clinical local and systemic tolerance to Tetagrip® vaccine administration for both transcutaneous (TC) and intramusculaire (i.m) routes of administration will be used as the primary measure of safety.

This criterion will be measured as the rate of severe adverse events (grade $\geq$ 3) judged by the investigator to be probably or definitely related to the transcutaneous mode of vaccine administration.

Safety will be evaluated by:

- Severe local toxicity at the site of injection (i.e., pain, tenderness, erythema, induration, regional lymphoadenopathy, limitation of limb movement) judged to be probably or definitely related to the mode of vaccine administration.
- Severe systemic symptoms (i.e.), fever, myalgia, fatigue, headache, anaphylaxis, hypersensitivity reactions) judged to be probably or definitely related to the administration mode of the vaccine.
- Other severe adverse reaction such as dermatologic, neurologic, gastrointestinal (nausea/vomiting, diarrhea) judged to be probably or definitely related to the administration mode of the vaccine

The criteria for an adverse experience to be considered severe are described in section10)

8.2. Secondary Endpoints

Evaluation of immunogenicity of the two modes of administration will be measured in all participating subjects.

Immune response against 0.5 ml Tetagrip® vaccine administered via transcutaneous application compared to the immune response induced by conventional i.m. injection will be assessed in the peripheral blood to evaluate:

- The protective tetanus and influenza-specific antibodies GMT titers.
- The increase in tetanus and influenza specific antibody titers between baseline and day 14 and day 28.
- The tetanus and influenza CD4 and CD8 peripheral blood T cells numbers.
- The characteristics of vaccine-specific CD4 and CD8 T cell differentiation.

9. STATISTICAL CONSIDERATIONS

9.1. General design issues

The primary aims of this study are to assess safety and tolerance of a transcutaneous mode of tetanus/influenza vaccine administration and to compare to the immunogenicity induced by intramuscular injection of the vaccine in healthy volunteers and HIV infected patients. The 48 participants will be randomized to two arms in two modes of vaccine administration; these groups are: Arm A) Transcutaneous administration of Tetagrip® vaccine and ArmB) Intramuscular injection of Tetagrip® vaccine.

9.2. Analysis Plan Overview

The data Analysis will consist of a comparison of safety and immunogenicity data between study arms. The primary analysis is *an intent to treat analysis,* i.e., it will include all participants enrolled. A per protocol analysis will also be conducted. Since enrolment is concurrent with receiving vaccination by any of the two administration modes, all participants will have received Tetagrip® vaccine, either by transcutaneous application or conventional intramuscular injection and therefore will provide some safety data.

9.3. Sample Size Calculations

Our intended group size of twelve individuals per arm in each cohort is usual for phase I trials and was not based on sample size calculations

Two or more severe toxicities within an arm will indicate a safety problem. If one observe no AE ($\geq$ grade 3) then, with this sample size (12 subjects within each arm) the true rate of occurrence of a severe toxicity event $\geq$ grade 3 related to the administration mode of Tetagrip® vaccine is lower than 26.5 %

9.4. Statistical Analysis

All statistical analysis will be performed using SAS or SSPS software. All randomized subjects who received one dose of the vaccine will be included in the ITT analysis.
The analysis variables consist of baselines variables, primary endpoints (safety variables), and secondary endpoints (immunogenicity variables).

9.4.1. Baseline

Demographic variables to be measured, include race, ethnicity, age, and sex.
Clinical variables including temperature, vital signs, and clinical symptoms.
Immunology variables will also be collected at baseline
For baseline variables, descriptive analyses will be performed (%, ;median, interquartiles, ranges) by arm in each cohort.

9.4.2. Primary endpoints (Safety)

Primary endpoints measure safety of the candidate transcutaneous administration mode. Toxicities for each arm are measured by local and systemic reactions to the mode of Tetagrip® vaccine administration.

9.4.3. Secondary endpoints (Immunogenicity analysis)

For safety and immunogenicity the two arms will be compared using non parametric tests: Fisher exact test for qualitative variables and Mann-Whitney tests for continuous variables.

10. ADVERSE EVENTS AND SERIOUS ADVERSE EVENTS

10.1. Adverse Events - Definition

An Adverse Event (AE) is any adverse change from the subject's baseline condition. This includes intercurrent signs, symptoms, illness, and significant deviations from baseline laboratory values, which may occur during the course of the clinical study, whether considered related to treatment or not.

All laboratory tests for which abnormal results are collected after study treatment initiation should be repeated until the values return to normal or stable status.

Abnormal results are defined as those falling out of the laboratory normal range that are clinically significant. The frequency with which such checks should be made will be defined by the investigator's opinion depending on the degree of the abnormality.

In all cases, the aetiology should, as much as possible, be identified and the sponsor notified.

10.1.1. Grading of adverse events

The severity of adverse events should be determined by using the toxicity criteria given in Appendix XI.

10.1.2. Reporting of adverse events

All adverse events which occurs between 2 visits must be assessed and documented using the toxicity guide. Subjects should be asked for recording any signs and symptoms; onto the patient diary card.

Any adverse or intercurrent event occurring during the study period, spontaneously reported by, or written onto the subject's diary card; or observed by others, will be recorded in the subject's Case Report Form (CRF). At each volunteer/patient visit, the investigator will inquire about any Adverse Event occurrence, by interviewing the volunteer using an open question taking care not to influence the subject's answer and, if appropriate, by directed questioning and clinical examination.

Each time a concomitant medication is reported during the study, an Adverse Event will be documented and the reason for the therapy noted.

The records will describe the nature (diagnosis, signs and symptoms) severity, date/time of onset, date/time of resolution, outcome and actions taken as well as relationship to study treatment (according to the investigator's opinion).

It will be specified whether the event is serious or not

All AEs already recorded and designated as "ongoing" should be reviewed at each subsequent visit. If resolved, the details are to be recorded in the CRF. If any AE changes for the worse, in frequency, symptoms or in severity, a new record of the event must be started. Distinct reports

are required for differing frequencies and/or severity of the same event to enable comprehensive safety reports and later analysis.

10.2. Serious Adverse Events - Definition

A serious adverse event (SAE) includes but is not necessarily restricted to any event which:

- Results in death (whatever may be the cause)
- Is life-threatening
- Results in persistent or significant disability/incapacity
- Requires hospitalization or prolongation of existing hospitalization
- Is a congenital anomaly or birth defect
- Other events including cancer, overdose, pregnancy and any additional adverse experience or abnormal laboratory values occurring during the study period defined by the protocol as serious or which the investigator considers significant enough or that suggests a significant hazard, contraindications, side effect or precaution will be handled as a serious adverse event.

The term “life threatening” in the definition of “***Serious***” refers to an event in which the patient was at risk of death at the time of the event; it does not refer to an event which hypothetically might have caused death if it was more severe.

Hospitalization solely for the purpose of diagnostic tests, even if related to an Adverse Event, elective hospitalization for an intervention which was already planned before the inclusion of the subject in the study, and admission to day-care facility may not themselves constitute sufficient grounds to be considered as a Serious Adverse Event.

The severity of illness and adverse events are categorized using a standard grading scale with the following severity levels: mild, moderate, severe, life threatening, and death.

Intensity will be determined, using the following definitions as guideline :

- ***Mild***: Awareness of sign or symptom, but easily tolerated, has not to be treated and is not affecting daily life,
- ***Moderate***: Discomfort enough to cause interference with daily activity, but with no risk for the subject’s health and improvement after slight therapy,
- ***Severe***: Unbearable event, interfering considerably with the subject’s daily activity and/or possibly leading to a disability, or a life-threatening situation.

The causal relationship of an Adverse Event to the investigational product(s) will also be determined by the investigator according to the following criteria :

- ***Unlikely***: The event, with predominant probability, is due to other reasons than the administration of the investigational product(s).
- ***Possible:*** There is sufficient information to accept the possibility of a causal relationship, although the connection is uncertain or doubtful, i.e. a causal relationship is not impossible and not unlikely. The event follows a plausible chronological

sequence in respect of administering the investigational product(s) and or presents the usual response with the product(s) tested. It might also be caused by other factors such as the subject's clinical condition, therapeutic procedures or administration of concomitant medications.

- ***Probable***: There are good reasons and sufficient documentation to assume a causal relationship in the sense of plausible, conceivable, likely, but not necessarily highly probable. The event follows a plausible chronological sequence in respect of administering the investigational product(s) and/or presents an expected response of the investigational product(s) and cannot reasonably be explained by other factors such as the subject's clinical condition, therapeutic procedures or administration of concomitant medications.
- ***Definitively related****:* Should be reserved for those events which have no uncertainty in their relationship to investigational product administration : this means that are-challenge was positive.
- ***Unclassifiable:*** Causality is, for one or another reason, not assessable, e.g. because of insufficient evidence or conflicting data. The event can neither be attributed to the investigational product(s) nor to any other factor because of a lack of objective evidence.

Unexpected adverse drug reaction is defined as:

An adverse reaction, the nature or severity of which is not consistent with the applicable product information (e.g. Investigator Brochure for an unapproved investigational product or package insert / summary of product characteristics for an approve product)

10.2.1. Grading of serious adverse events

Safety of the transcutaneous mode of vaccination under investigation will be assessed by monitoring clinical and immunological parameters and by the occurrence of Grade 3 (severe) or Grade 4 (life-threatening) local and systemic reactions to the Tetagrip® vaccine administration by using two distinct modes of administration.

Adverse events may be temporarily incapacitating (for example, loss or cancellation of work or social activities) and could make the administration mode under investigation impractical for large scale use if they occur in more than a small proportion of cases. Separate assessments of the rates of severe systemic and local reactions will be made. All participants will be closely monitored to compare in both arms, local and systemic adverse events during the week following Tetagrip® vaccine administration, and followed for 4 weeks.

The relationship of these experiences to vaccination by using two administration routes will be assessed and recorded as one as the following: definitely related, probably related, possibly related, or not related.

Adverse events will be judged as Serious Adverse Events (SAEs) using the criteria given in appendix XI.

10.2.2. Reporting of serious adverse events

All serious Adverse Events occurring during treatment period and 30 days after the end of study treatment evaluation, according to the above mentioned definitions, regardless of treatment or relation to study administration route and study drug must be recorded by the investigator as soon as he/she is informed of the event.

The investigator must notify ORVACS of this event by sending within **24 hours** the "Notification of Serious Adverse Event" form (initial report; Appendix XII) with all available information concerning the event to the sponsor's representatives :

Project leader/Study manager

Norma WINCKER, PhD.
ORVACS
Hôpital Pitié Salpêtrière
Pavillon Laveran
47-83 boulevard de l'hôpital
75013 Paris, FRANCE
Phone: + 33 1 44 24 23 98
Fax + 33 1 42 16 17 96 / + 33 1 44 24 01 26

In her absence, the Data Management Department will be contacted :

ORVACS
Tchadie BOMMENEL
INSERM U720
Phone: + 33 1 42 16 42 59
Fax + 33 1 42 16 42 61 / 67

10.2.3. Follow-up of Adverse Event

Any serious and unexpected adverse event should be medically well documented and the information should be available as soon as possible. The investigator must complete a special clinical report to describe the outcome. Subject withdrawal, investigational product discontinuation and re-introduction or permanent discontinuation will also be documented for each event.

The Serious Adverse Events must be followed-up until resolution or stabilisation or until evidence that the investigational route and/or product and/or the volunteer's participation in the trial are not responsible for the event. A Serious Adverse Event which would not be resolved at the end of the trial will have to be followed until resolution or stabilisation.

Subjects who are removed from the trial due to a SAE will be treated according to established medical practice. All pertinent information concerning the outcome of such treatment must be entered in the Case Report Form. If the Adverse Event has not been resolved at the end of the trial, a follow-up is required at appropriate intervals until recovery, unless a plausible, investigational product-unrelated explanation has been found.

Any Serious Adverse Events shall be also reported in the CRF.

11. STUDY MONITORING AND DATA COLLECTION

11.1. Data collection

11.1.1. CRF

All data obtained in the study described in this protocol will be recorded on CRFs. The CRF for each subject will be presented in a folder. The CRF will be completed chronologically and updated regularly in order to reflect the most recent data on the subject included in the study.

Prior to the start of the study, the investigator will complete a “Delegation of significant study related duties” or “List of delegation tasks” form, showing the signatures and initials of all those who are authorized to make or change entries on the CRF.

Procedure to fill-in CRFs:

Each CRF must be neatly filled in with a black-inked pen. For each page on which information is entered, the subject code must be recorded.All data generated in the course of the trial will be recorded. Incomplete entries must be substantiated by giving reasons.

- Write one digit per box. If a value has fewer digits than the number of boxes, please right-justify.
- If a pre-defined code is given, please tick the appropriate item or complete it.
- Write data in the form of PRINTED CAPITAL LETTERS.
- If an answer cannot be provided please fill in NA or ND.

  NA will be used for : *not applicable, not known, not available*,

  ND will be used for: *not done*.
- Errors must be corrected by drawing a single line through the incorrect entry and by writing the new value as close as possible to the original. The correction must then be initialled and dated by an authorized person.
- Photographs and any electronically issued documents (if there are) will be identified with the subject’s Number and initials and filed in the CRF.
- The registration form and the end of study form must be dated and signed by an authorized investigator

Although subjects may be interviewed by a research nurse or the trained equivalent (e.g. medical student, physician assistant), the investigator must verify that all data entries are accurate and correct, including verification that the subject fulfils the criteria for entrance into the study before vaccine administration. Physical examinations have to be performed by a registered medical practitioner.

11.1.2. Source documents

Definition:

- Source Data: All information in original records and certified copies of original records of clinical findings, observations, or other activities in a clinical trial necessary for the reconstruction and evaluation of the trial. Source data are contained in source documents.

- Source Documents: Original documents, data, aand records (e.g. hospital records, clinical and office charts, laboratory notes, memoranda, records, recorded data from automated appliances, copioes or transcriptions certified after verification as being accurate copies, microfiches, photographic negatives, microfilms or magnetic media, x-rays, subject files, and records kept at the pharmacy, at the laboratories and at medico-technical separtments involved in the clinical trial).

The subject must have consented to allow their medical records to be viewed by sponsor-authorized personnel and by local and possibly foreign regulatory authorities. This information shall be included in the informed consent.

11.2. Study monitoring

A monitor will be assigned by the sponsor to monitor this study and periodically contact the site, including conducting on site visits.

Monitor's activities will include:

- Site initiation visit to collect and distribute essential pre-study documents; to instruct site personnel about the protocol, study procedures and expectations; to obtain the investigator's assurance to comply with study requiorements and GCP guidelines and inform site personnel about study materials.

- Monitoring visits: According to Good Clinical Practices, the study monitor are fully instructed concerning confidentiality and able to perform any necessary control on Informed Consent and CRFs. All observations and findings should be verifiable. During monitoring visists, the sponsor's monitor will:

- Check and assess the progress of the study,
- Review collected study data,
- Conduct Source Document verification (hospital files),
- Identify any issue and address its resolution

All of that will be done in order to verify that the:

- Data are authentic, accurate and complete,
- Safety and rights of subjects are being protected,
- Study is conducted in accordance with the currently approved protocol (and any amendements), GCP and all applicable regulatory requirements.

The investigator agrees to allow sponsor's monitor direct access to all relevant documents and to allocate his/her time and the time of his/her staff to the monitor to discuss findings and any relevant issues.

- Termination visit: At study closure the monitor will also conduct the related activities.

12. DATA MANAGEMENT

12.1. Data entry

The study data will be entered into a data base at the end of the study. Independent double data entry will be performed by two different trained operators. The two entries will be compared in order to identify and resolve any data entry discrepancy.

12.2. Data review

Consistency checks will be performed on the data. The resulting edit queries will be transmitted to the monitoring team. Answers to these queries will be integrated into the data base.

12.3. Data coding

Adverse events will be coded according table in appendix XIV.
Serious Adverse Events will be coded according to MedDRA

12.4. Data storage

Data backup will be done on CD-Rom

12.5. Data freezing

After corrections and modifications have been performed, the data base will be locked. Data will be extracted from the data base into the data files for statistical analysis.

13. ETHICAL CONSIDERATIONS

13.1. Ethical conditions

This study will be performed in accordance with the principles stated in the Declaration of Helsinki (appendix II) and subsequent amendments and in accordance with the Good Clinical Practice Guideline (CPMP/ICH/135/95) and in compliance with local regulatory requirements

Independent Ethics Committe and Health authorities

- **Independent Ethics Committee (IEC)**

Before beginning the trial, the Investigator will submit to the IEC of the University Medical Center Charité, Campus Mitte, a study file. The clinical trial won't begin before obtaining the approval of the IEC on the study protocol as submitted, or after being implemented with the modifications requested by the IEC.
The study documentation will also be submitted to the ethical committee of the Goethe University according with new regulations. Any appropriate amendment to the protocol will also be submitted to the IECs.

- **Health Authorities**

The sponsor will submit the study protocol
In Germay: To the Paul-Ehrlich Institute (Federal Agency for Sera and Vaccines) and to the local ethic committee of the principal investigator in Berlin
In France: To the French Agence for the medicines (AFSSAPS) and the CCPPRB of the Pitié Salpêtrière hospital.

13.2. Patient information and consent

The investigator will explain to the subjects the objectives and methods of the clinical trial as well as the potential risks of the trial and of the possible discomforts which might happen to them.

They will be informed that they can choose not to participate in the trial and that they are free to retrieve their consent at any time without having any disadvantage.

A written document, called "Subject Information Sheet and Consent Form", approved by the IEC, written in German in an easily understanding wording, will be handed over to the subject by the investigator. After reading, understanding and having the responses to all the questions, the subject will give his written consent twice by initialing each page and dating and signing the last page.

By signing the same form at the bottom of the page, the investigator will confirm that he/she has provided all information contained in the form prepared for this trial.

The signed consent will be obtained before engaging any trial procedure with the subject.

One copy is intended for the subject, and the original for the investigator's source document file.

The Subject Information Sheet and Consent Form for each participating center is filed in Appendix I.

13.3. Confidentiality

The aim and content of this trial, its investigational products and its results must not be disclosed, other than to those directly involved in the conduct or ethical review of the trial, without written authorization ***by ORVACS.***

All information from this study (excluding data from informed consent) will be entered into a computer by the sponsor in accordance with the French law "Loi informatique te libertés" (Art. 40, January 6, 1978) modified by the law dated August 6, 2004 transposing the European Directive 95/46/CE.

13.4. Insurance Policy

Insurance will be provided by ORVACS. A Copy of the Insurance certificate is filed in Appendix III.

13.5. Compensation

Healthy volunteers will receive a compensation of 250 Euros for their participation in the trial. This compensation has been calculated on the number of visits, blood withdrawals and on the time they will spend at the Center at each visit. Additionally, public transportation costs within the city will be covered for a maximum of 40 Euros during the whole study.

14. ADMINISTRATIVE PROCEDURES

14.1. Protocol amendments

Neither the investigator nor the sponsor may alter the protocol without the permission of the other parties.
All changes to the protocol will be subject to an amendment which must be dated and signed by both parties (Investigator and Sponsor) and must appear as an addendum to the protocol.
Depending on the importance of the change in the conditions of the trial, the amendment will be sent to the IEC for prior approval or for information. It will be also submitted or sent to the relevant regulatory authorities if applicable.

14.2. Audit

14.2.1. Source document requirement

It is the responsibility of the investigator to assure that

- the trial is conducted in accordance with the protocol,
- valid data are entered into the Case Report Form,

- a documentation of all relevant data in the subject's file is maintained, such as medical history, concomitant diseases, date of inclusion, visit dates, results of examinations, administration of investigational products, Adverse Events and any change in concomitant medication

To achieve this objective, the investigator will permit auditor to monitor the trial and check the Case Report Forms, Informed consent and related source documents during regular visits in order to determine that data recording and protocol adherence are satisfactory. The investigator and his staff will be expected to cooperate with the auditor providing any missing information whenever possible.

In addition, the investigator guarantees:

- Prior to each monitoring visit all data should be properly record in the Case Report Forms,
- At the monitoring visits source documentation should be available, (in case of electronic medical file, a dated and signed printed copy will be sorted out and filed within the CRF).

14.2.2. Inspections by regulatory agencies

By signing this protocol, the investigator agrees to allow any regulatory agency to have access to the study records for review. These personnel, bound by professional secrecy, will not disclose any personal identity or personal medical information. These audits involve review of source documents supporting the adequacy and accuracy of data gathered in CRFs, review of documentation required to be maintained, and checks on investigational products accountability. The investigator will have to be available to provide the auditors with all the requested documents and be available to answer to any questions.

In case of an audit, an audit certificate will be issued and joined to the final report.

14.3. Publications

Prior to publication ORVACS will receive a final report co-established by principal investigator clinical study investigator, and immunology study coordinator and the statistical data management center, in due time after the end of the study. The results will be published by the principal investigators and coworkers in a peer-reviewed journal after agreement between sponsor representative, principal investigator clinical investigator, and immunology study coordinator. Neither investigators nor the sponsor may publish without the permission of other parties.

14.4. Clinical Study Report

At completion of the data analysis, a final report will be drawn up. This report will be a clinical and statistical integrated report. This report will be signed by the Sponsor representative(s) and the clinical study investigators.

14.5. Source Documents, Investigator's file storage

All trial-related documents must be kept by the investigator in appropriate file folders. Records of subjects, original informed consents, source documents, Case Report Forms, photographs, correspondence, original or amended signed protocol, IEC and Sponsor correspondence pertaining to the study, all reports and all other material relating to the study must be kept securely on file for the legally required duration of archiving.

If the investigator retires, relocates, or for any other reasons withdraws from the responsibilities of keeping the study records, custody must be transferred to a person who will accept the responsibility. The Sponsor must be notified in writing of the name and address of the new custodian.

15. REFERENCES

Autran B., Debré P., Walker B., Katlama C. Therapeutic vaccines against HIV need international partnerships. Nat Rev Immunol, 3(6) :503-508, 2003

Autran, B., Carcelain, G., T.S., Li., Blanc, C., Mathez, D. Positive effects of combined anti-retroviral therapy on CD4+ T cell homeostasis and function in advanced HIV disease. Science 277, 112-116, 1997

Christoph T, Muller-Rover S, Audring H, Tobin DJ, Hermes B, Cotsarelis G, Ruckert R, Paus R: The human hair follicle immune system: cellular composition and immune privilege. Br J Dermatol 142(5):862-873, 2000

Fan H, Lin Q, Morrissey GR, Khavari PA: Immunization via hair follicles by topical application of naked DNA to normal skin. Nat Biotechnol 17(9):870-872, 1999

Glenn GM, Taylor DN, Li X, Frankel S, Montemarano A, Alving CR: Transcutaneous immunization: a human vaccine delivery strategy using a patch. Nat Med 6(12):1403-1406, 2000

Glenn GM, Kenney RT, Hammond SA, Ellingsworth LR. Transcutaneous immunization and immunostimulant strategies. Immunol Allergy Clin North Am 23(4):787-813, 2003

Hueber F, Schaefer H, Wepierre J: Role of transepidermal and transfollicular routes in percutaneous absorption of steroids: in vitro studies on human skin. Skin Pharmacol 7(5):237-244, 1994

Jodar L, Duclos P, Milstien JB, Griffiths E, Aguado MT, Clements CJ: Ensuring vaccine safety in immunization programmes--a WHO perspective. Vaccine 19(13-14):1594-1605, 2001

Kenney RT, Frech SA, Muenz LR, Villar CP, Glenn GM: Dose sparing with intradermal injection of influenza vaccine. N Engl J Med 351(22):2295-2301, 2004

Lademann J, Otberg N, Richter H, Weigmann HJ, Lindemann U, Schaefer H, Sterry W: Investigation of follicular penetration of topically applied substances. Skin Pharmacol Appl Skin Physiol 14 Suppl 1:17-22, 2001

Lange CG, Lederman MM, Medvik K, Asaad R, Wild M, Kalayjian R, Valdez H. Nadir CD4+ T-cell count and numbers of CD28+ CD4+ T-cells predict functional responses to immunizations in chronic HIV-1 infection. AIDS. 26;17(14):2015-23, 2003

DermaVir: a novel topical vaccine for HIV/AIDS.Lisziewicz J, Trocio J, Whitman L, Varga G, Xu J, Bakare N, Erbacher P, Fox C, Woodward R, Markham P, Arya S, Behr JP, Lori F. J Invest Dermatol. 2005;124(1):160-9

McMichael A and T. Hanke: HIV vaccines 1983-2003. Nature Med. 2003 ,7: 874-880

Mills OH Jr, Kligman AM. The follicular biopsy. Dermatologica 167(2):57-63, 1983

Partidos CD, Beignon AS, Mawas F, Belliard G, Briand JP, Muller S: Immunity under the skin: potential application for topical delivery of vaccines. Vaccine 21(7-8):776-780, 2003

Schaefer H, Lademann J: The role of follicular penetration. A differential view. Skin Pharmacol Appl Skin Physiol 14 (Suppl 1):23-27, 2001

Taira K, Narisawa Y, Nakafusa J, Misago N, Tanaka T. Spatial relationship between Merkel cells and Langerhans cells in human hair follicles. J Dermatol Sci 30(3):195-204, 2002

Tenjarla SN, Kasina R, Puranajoti P, Omar MS, Harris WT: Synthesis and evaluation of N-acetylprolinate esters - novel skin penetration enhancers. Int J Pharm 192(2):147-158, 1999

APPENDIX I

PATIENT INFORMED CONSENT FOR GERMAN CENTERS

PATIENTENINFORMATION

KLINISCHEN STUDIE ZUR BEWERTUNG DER SICHERHEIT UND DER WIRKSAMKEIT DER APPLIKATION VON 0,5 ML TETAGRIP®-IMPFSTOFF (IMPFSTOFF GEGEN TETANUS UND GRIPPE) AUF DIE HAUT IM VERGLEICH ZUR INTRAMUSKULÄREN INJEKTION IN DEN OBERARM,
IN INSGESAMT VIER GRUPPEN:
ZWEI GRUPPEN MIT JE 12 GESUNDEN PROBANDEN UND
ZWEI GRUPPEN MIT JE 12 HIV-INFIZIERTEN PATIENTEN
KLINISCHE STUDIE: MANON 05 – CUTAVAC

Proband / Patient:______________________
Familienname, Vorname

Probanden / Patienten -Nr.:______________________

Sehr geehrter Proband, sehr geehrter Patient,

Der Untersucher ______________________________________(Familienname, Vorname), (verantwortlicher Arzt zur Durchführung der o. a. klinischen Studie) hat Sie um Ihr Einverständnis gebeten, ob Sie an der o. a. klinischen Studie teilnehmen wollen.

Die klinische Studie wird in drei Zentren, dem „Clinical Research Center for Hair and Skin Physiology“ der Klinik für Universitätshautklinik, Charité – Universitätsmedizin Berlin, Schumannstr. 20/21, 10117 Berlin, dem Klinikum der Johann Wolfang Goethe-Universität Frankfurt, Innere Medizin II, Theodor-Stern-Kai 7, 60590 Frankfurt am Main und dem Service des Maladies Infectieuses, Hôpital Pitié Salpêtrière, Pavillon Laveran, 47-83 Bd de l’hôpital, 75651 Paris, Frankreich, durchgeführt. Bevor die klinische Studie beginnen kann, ist es unabdinglich, dass Sie als freiwilliger Proband / Patient Ihr schriftliches Einverständnis geben, um an der o. a. klinischen Studie teilzunehmen.

Bitte lesen Sie die folgende Information sorgfältig durch, um sich auf das Gespräch mit dem untersuchenden Arzt vorzubereiten. Bitte zögern Sie auf keinen Fall, wenn Sie Fragen haben, die Ihnen wichtig erscheinen.

Sie sollten die Einverständniserklärung nur unterschreiben, wenn Ihnen die Bedeutung und die praktischen Details dieser Studie verständlich geworden sind, wenn Sie einverstanden sind, an ihr teilzunehmen und wenn Ihnen klar ist, welche Rechte und welche Verpflichtungen Ihnen als freiwilliger Proband / Patient an dieser klinischen Studie obliegen.

Das Studienprotokoll und alle studienrelevanten Unterlagen wurden der Ethikkommission der Charité – Universitätsmedizin Berlin Campus Mitte, die aus unabhängigen Mitgliedern ohne direktes Interesse an der Durchführung dieser Studie besteht, vorgelegt. Dem Leiter der klinischen Studie liegt eine Kopie der Zulassung und Anerkennung durch die Ethikkommission vor. Studienprotokoll und alle

studienrelevanten Unterlagen sind auch dem ethischen Ausschuß des Klinikum an der Johann Wolfgang Goethe Universität dargestellt worden.

ZIELE UND RECHTFERTIGUNG FÜR DIESE KLINISCHE STUDIE

Die Infektion mit dem Human Immundeficiency Virus (HIV) ist eine chronische Infektion. Antiretrovirale Medikamente helfen in einem Teil der Betroffenen, das Virus zu kontrollieren. Diese müssen jedoch lebenslang eingenommen werden und haben häufig beeinträchtigende Nebenwirkungen. Verschiedene internationale Arbeitsgruppen haben in den vergangenen Jahren Impfstoffe gegen HIV experimentell und klinisch getestet. Trotz viel versprechender Ergebnisse ist es bislang nicht gelungen, einen effektiven Impfstoff zu entwickeln. Eine mögliche Erklärung liegt darin, dass durch die üblicherweise durchgeführte Injektion von Impfstoff in Muskelgewebe oder in subkutanes Fettgewebe der Impfstoff in Gewebe gebracht wird, das arm an Immunzellen sind.
Unsere Forschung zielt darauf ab, ein neuartiges Impfverfahren zu evaluieren, bei dem Impfstoff über die Haut eingebracht wird. Die menschliche Haut ist ein wichtiges Grenzorgan. Sie steht in ständigem Kontakt mit Krankheitserregern und ist dementsprechend sehr reich an Immunzellen. Diese Immunzellen sind optimal auf den Kontakt mit Fremdmaterial vorbereitet. Impfverfahren, die den Impfstoff direkt in die Nähe dieser Hautimmunzellen bringen, nutzen die natürliche Schutzfunktion der Haut und könnten die Wirksamkeit von Impfstoffen deutlich verbessern. Verschiedene Studien an Tiermodellen und am Menschen haben bereits gezeigt, dass Impfungen direkt durch die Haut möglich sind. Derartige Impfverfahren würden konventionelle Injektionen, die schmerzhaft sind und mit einem Infektionsrisiko einhergehen, überflüssig machen.
Das in unserer Arbeitsgruppe entwickelte Verfahren zum Auftragen von Impfstoff auf die Haut basiert auf der Beobachtung, dass ein beträchtlicher Anteil von Substanz, die aufgetragen wird, durch die Haarfollikel in die Haut gelangt. Die Haarfollikel stellen wichtige natürliche Unterbrechungen in der sonst weitgehend durchgehenden und dichten Hornschicht der Haut dar. Ein Großteil der Haarfollikelöffnungen ist allerdings durch Talg und abgestoßene Hautzellen verschlossen. Die Entfernung dieser Talg-Horn-Pfröpfe ist durch die sog. Cyanacrylat-Stripping-Methode möglich, bei der eine hauchdünne Schicht Sekundenkleber auf die Hornschicht der Haut aufgebracht und nach Antrocknen mit Hilfe von Tesa-Film entfernt wird. Es werden durch diese Technik nur die obersten Hornschichten (etwa 30% der gesamten Hornschicht) abgetragen. Die Haut an sich bleibt unverletzt.
Im Rahmen der vorgestellten Studie sollen mit Hilfe des Testimpfstoffs Tetagrip® (Impfstoff gegen Tetanus und Grippe) die Sicherheit, die Verträglichkeit und die Effektivität des in unserer Arbeitsgruppe entwickelten Verfahrens zum Auftragen von Impfstoff auf die Haut an gesunden Probanden und an HIV-infizierten Probanden untersucht werden.

STUDIENBESCHREIBUNG

Die vorgestellte Studie umfasst zwei Kohorten, d.h. 24 gesunde Probanden (Kohorte I Berlin) und 24 HIV-infizierte Patienten (Kohorte II Frankfurt, Paris). Jeweils 12 Probanden / Patienten jeder Kohorte erhalten 0,5 ml Tetagrip®-Impfstoff (Impfstoff gegen Tetanus und Grippe) intramuskulär, d.h. durch Injektion in das Muskelgewebe der linken Oberarmaußenseite. Das Vorgehen entspricht der zugelassenen und routinemäßig durchgeführten Vorgehensweise. Entsprechend ist mit einer sicheren Immunisierung dieser Probanden / Patienten gegen Tetanus und Grippe zu rechnen. Die verbleibenden 12 Probanden / Patienten jeder Kohorte erhalten 0,5 ml Tetagrip®-Impfstoff verteilt auf zwei Hautareale von je 4x 4 cm auf der linken Oberarmaußenseite. Diese beiden Hautareale werden gemäß der in unserer Forschungsgruppe erarbeiteten Methode mit Cyanacrylat Stripping vorbehandelt. Untersucht wird die Verträglichkeit der Applikation des Impfstoffs auf die Haut. Die Immunantwort bei Probanden /

Patienten, die den Impfstoff auf die Haut erhalten, wird mit der Immunantwort verglichen, die in den übrigen Probanden / Patienten durch intramuskuläre Injektion von Tetagrip® Impfstoff erreicht wird. Die Studiendauer umfasst 5 Wochen pro Proband / Patient.

INFORMATION ÜBER DIE UNTERSUCHUNGSPRODUKTE UND MÖGLICHE RISIKEN UND UNERWÜNSCHTE WIRKUNGEN

Der Impfstoff Tetagrip® ist ein in Frankreich zur subkutanen und zur intramuskulären Injektion zugelassener und routinemäßig verwendeter Impfstoff, der von der Firma Sanofi-Pasteur hergestellt wird. Zur Impfung gegen Tetanus und gegen Grippe erfolgt üblicherweise eine einmalige Injektion von 0,5 ml des Impfstoffs in die Oberarmaußenseite. Auf diese Weise werden 0,5 ml Impfstoff das Muskelgewebe der Oberarmaußenseite eingebracht. Diese intramuskuläre Injektion entspricht der zugelassenen und routinemäßig durchgeführten Vorgehensweise.. Die Impfung ist im Allgemeinen sehr gut verträglich. Möglich sind leichte Reaktionen an der Injektionsstelle und Allgemeinreaktionen, wie Abgeschlagenheit, Magen-Darm-Beschwerden oder Temperaturerhöhungen. Es besteht allgemein die Möglichkeit einer Überempfindlichkeitsreaktion gegen Bestandteile des Impfstoffes, Pflaster oder Kleberinhaltsstoffe. Individuen mit bekannter Überempfindlichkeit gegen derartige Stoffen werden nicht in die Studie aufgenommen. Trotzdem erfolgt eine Kontrolle des Applikationsareals an den Tagen 1, 7, 14, 21 und 28 nach der Impfung. Eine vorübergehende Schwellung der lokalen Lymphknoten in der Achsel kann auftreten. Auch diesbezüglich wird der Proband / Patient bei jedem Kontrolltermin untersucht.

EINSCHLUSSVERFAHREN

Wenn Sie Interesse an einer Teilnahme an der vorgestellten Studie haben, bitten wie Sie zu einer Screening-Untersuchung. Am Tag des Screenings (Tag -7, 1 Woche vor Studienbeginn) wird Ihre Krankengeschichte einschließlich der eingenommenen Medikamente und der bisher erhaltenen Schutzimpfungen erhoben. Anhand der Einschlusskriterien wird Ihre Eignung für die klinische Studie überprüft. Teilnehmer dieser Studie müssen z. B. zwischen 18 und 45 Jahren alt sein und einen Body Mass Index (Körpergewicht (kg) / Körpergröße^2 (cm^2)) von 21-26 aufweisen. Für einen Einschluss in die Studie muss die letzte Tetanus-Impfung mindestens 4 Jahre zurückliegen und die letzte Grippe-Impfung mindestens ein Jahr.
Weitere Einschlussbedingungen sind für gesunde Probanden ein negativer HIV-Test innerhalb der letzten 3 Monate. Spezielle Hinweise für HIV-infizierte Patienten sind im folgenden Abschnitt aufgeführt. Die Zuordnung der jeweiligen Applikationsform des Impfstoffs, d. h. entweder transkutan oder intramuskulär, erfolgt randomisiert, d. h. nach dem Zufallsprinzip.

HINWEISE FÜR HIV-INFIZIERTE PATIENTEN (KOHORTE II)

HIV-infizierte Patienten können an der Studie teilnehmen, wenn die Infektion erfolgreich mittels antiretroviraler Therapie kontrolliert ist. Dies beinhaltet eine Anzahl von CD4 Zellen von größer 350 Zellen/mm^3 über einen Zeitraum von mindestens einem Jahr, wobei der vorangegangene Tiefststand von CD4 Zellen nicht unter 200 Zellen /mm^3 liegen darf. Die antiretrovirale Therapie wird während der Studie beibehalten. Zur Bestimmung der beschriebenen Laborwerte sind Blutentnahmen von jeweils ca. 35-70 ml notwendig. Ein Teil der Blutproben wird für zusätzliche immunologische Untersuchungen verwendet, die durch den Sponsor dieser Studie ORVACS (Objectif Recherche Vaccin Sida, Programm zur Entwicklung von Impfstoffen gegen HIV) gefördert werden. An diesen Proben werden keine genetischen Untersuchungen durchgeführt.

UNTERSUCHUNGSMETHODEN UND DURCHFÜHRUNG

Tag -7 (1 Woche vor Studienbeginn)
- Schriftliche Einverständniserklärung
- Erst nach schriftlichem Einverständnis Überprüfung sämtlicher Einschluss- und Ausschlusskriterien
- Erhebung der Krankengeschichte einschließlich vorangegangener Impfungen und der aktuellen Medikation
- Zuordnung des Identifizierungscodes

Tag 0
- Körperliche Untersuchung
- Blutentnahme zur Bestimmung der Immunantworten gegen Grippe und Tetanus. In HIV-positiven Patienten erfolgt zusätzliche eine Bestimmung von Viruslast und CD4-Zahl
- Messung hautphysiologischer Eigenschaften (pH, Wasserverlust durch die Haut, Talgproduktion, Wassergehalt der Hornschicht)

- **Applikation von Tetagrip®-Impfstoff je nach Testgruppe:**
  An Tag 0 sind je nach Testgruppe ca. 1 Stunde bei intramuskulärer Applikation und ca. 2 ½ Stunden bei Auftragen von Tetagrip®-Impfstoff auf die Haut einzuplanen.

  Gruppe 1: Applikation auf die Haut:
  - Bestimmung von zwei Hautarealen von je 4x 4cm auf der linken Oberarmaußenseite
  - Markierung mittels permanentem Hautmarker
  - Rasur der Hautareale, jeweils 1 cm seitlich und je 2 cm nach oben und unten hin über die Testareale hinaus
  - Vorbereitung der Haut durch Cyanacrylat-Stripping: Auftragen einer dünnen Schicht Sekundenkleber auf den Testarealen, zusätzlich Applikation von Tesa-Film. Nach Aushärten des Sekundenklebers abziehen mit Hilfe des Tesa-Films zur Öffnung des Haarfollikelöffnungen
  - Applikation von je 0,25 ml Tetagrip®-Impfstoff und Massage des Hautareals mittels Fingerhandschuh
  - Nach Beendigung der Prozedur Applikation eines hydrokolloidalen Verbandes für 24 Stunden

  Gruppe 2: Injektion intramuskulär:
  - Bestimmung der Injektionsstelle an der Außenseite des linken Oberarms
  - Desinfektion der Injektionsstelle
  - Intramuskuläre Injektion von 0,5 ml Tetagrip®-Impfstoff

Verlaufskontrollen:
Sämtliche Probanden und Patienten unterziehen sich über einen Zeitraum von vier Wochen insgesamt 5 Kontrolluntersuchungen. Die erste Kontrolluntersuchung erfolgt an Tag 1.

Tag 1
- Entfernung des Verbandes
- Begutachtung der Applikationsstelle, Untersuchung der axillären Lymphknoten
- Frage nach Zeichen von Unverträglichkeit, Frage nach Veränderungen der Begleitmedikation
- Blutentnahme

Tag 3, Tag 7, Tag 14, Tag 21

- Begutachtung der Applikationsstelle, Untersuchung der axillären Lymphknoten,
- Frage nach Zeichen von Unverträglichkeit, Frage nach Veränderungen der Begleitmedikation
- Blutentnahme

Tag 28 - Ende der Studie

- Begutachtung der Applikationsstelle, Untersuchung der axillären Lymphknoten,
- Frage nach Zeichen von Unverträglichkeit, Frage nach Veränderungen der Begleitmedikation
- Abschließende körperliche Untersuchung
- Blutentnahme zur Bestimmung der Immunantworten gegen Grippe und Tetanus. In HIV-positiven Patienten erfolgt zusätzliche eine Bestimmung von Viruslast und CD4-Zahl

SPEZIELLE ANWEISUNGEN

Die Studiendauer pro Patient beträgt 5 Wochen (1 Woche Screening, 4 Wochen Verlaufskontrollen).

Geplant sind neben der Einschlussuntersuchung (Tag -7) sechs Untersuchungstage (Applikation des Impfstoffes an Tag 0, Verlaufskontrollen, bzw. Blutentnahmen an Tag 1,3,14,21 und 28)

- In den 24 Stunden nach Applikation des Impfstoffes sind sämtliche Aktivitäten zu vermeiden, die mit Schweißbildung verbunden sind; darunter jede Art von körperlicher Aktivität, Saunabesuche, etc.
- Während der gesamten Studiendauer sollte kein intensiver Sport getrieben werden.
- Abgesehen von üblichen Reinigungsprodukten (Duschgel und/oder Seife) dürfen Sie für die Dauer der Studie kein anderes Produkt auf die Testzonen am Oberarm auftragen.
- Des weiteren sollten Sie keine lokale oder innerliche Therapie durchführen, die die Studie beeinflussen könnten (darunter z. B. Medikamente, die die Funktion des Immunsystems beeinflussen; dies bespricht der Studienarzt mit Ihnen)
- Außer in dringenden Fällen sollten Se keine neue medikamentöse Behandlung beginnen. Sollten Se sich im Studienverlauf irgendeiner Behandlung unterziehen, so informieren Sie bitte Ihren Studienleiter möglichst umgehend, am besten noch ehe Sie mit der neuen Medikation beginnen.
- Sollten sie stets Sonneneinstrahlung auf die behandelten Hautpartien vermeiden und dürfen sich für die Studiendauer keiner UV-Bestrahlung (Solarium) aussetzen.
- Sie bekommen ein Tagebuch ausgehändigt, das alle Studienanleitungen zusammenfasst. Alle medizinischen Vorkommnisse sollten in diesem Tagebuch eingetragen werden. Bitte bringen Sie dieses Tagebuch zu jeder Kontrolluntersuchung mit.
- Sollten Sie an der Studie teilnehmen, werden Sie nach dem Zufallsprinzip einer der beiden Gruppen zugeordnet. Je nach Testgruppe erhalten Sie 0,5 ml Tetagrip®-Impfstoff als intramuskuläre Injektion in die Außenseite des linken Oberarms, oder es werden zwei Testareale von je 4x 4 cm auf der linken Oberarmaußenseite festgelegt die mittels Cyanacrylat-Stripping vorbehandelt werden und auf die nachfolgend insgesamt 0,5 ml Tetagrip®-Impfstoff aufgetragen wird.

NUTZEN

Bei Probanden / Patienten, die Tetagrip®-Impfstoff durch Injektion in das Muskelgewebe erhalten, ist mit einer Immunisierung gegen Tetanus und Grippe zu rechnen. Bei einzelnen Probanden / Patienten und bei Probanden bzw. Patienten, die Tetagrip®-Impfstoff auf die Haut erhalten, ist das Ausbleiben einer Immunantwort gegen Grippe und Tetanus möglich. Die vorgestellte klinische Studie wird von September 2005 bis Dezember 2005 durchgeführt. Sollte sich zeigen, dass durch die Haut geimpfte Probanden / Patienten keinen oder keinen ausreichenden Schutz gegen Grippe erworben haben, werden die Betroffenen umgehend informiert. Sie haben dadurch die Möglichkeit, sich bei entsprechendem Wunsch noch vor Beginn der Grippesaison 2005/2006 auf konventionelle Weise, d.h. per Injektion in den Oberarm, impfen zu lassen.
Ebenso besteht ggf. die Möglichkeit, dass HIV-infizierte Patienten unabhängig auf die Applikationsweise des Impfstoffs nicht auf die Grippeschutzimpfung ansprechen.
Die vorgestellte Studie wird wertvolle Erkenntnisse für zukünftige klinische Studien auf dem Gebiet der transkutanen Impfstoffapplikation und für die Entwicklung von Impfstoffen gegen HIV liefern.

AUSGLEICHSZAHLUNG IM FALL EINER VERLETZUNG

Eine Versicherungspolice für klinische Studien, die alle diese Studie betreffenden Schäden, die im Verlauf dieser Untersuchung auftreten, wurde durch den Sponsor, die Stiftung ORVACS, „Objetif Recherche Vaccin Sida“, abgeschlossen.

Während der Dauer der klinischen Studie, sollte außer in Notfallsituationen jede weitere medikamentöse Behandlung nur mit Einverständnis des Studienarztes durchgeführt werden. Sollten Sie eine mit der Studie in Verbindung stehende Schädigung Ihrer Gesundheit vermuten, sind Sie aufgefordert, umgehend die Versicherungsgesellschaft zu informieren. Sie können entweder den Versicherer selbst informieren oder, um es Ihnen zu erleichtern, kontaktieren Sie Ihren Studienarzt, er / sie wird dann in Ihrem Namen umgehend die Versicherungsgesellschaft informieren. Sie sollten von Ihrem Studienarzt eine Kopie dieses Berichtes erhalten.

DIE FREIWILLIGE NATUR DER TEILNAHME

Ihre Teilnahme an dieser klinischen Studie ist komplett freiwillig. Sie können jederzeit ablehnen, daran teilzunehmen und ebenso ist es Ihnen jederzeit möglich, auch wenn Sie heute zustimmen, Ihre Zustimmung zu jedem Zeitpunkt ohne Angabe von Gründen zurückzuziehen.

Wenn Sie sich entscheiden, nicht an dieser Studie teilzunehmen oder sich zurückzuziehen, wird das weder einen Einfluss auf Ihre zukünftige Behandlung haben noch Ihr Verhältnis mit Ihrem behandelnden Arzt beeinflussen. Sollten Sie diese Studie frühzeitig aus eigener Entscheidung abbrechen oder Aus einem anderen Grund ausschneiden (siehe Abschnitt frühzeitiges Beenden der klinischen Studie) ist es zu Ihrer eigenen Sicherheit notwendig, eine Abschlussuntersuchung vornehmen zu lassen.

Wenn Sie an dieser Studie teilnehmen, dürfen Sie mindestens einen Monat nach Ende dieser Studie an keiner neuen Studie teilnehmen.

ERLAUBNIS ZUR EINSICHT IN STUDIENUNTERLAGEN UND VERTRAULICHKEIT DER DATEN

Um das Monitoring dieser klinischen Studie sicher zu stellen, ist es notwendig, autorisierten Vertretern (Personen werden durch den vom Sponsor verantwortlichen Arzt, Prof. Dr. Christine Katlama festgelegt) in ihrer Eigenschaft als klinische Studienmonitore und Auditore den Zugang zu medizinischen Daten und den Ergebnissen dieser klinischen Studien zu ermöglichen.

Das gleiche gilt für örtliche und auswärtige Aufsichtsbehörden, die klinische Studien kontrollieren.

Inspektoren der örtlichen Behörden (regionale Ämter, Gesundheitsämter) und Bundesinstitute (Paul-Ehrlich-Institut (Bundesamt für Sera und Impfstoffe), europäische verantwortliche Stellen (EMEA, European Medicine Agency) und Mitglieder der Ethikkommission der Charité –

Universitätsmedizin Berlin Campus Mitte, die für Sie verantwortlich sind insofern als sie Rechtsgewalt gegenüber dem untersuchenden Arzt haben.

Ihre ausdrückliche schriftliche Zustimmung ist jedoch hierfür notwendig, für den Fall, dass dies notwendig sein sollte. Indem Sie diese Probanden / Patienten-Einverständniserklärung unterschreiben, erlauben Sie diesen Zugang.

Andere Informationen, die während dieser Studie erhoben werden, unterliegen der Geheimhaltung. Nur Ihr Studienarzt, der durch die ärztliche Schweigepflicht gebunden ist, wird Ihre Identität kennen und nur er / sie könne entsprechende Daten Ihrer Person zuordnen, da er / sie eine Liste der Probanden / Patienten hat, die zu den Probanden / Patienten-Nummern gehören.

Medizinische Daten, die während des Verlaufs der Studie gewonnen werden, werden nur unter strengster Wahrung des Datenschutzgesetzes anonym weitergegeben (d.h. ohne Ihre Identität preiszugeben) und vertraulich gegenüber den Vertretern des Sponsors gehalten; Studienberichte sowie mögliche entstehende Publikationen werden auch nur in einer anonymisierten Form auf die Daten der Probanden / Patienten Bezug nehmen.

Die für die Durchsicht und Analyse der Daten verantwortlichen Personen sind strengstens an die Verschwiegenheitsauflagen gebunden und unterliegen den strengen Anforderungen des Datenschutzgesetzes.

NEUE INFORMATION

Während der Dauer der klinischen Studie wird der Studienarzt Ihnen jede neue Information, die für Sie von Bedeutung ist, zur Verfügung stellen oder die ihm / ihr in Bezug auf die klinische Studie bekannt geworden ist. Unter diesen Voraussetzungen ist es Ihnen dann jederzeit möglich, Ihre Entscheidung bezüglich der Teilnahme an dieser klinischen Studie zu überprüfen.

VORZEITIGE BEENDIGUNG DER KLINISCHEN STUDIE

Unter bestimmten Umständen ist es auch möglich, dass der Studienarzt oder der Sponsor dieser klinischen Studie entscheidet, dass Ihre Teilnahme an dieser klinischen Studie ohne ihre vorherige Zustimmung vorzeitig beendet werden sollte oder abgebrochen wird. Dies könnte notwendig werden, weil Sie nicht in der Lage sind, die Anforderungen der klinischen Studie zu erfüllen oder im Falle dass Sie eine schwerwiegende Erkrankung entwickeln.

KONTAKTPERSONEN IM FALLE VON FRAGEN

Der Studienarzt und sein/ihre Kollegen werden Ihnen jederzeit zur Verfügung stehen, um weitere Fragen, die Sie im Zusammenhang mit dieser klinischen Studie haben, zu beantworten. Er / sie wird jederzeit bereit sein, Ihnen Fragen über Ihre Rechte als freiwilliger Proband / Patient in dieser klinischen Studie zu beantworten.

Vielen Dank für Ihre Mitarbeit.

Mit freundlichen Grüßen,

Name der Kontaktperson: Telefonnummer

____________________________ ____________________________

Studienarzt

EINVERSTÄNDNISERKLÄRUNG
ZUR TEILNAHME AN DER

KLINISCHEN STUDIE ZUR BEWERTUNG DER SICHERHEIT UND DER WIRKSAMKEIT DER APPLIKATION VON 0,5 ML TETAGRIP®-IMPFSTOFF (IMPFSTOFF GEGEN TETANUS UND GRIPPE) AUF DIE HAUT IM VERGLEICH ZUR INTRAMUSKULÄREN INJEKTION IN DEN OBERARM, IN INSGESAMT VIER GRUPPEN:
ZWEI GRUPPEN MIT JE 12 GESUNDEN PROBANDEN UND
ZWEI GRUPPEN MIT JE 12 HIV-INFIZIERTEN PATIENTEN
KLINISCHE STUDIE: MANON 05 – CUTAVAC

Proband / Patient:______________________
Familienname, Vorname

Probanden / Patienten-Nr.:______________________

Mit meiner Unterschrift auf diesem Formular

- **bestätige ich, dass ___________________________ (Studienarzt) mich ausführlich über die Bedeutung, die Notwendigkeit und den Ablauf dieser klinischen Studie unterrichtet hat.**
- **Ich bestätige hiermit, dass ich diese Information verstanden habe. Ich habe eine Zusammenfassung erhalten und hatte ausreichend Zeit zur Verfügung, Fragen zu stellen. Alle Fragen wurden zu meiner vollsten Zufriedenheit beantwortet, und ich hatte genügend Zeit, meine Entscheidung zu fällen.**
- **Ich stimme daher zu, an dieser klinischen Studie teilzunehmen.**

Meine Teilnahme ist mein freier Entschluss und erfolgt ausschließlich aus freiem Willen. Ich kann mich jederzeit ohne Angabe von Gründen aus der Studie

zurückziehen ohne dass dadurch meine weitere medizinische Betreuung oder zukünftige medizinische Behandlung beeinflusst werden. In einem solchen Fall würde ich jederzeit umgehend den Studienarzt informieren.

Ich bin darüber informiert, dass der Studienarzt zu jedem Zeitpunkt die Studie abbrechen kann.

Ich bin auch darüber informiert, dass diese Studie keinen unmittelbaren Vorteil für meine Gesundheit bringt.

Hiermit bestätige ich, dass ich z. Z. an keiner anderen klinischen Studie teilnehme und dass ich auch nicht in der Ausschlussperiode aufgrund einer vorhergehenden Studie bin.

Ich bin damit einverstanden, dass ich an keiner anderen klinischen Studie für die Dauer dieser Studie teilnehmen und auch einen Monat im Anschluss an das Ende dieser Studie an keiner weiteren Studie teilnehmen werde.

Ich stimme weiterhin zu, dass zum Zwecke des Monitoring dieser klinischen Studie der Vertreter des Sponsors in seiner Eigenschaft als klinischer Studienmonitor und Auditor Zugang zu dieser Einverständniserklärung und zu meinen medizinischen Unterlagen haben darf. Die gleiche Erlaubnis gebe ich auch den Vertretern der staatlichen Gesundheitsbehörden, die für die Überwachung von klinischen Studien verantwortlich sind und auch den Mitgliedern der Ethikkommission. Ich gebe meine Erlaubnis, dass diese Daten in anonymisierter Form für eine Computeranalyse durch den Sponsor oder durch einen Vertreter des Sponsors benutzt werden können.

Ich habe eine Kopie des Probanden / Patient-Informationsblattes und der Einverständniserklärung erhalten und werde sie während der gesamten Studiendauer aufbewahren. Das Original verbleibt beim Studienarzt in meinen Unterlagen.

Mit meiner Unterschrift unter dieses Formular erkläre ich mich zur Teilnahme an dieser klinischen Studie bereit, deren Ziel darin besteht die Sicherheit und die Wirksamkeit der Anwen

dung von kommerziell erhältlichem 0,5ml Tetagrip®-Impfstoff auf der Haut zu untersuchen und deren Wirksamkeit mit der der üblichen intramuskulären Impfung mit 0,5 ml Tetagrip®-Impfstoff zu vergleichen.

______________________________	________________________
Unterschrift des Probanden / Patienten	Ort und Datum
______________________________	________________________
Unterschrift des für die Aufklärung verantwortlichen Arztes	Ort und Datum

PATIENT INFORMED CONSENT FOR THE FRENCH CENTER

Objectif Recherche Vaccins SIDA
Hôpital Pitié-salpêtrière
47-83, boulevard de l'hôpital
75651 paris

MANON 05 – CUTAVAC

ESSAI MULTICENTRIQUE, INTERNATIONAL DE PHASE I RANDOMISE, OUVERT, COMPARATIF, EVALUANT ET COMPARANT LA SECURITE ET L'IMMUNOGENICITE D'UN MODE TRANSCUTANE D'ADMINISTRATION D'UN VACCIN AUTORISE CONTRE LE TETANOS ET LA GRIPPE, A LA VOIE CONVENTIONNELLE D'ADMINISTRATION INTRAMUSCULAIRE CHEZ LES VOLONTAIRES SAINS (COHORTE I) ET LES PATIENTS INFECTES PAR LE VIH (COHORTE II)

Cohorte II : patients infectés par le vih

Lettre d'information au patient

Monsieur,

Vous êtes suivi pour votre séropositivité par votre médecin qui vous propose de participer à cet essai clinique de vaccination.
Cette étude sera mise en œuvre à l'hôpital de jour du Service des Maladies Infectieuses de l'hôpital Pitié-Salpêtrière, sous la responsabilité du Pr. Christine KATLAMA.
Avant que toute procédure concernant cette étude clinique ne puisse commencer, il est indispensable que vous donniez votre accord écrit.
Veuillez lire soigneusement l'information suivante, pour vous préparer à l'entretien avec votre médecin. N'hésitez en aucun cas à poser toutes les questions qui vous paraissent importantes.

Vous ne signerez la déclaration d'accord que si l'importance et les détails pratiques de cette étude vous sont compréhensibles, si vous êtes d'accord pour y participer et si les droits et obligations vous incombant en tant qu'individu participant à cette étude vous paraissent clairs.

Le protocole d'étude et tous les documents relevants ont été présentés au CCPPRB (Commission Consultatif pour la Protection de Personnes se Prêtant à la Recherche Biomédicale) de l'hôpital Pitié-Salpêtrière, composé de membres indépendants sans intérêt direct avec cette étude.

POURQUOI CETTE ETUDE ?

Les traitements antirétroviraux ont permis une nette amélioration de la maladie VIH conduisant à une réduction importante de la charge virale et à une augmentation du nombre des lymphocytes CD4. Cependant, ces traitements nécessitent une utilisation au long cours en induisant souvent des effets secondaires toxiques. Une nouvelle stratégie est actuellement explorée par les médecins et les chercheurs : il s'agit d'utiliser des vaccins pour stimuler les défenses immunitaires spécifiques de l'organisme chez des personnes déjà infectées par le VIH, afin d'alléger les stratégies thérapeutiques. De recherches restent cependant nécessaires pour améliorer l'efficacité des vaccins.

QU'EST CE QUE L'ETUDE MANON 05 ?

La vaccination, est généralement faite par injection dans les tissus musculaires ou sous-cutanés. Ces tissus sont pauvres en cellules immunes nécessaires à une réponse vaccinale pleinement efficace. Le développement de procédures de vaccination bien tolérées et à faible risque ciblant mieux le système immun et capables d'induire une protection améliorée pourrait améliorer l'efficacité des vaccinations contre des maladies pour lesquelles on ne peut pas jusqu'ici vacciner avec succès comme par exemple la tuberculose, l'hépatites et le VIH.

L'étude Manon 05 teste une nouvelle voie d'administration des vaccins, à travers la peau. En effet la peau, en contact constant avec les agents pathogènes, est une barrière importante contre les agressions. Sa richesse en cellules immunes, appelées cellules de Langerhans, lui permet d'induire des défenses immunitaires efficaces contre ces agressions. La mise au point d'une nouvelle procédure de vaccination utilisant cette fonction naturelle de la peau et permettant d'introduire le vaccin à proximité de ces cellules immunes cutanées, semble être une voie très prometteuse d'optimisation des vaccinations. Cependant la peau est composée de plusieurs couches (couche cornée, épiderme, derme, hypoderme) et différentes études indiquent que les substances appliquées sur la peau pénètrent par les follicules pileux qui représentent des interruptions naturelles importantes de la couche cornée et sont riches en ces cellules immunes nécessaires au succès de la vaccination. Atteindre ces cellules immunes nécessite l'ouverture des follicules pileux. Différentes études chez des modèles animaux et même chez l'homme ont déjà montré que des vaccinations sont possibles à travers la peau, mais aucune n'a cherché à optimiser cette voie en ciblant les follicules pileux.

L'objectif de l'étude Manon-05 est de tester une nouvelle voie de pénétration optimale d'un vaccin dans la peau via les follicules pileux. Pour cela la méthode la plus efficace à faire pénétrer des substances appliquées sur la peau est la méthode dite du « Cyanacrylat-Stripping » Cette technique consiste à appliquer sur la peau une couche de colle de cyanacrylate (super-glue) sous un film adhésif ; le retrait de ce film adhésif élimine les cellules mortes et autres débris des couches supérieures de la peau (soit environ seulement 30% de la couche cornée totale). La peau en soi reste intacte. Cette méthode est couramment utilisée en cosmétologie afin d'étudier la pénétration des cosmétiques *et est acceptée par les agences européennes et américaines d'évaluation des médicaments*

Ainsi l'objectif principal de l'étude MANON 05 – CUTAVAC est d'évaluer la sécurité et l'immunogenicité de l'administration sur la peau de 0,5 ml d'un vaccin approuvé et commercialisé en France contre le Tétanos et la grippe Tetagrip®, par rapport à l'injection intramusculaire habituelle, chez 24 volontaires sains et chez 24 patients infectés par le VIH, répartis dans quatre sous-groupes avec 12 sujets chacun.

QUEL VACCIN ?

Le vaccin utilisé dans cette étude est Tetagrip®, un vaccin agréé, fabriqué par l'entreprise Sanofi Pasteur, et couramment utilisé en France chez l'adulte pour la prévention conjointe du tétanos et de la grippe. Il permet, à l'occasion de la vaccination annuelle contre la grippe, de pratiquer la vaccination de rappel contre le tétanos, ou s'intègre dans le schéma de première vaccination contre le tétanos. Une injection unique d'une dose de 0,5 ml du vaccin, par voie intramusculaire ou sous-cutanée, suffit à conférer l'immunité protectrice vis-à-vis de la grippe et l'immunité de rappel efficace contre le tétanos.

DESCRIPTION DE L'ETUDE MANON 05

- **Principaux critères d'inclusion**

L'étude sera effectuée sur deux groupes de volontaires :

- Cohorte I : 24 volontaires sains
- Cohorte II : 24 volontaires séropositifs pour le VIH

Tous les sujets entrant dans l'étude devront être du sexe masculin, âgés entre 18 et 45 ans et ne pas avoir été vaccinés contre le tétanos depuis quatre ans et contre la grippe depuis un an. Pour la Cohorte II les patients devront être séropositifs pour le VIH, recevoir un traitement antirétroviral efficace depuis au moins 6 mois, avoir un taux de CD4 supérieur à 350/mm^3 depuis au moins un an et une charge virale inférieure à 400 copies/ml depuis au moins 6 mois.

- **DEROULEMENT DE L'ETUDE**

L'étude compare 2 voies d'administration du vaccin Tetagrip® de façon identique à l'intérieur des 2 Cohortes subdivisées en 2 **groupes**.

Les sujets seront répartis **après tirage au sort** dans un des groupes suivants :

- **Groupe A : Mode d'administration sur la peau**
- **Groupe B : Mode Intramusculaire conventionnel**

Les sujets en nombre de 12 dans chaque groupe recevront une administration unique de 0.5 ml de Tetagrip® dans le muscle deltoïde (partie extérieure du bras gauche).

I. Groupe A : Mode d'administration sur la peau

Les sujets du groupe A recevront le vaccin selon la voie d'administration sujette de l'étude, à la dose unique de 0,5 ml de Tetagrip® distribuée sur deux secteurs de peau de 4x 4 cm chacun, sur l'extérieur du bras supérieur gauche (muscle deltoïde). La procédure est indiquée plus bas.

II. Groupe B : Mode intramusculaire conventionnel

Les sujets du groupe B recevant l'injection intramusculaire conventionnelle deviennent les témoins de l'étude, c'est-à-dire qu'ils vont recevoir la dose de 0,5 ml Tetagrip® dans un seul site par la voie conventionnelle intramusculaire, dans le muscle deltoïde (partie extérieure du bras gauche). C'est une procédure agréée et couramment utilisée. Il faut en conséquence s'attendre à une immunisation sûre contre la grippe dans ce groupe.

- **MISE EN OEUVRE DE L'ETUDE**

La mise en oeuvre de l'étude est prévue d'octobre 2005 à décembre 2005. Les sujets participants vaccinés par la peau seront informés immédiatement s'ils ont acquis une protection suffisante contre la grippe ou pas. Ils auront ainsi la possibilité, en cas d'inefficacité de cette voie de vaccination, de se faire vacciner de manière conventionnelle au début de la saison de grippe 2005/2006.

- Le nombre de patients de la Cohorte II participant à cette étude sera de 24 (12 patients par groupe). Ils seront recrutés dans deux centres, l'un en France et l'autre en Allemagne.
- La durée totale de l'essai est de 3 mois.

- La durée d'étude par patient s'élève à 5 semaines (1 semaine de pré-inclusion et 4 semaines de visites de contrôles et suivi. En tant que participant à cette étude il vous sera demandé de venir à l'hôpital huit (8) jours non consécutifs, répartis comme suit :

Jour – 7 : Visite de Pré-Sélection et Pré-Inclusion
Jour 0 : Inclusion dans l'étude et Procédure d'application du vaccin de l'étude
Jour 1, 3, 7,14, 21 : Visites de suivi
Jour 28 : Fin de l'étude

- ***JOUR - 7 : PRE-SELECTION ET* PRE-INCLUSION :**

a. Présentation et Signature du consentement éclairé,
b. Entretien avec votre médecin, étude du dossier médical y compris des vaccinations précédentes ainsi que la médication actuelle,

CE N'EST QU'APRES VERIFICATION ECRITE DE TOUS LES CRITERES D'INCLUSION ET D'EXCLUSION QUE VOUS SEREZ DEFINITIVEMENT INCLUS DANS L'ETUDE.

- Attribution du code d'identification et assignation du mode d'application du vaccin Tetagrip®

- ***JOUR 0 :* APPLICATION DU VACCIN TETAGRIP®**

a. Prélèvement de 75 ml de sang pour les tests de réponses immunes au vaccin ainsi que pour mesurer votre charge virale et votre taux des CD4,
b. Mesure de qualités physiologiques de votre peau (pH, perte d'eau par la peau, production de sueur, teneur en eau de la couche cornée),
c. Application du vaccin Tetagrip® selon le tirage à sort dans un des deux groupes de l'étude :

LE JOUR 0, SELON LE GROUPE ASSIGNE, VOUS DEVREZ PREVOIR ENVIRON 2 ½ HEURES POUR L'APPLICATION DU VACCIN TETAGRIP® SUR LA PEAU (GROUPE A) OU ENVIRON 30-45 MINUTES POUR L'APPLICATION INTRAMUSCULAIRE (GROUPE B).

Groupe A : Application sur la peau

a. Délimitation de deux secteurs de la peau de 4x 4cm sur la partie extérieure du bras supérieur gauche et Rasage
b. Préparation de la peau par la méthode du « Cyanacrylat-Stripping » : Une couche mince de colle est appliquée sur chacune des 2 sections de la peau et couverte d'un film adhésif qui est enlevé après un temps de séchage d'à peu près 20 minutes.
c. Application de 0,25 ml du vaccin Tetagrip® sur chaque site délimité.
d. Couverture d'un « patch » hydrocolloidal pour 24 heures.

***GROUPE B : INJECTION INTRAMUSCULAIRE* :**

a. Désinfection du site d'injection à l'extérieur du bras supérieur gauche
b. Injection intramusculaire de 0,5 ml du vaccin Tetagrip®.

Visites de contrôle: Toutes les sujets participant à l'essai se soumettrant au total de 5 contrôles sur une période de quatre semaines. Le premier contrôle a lieu le jour 1.

- ***Jour 1***

a. Ablation du « patch »
b. Examen du site d'application et des ganglions lymphatiques axillaires,
c. Interrogatoire sur la tolérance cutanée et générale de la procédure de vaccination.
d. Mesure de qualités physiologiques de votre peau (pH, perte d'eau par la peau, production de sueur, teneur en eau de la couche cornée.

- ***Jour 3, jour 7, jour 14, jour 21***

a. Examen du site d'application, inspection des ganglions lymphatiques axillaires,
b. *Question concernant des signes d'incompatibilité, question concernant les modifications du traitement complémentaire*,
c. Prélèvement de 40 ml (l'équivalent de 3 cuillères à soupe) de sang aux jours 7 et 14,
d. Mesure de qualités physiologiques de votre peau (pH, perte d'eau par la peau, production de sueur, teneur en eau de la couche cornée) le jour 7 et le jour 14 Jour 28.

- ***Jour 28***

a. Examen physique
b. *Examen du site d'application, inspection des ganglions lymphatiques axillaires,*
c. *Question concernant des signes d'incompatibilité, questions concernant des modifications du traitement complémentaire*,
d. Prélèvement de 75 ml (équivalent à 5 cuillères à soupe) de sang
e. Mesure de qualités physiologiques de votre peau (pH, perte d'eau par la peau, production de sueur, teneur en eau de la couche cornée),

La vaccination avec Tetagrip® est en général très bien tolérée. Des réactions locales au site d'injection ainsi que des réactions générales peuvent cependant se manifester, par exemple, des douleurs intestinales ou de l'estomac ainsi que des épisodes de fièvre transitoire sont possibles. Cependant une réaction d'hypersensibilité est possible contre les composants du vaccin, ou des composants de la colle ou du *pansement*. Les sujets avec hypersensibilité connue contre ces composants ne sont pas acceptés dans l'étude. Une inspection est effectuée sur le site d'application aux jours 1, 7, 14, 21 et 28 après la vaccination. Un gonflement temporaire des ganglions lymphatiques locaux dans l'épaule peut apparaître. Le sujet est examiné aussi à cet égard à chaque visite de suivi.
Pendant la durée de l'étude les prélèvments représenteront un total de 230 ml de sang pour les analyses immunologiques, ainsi que pour mesurer votre charge virale et taux de CD4.

➢ **INSTRUCTIONS SPECIALES**

- Dans les 24 heures après l'application du vaccin, toutes les activités induisant la production de sueur doivent être évitées dont toute activité physique, séances de sauna, etc.
- Pendant la durée totale d'étude tout sport intensif est interdit.
- Outre les produits de nettoyage habituels (gel douche et/ou du savon), vous ne pourrez appliquer sur la surface du bras où le vaccin a été appliqué aucun autre produit, et ceci pour toute la durée de l'étude.
- De plus, vous ne pourrez pas utiliser des traitements locaux ou par voie systémique pouvant influencer les résultats de l'étude comme par exemple, les médicaments qui influencent la fonction du système immun (le médecin de l'étude examinera cela avec vous, mais tout sujet utilisant ces médicaments est exclus de l'étude).
- Outre les cas urgents, vous ne devrez pas commencer de nouveaux traitements médicamenteux pendant votre participation à l'étude. Si cela est nécessaire, veuillez informer votre médecin d'étude le plus immédiatement possible, au mieux avant que vous ne commenciez avec la nouvelle médication.
- Les irradiations solaires doivent être évitées sur les portions de peau traitées. Les séances d'irradiation UV (solarium) doivent être suspendues pour la durée d'étude.

Les sujets inclus dans l'essai recevront un agenda qui résume tous les guides d'étude. Tous les événements médicaux devraient être enregistrés dans cet agenda. Veuillez apporter cet agenda à chaque contrôle

➢ PARTICIPATION

Si vous ne désirez pas prendre part à cette étude ou si vous souhaitez vous en retirer à quelque moment et pour quel que motif que ce soit, vous continuerez à bénéficier du suivi médical et cela n'affectera en rien votre surveillance future. Pour interrompre votre participation il suffira d'en avertir le médecin responsable. De la même manière, votre médecin a le droit d'interrompre votre participation à l'étude s'il pense que c'est bénéfique pour vous.

➢ RISQUES ET BENEFICES DE L'ETUDE

Les risques théoriques sont liés au vaccin. Comme après toute injection vaccinale, vous pouvez ressentir une gêne au point d'injection : douleur, rougeur, gonflement ou induration ainsi que des signes généraux, fièvre, frissons, fatigue, maux de tête ou éruption cutanée. Ces effets secondaires habituels pour des vaccins ne nécessitent généralement aucun traitement spécifique et disparaissent spontanément.

Le bénéfice escompté de cette vaccination est acquérir une immunité vis-à-vis de la grippe et une immunité protectrice de rappel contre le tétanos. La prévention de la grippe est particulièrement recommandée chez les sujets qui présentent un déficit immunitaire avec risque élevé de complications associées. De même le tétanos est une affection grave dont le seul traitement repose sur la prévention.

➢ LES CONTRAINTES LIEES A L'ETUDE

Elles sont liées au respect du calendrier de consultation et bilans sanguins (prélèvements de 40 à 75 ml de sang soit un volume total de 230 ml sur 5 semaines) pour les patients inclus. Une visite de pré-inclusion, puis 7 visites dont 1 visite correspondant à l'application du vaccin par l'une ou l'autre méthode de vaccination selon les groupes. Des bilans immuno-virologiques complets seront pratiqués à J0, et J28.

- **CONFIDENTIALITE**

Toutes les données recueillies resteront strictement confidentielles. Votre nom et votre adresse ne seront connus que de votre médecin. La partie de votre dossier médical concernant l'essai ne pourra être consultée que par les personnes qui collaborent à la recherche, déléguées par l'ORVACS qui est promoteur de cette étude, le médecin ou éventuellement des représentants des autorités sanitaires ou des services d'inspection locaux ou étrangers qui contrôlent les essais cliniques.

- **NATURE DES DONNEES INFORMATISEES**

Compte tenu de la nécessité de la recherche et de son analyse ultérieure, les données recueillies vous concernant feront l'objet d'un traitement informatisé et anonyme. La loi « Informatique et Liberté » (article 40), du 6 janvier 1978, modifiée par la loi du 6 août 2004 ; prévoit votre droit d'opposition à l'informatisation de ces données ainsi que votre droit d'accès à ses informations et votre droit de rectification de celles-ci pendant toute la durée de l'essai.

Ces droits pourront s'exercer auprès du Docteur :…………………………………………………………….

Les informations qui feront l'objet de ce traitement informatisé sont les suivantes :

- Données démographiques
- Evènements cliniques observés
- Résultats biologiques, immunologiques et virologiques
- Traitements concomitants et prophylactiques prescrits.

De plus, comme le prévoit l'article L.1122-1 du Code de la Santé Publique, vous pourrez être tenu informé(e), si vous le souhaitez, des résultats globaux de cette étude, une fois que toutes les données auront été validées et analysées.

- **DE NOUVELLES INFORMATIONS**

Si pendant la durée de l'étude il s'avérait que de nouvelles informations concernant l'étude clinique et/ou qui pourraient affecter votre participation à l'étude sont connues par le sponsor, votre médecin d'étude vous communiquera ces informations. Dans ces conditions il vous est possible de réexaminer votre décision concernant la participation à cette étude clinique à tout moment.

- **INFORMATIONS COMPLEMENTAIRES**

- Assurance : L'ORVACS est le promoteur de cette étude. En tant que promoteur ORVACS a souscrit un contrat de responsabilité civile conformément aux dispositions de l'article L. 1121-7 du code de Santé Publique.
- Interruption prématurée de l'étude: Il est aussi possible que votre médecin, le médecin de l'étude ou le promoteur de cette étude clinique; décide que votre participation à cette étude clinique doit être interrompue. Cela parce que vous n'êtes pas en mesure de remplir les exigences de l'étude clinique, par exemple parce que vous développez une maladie sérieuse.

- Si vous participez à cette étude, vous ne pouvez pas participer à une autre étude clinique au moins un mois après la fin de cette étude.
- La personne à contacter dans le cas où vous auriez des questions sera votre médecin responsable de l'étude. Lui et ses collègues seront disponibles à tout moment, pour répondre aux questions que vous pourriez avoir en rapport avec cette étude clinique. Ils seront à tout moment prêts à vous répondre sur vos droits en tant que participant dans cette étude clinique.

Objectif Recherche Vaccins SIDA
Hôpital Pitié-salpêtrière
47-83, boulevard de l'hôpital
75651 paris

MANON 05 – CUTAVAC

ESSAI MULTICENTRIQUE, INTERNATIONAL DE PHASE I RANDOMISE, COMPARATIF, EVALUANT ET COMPARANT LA SECURITE ET L'IMMUNOGENICITE D'UN MODE TRANSCUTANE D'ADMINISTRATION D'UN VACCIN AUTORISE CONTRE LE TETANOS ET LA GRIPPE, A LA VOIE CONVENTIONNELLE D'ADMINISTRATION INTRAMUSCULAIRE CHEZ LES VOLONTAIRES SAINS (COHORTE I) ET LES PATIENTS INFECTES PAR LE VIH (COHORTE II)

COHORTE II : PATIENTS INFECTES PAR LE VIH

FORMULAIRE DE CONSENTEMENT ECLAIRE

Nom : ________________________ **Prénom :** ___________________________

Date de naissance :________________________

Le Dr m'a proposé de participer à la recherche nommée ci-dessus et m'en a expliqué les objectifs. Je comprends les contraintes et les avantages liés à ma participation. Je connais la possibilité qui m'est réservée d'interrompre ma participation à tout moment sans avoir à justifier ma décision, mais je m'engage à en informer le Dr Cela ne remettra pas en cause la qualité des soins qui me seront dispensés ultérieurement.

J'accepte que les données enregistrées à l'occasion de ce protocole, y compris compte tenu des nécessités de la recherche, mes origines ethniques et mes mœurs, puissent faire l'objet d'un traitement informatisé. J'ai bien noté que le droit d'accès prévu par la loi « Informatiques et Libertés » (article 40), s'exerce à tout moment auprès du Dr et que je pourrai exercer mon droit de rectification et d'opposition auprès de celui-ci.

Je confirme par ceci, que je ne participe pas actuellement à d'autre étude clinique et que je ne suis pas dans la période d'exclusion d'une étude précédente. Je m'engage à ne pas participer dans une autre étude clinique pendant ma participation à MANON 05–CUTAVAC, ainsi que dans le mois suivant la fin de cette étude.

J'accepte que tout médecin ou scientifique impliqué dans le déroulement de cet essai, ainsi que le représentant des autorités de santé aient accès aux données qui me concernent dans le respect le plus strict de la confidentialité.

Mon consentement ne décharge en rien le promoteur de l'étude de ces responsabilités, je conserve tous mes droits garantis par la loi.

J'accepte librement de participer à cette recherche dans les conditions précisées dans la note d'information qui m'a été remise.

Fait à Paris le, **Signature du patient :**

Je soussigné(e) Dr , certifie avoir communiqué à , toute information utile concernant les objectifs et les modalités de cette recherche. Je m'engage à faire respecter les termes de ce formulaire de consentement conciliant le respect des droits et des libertés individuelles et les exigences d'un travail scientifique.

Fait à Paris le, **Signature de l'investigateur :**

APPENDIX II

DECLARATION OF HELSINKI

DECLARATION OF HELSINKI

Ethical Principles For Medical Research Involving Human Subjects

Adopted by the 18th WMA General Assembly Helsinki, Finland, June 1964 and amended by the
29th WMA General Assembly, Tokyo, Japan, October 1975
35th WMA General Assembly, Venice, Italy, October 1983
41st WMA General Assembly, Hong Kong, September 1989
48th WMA General Assembly, Somerset West, Republic of South Africa, October 1996
and the 2nd WMA General Assembly, Edinburgh, Scotland, October 2000
Note of Clarification on Paragraph 29 added by the WMA General Assembly, Washington 2002

A. INTRODUCTION

1. The World Medical Association has developed the Declaration of Helsinki as a statement of ethical principles to provide guidance to physicians and other participants in medical research involving human subjects**.** Medical research involving human subjects includes research on identifiable human material or identifiable data.

2. It is the duty of the physician to promote and safeguard the health of the people. The physician's knowledge and conscience are dedicated to the fulfillment of this duty.

3. The Declaration of Geneva of the World Medical Association binds the physician with the words, "The health of my patient will be my first consideration," and the International Code of Medical Ethics declares that, "A physician shall act only in the patient's interest when providing medical care which might have the effect of weakening the physical and mental condition of the patient."

4. Medical progress is based on research which ultimately must rest in part on experimentation involving human subjects.

5. In medical research on human subjects, considerations related to the well-being of the human subject should take precedence over the interests of science and society.

6. The primary purpose of medical research involving human subjects is to improve prophylactic, diagnostic and therapeutic procedures and the understanding of the aetiology and pathogenesis of disease. Even the best proven prophylactic, diagnostic, and therapeutic methods must continuously be challenged through research for their effectiveness, efficiency, accessibility and quality.

7. In current medical practice and in medical research, most prophylactic, diagnostic and therapeutic procedures involve risks and burdens.

8. Medical research is subject to ethical standards that promote respect for all human beings and protect their health and rights. Some research populations are vulnerable and need special protection. The particular needs of the economically and medically disadvantaged must be recognized. Special attention is also required for those who cannot give or refuse consent for themselves, for those who may be subject to giving consent under duress, for those who will not benefit personally from the research and for those for whom the research is combined with care.

9. Research Investigators should be aware of the ethical, legal and regulatory requirements for research on human subjects in their own countries as well as applicable international requirements. No national ethical, legal or regulatory requirement should be allowed to reduce or eliminate any of the protections for human subjects set forth in this Declaration.

B. BASIC PRINCIPLES FOR ALL MEDICAL RESEARCH

10. It is the duty of the physician in medical research to protect the life, health, privacy, and dignity of the human subject.

11. Medical research involving human subjects must conform to generally accepted scientific principles, be based on a thorough knowledge of the scientific literature, other relevant sources of information, and on adequate laboratory and, where appropriate, animal experimentation.

12. Appropriate caution must be exercised in the conduct of research which may affect the environment, and the welfare of animals used for research must be respected.

13. The design and performance of each experimental procedure involving human subjects should be clearly formulated in an experimental protocol. This protocol should be submitted for consideration, comment, guidance, and where appropriate, approval to a specially appointed ethical review committee, which must be independent of the investigator, the sponsor or any other kind of undue influence. This independent committee should be in conformity with the laws and regulations of the country in which the research experiment is performed. The committee has the right to monitor ongoing trials. The researcher has the obligation to provide monitoring information to the committee, especially any serious adverse events. The researcher should also submit to the committee, for review, information regarding funding, sponsors, institutional affiliations, other potential conflicts of interest and incentives for subjects.

14. The research protocol should always contain a statement of the ethical considerations involved and should indicate that there is compliance with the principles enunciated in this Declaration.

15. Medical research involving human subjects should be conducted only by scientifically qualified persons and under the supervision of a clinically competent medical person.
The responsibility for the human subject must always rest with a medically qualified
person and never rest on the subject of the research, even though the subject has given consent.

16. Every medical research project involving human subjects should be preceded by careful assessment of predictable risks and burdens in comparison with foreseeable benefits to the subject or to others. This does not preclude the participation of healthy volunteers in medical research. The design of all studies should be publicly available.

17. Physicians should abstain from engaging in research projects involving human subjects unless they are confident that the risks involved have been adequately assessed and can be satisfactorily managed. Physicians should cease any investigation if the risks are found to outweigh the potential benefits or if there is conclusive proof of positive and beneficial results.

18. Medical research involving human subjects should only be conducted if the importance of the objective outweighs the inherent risks and burdens to the subject. This is especially important when the human subjects are healthy volunteers.
19. Medical research is only justified if there is a reasonable likelihood that the populations in which the research is carried out stand to benefit from the results of the research.

20. The subjects must be volunteers and informed participants in the research project.

21. The right of research subjects to safeguard their integrity must always be respected.

Every precaution should be taken to respect the privacy of the subject, the confidentiality of the patient's information and to minimize the impact of the study on the subject's physical and mental integrity and on the personality of the subject.

22. In any research on human beings, each potential subject must be adequately informed of the aims, methods, sources of funding, any possible conflicts of interest, institutional affiliations of the researcher, the anticipated benefits and potential risks of the study and the discomfort it may entail. The subject should be informed of the right to abstain from participation in the study or to withdraw consent to participate at any time without reprisal. After ensuring that the subject has understood the information, the physician should then obtain the subject's freely-given informed consent, preferably in writing. If the consent cannot be obtained in writing, the non-written consent must be formally documented and witnessed.

23. When obtaining informed consent for the research project the physician should be particularly cautious if the subject is in a dependent relationship with the physician or may consent under duress. In that case the informed consent should be obtained by a well-informed physician who is not engaged in the investigation and who is completely independent of this relationship.

24. For a research subject who is legally incompetent, physically or mentally incapable of giving consent or is a legally incompetent minor, the investigator must obtain informed consent from the legally authorized representative in accordance with applicable law.
These groups should not be included in research unless the research is necessary to promote the health of the population represented and this research cannot instead be performed on legally competent persons.

25. When a subject deemed legally incompetent, such as a minor child, is able to give assent to decisions about participation in research, the investigator must obtain that assent in addition to the consent of the legally authorized representative.

26. Research on individuals from whom it is not possible to obtain consent, including proxy or advance consent, should be done only if the physical/mental condition that prevents obtaining informed consent is a necessary characteristic of the research population. The specific reasons for involving research subjects with a condition that renders them unable to give informed consent should be stated in the experimental protocol for consideration and approval of the review committee. The protocol should state that consent to remain in the research should be obtained as soon as possible from the individual or a legally authorized surrogate.

27. Both authors and publishers have ethical obligations. In publication of the results of research, the investigators are obliged to preserve the accuracy of the results. Negative as well as positive results should be published or otherwise publicly available. Sources of funding, institutional affiliations and any possible conflicts of interest should be declared in the publication. Reports of experimentation not in accordance with the principles laid down in this Declaration should not be accepted for publication.

C. ADDITIONAL PRINCIPLES FOR MEDICAL RESEARCH COMBINED WITH MEDICAL CARE

28. The physician may combine medical research with medical care, only to the extent that the research is justified by its potential prophylactic, diagnostic or therapeutic value.

When medical research is combined with medical care, additional standards apply to protect the patients who are research subjects.

29. The benefits, risks, burdens and effectiveness of a new method should be tested against those of the best current prophylactic, diagnostic, and therapeutic methods. This does not exclude the use of placebo, or no treatment, in studies where no proven prophylactic, diagnostic or therapeutic method exists. *(See footnote*)*

30. At the conclusion of the study, every patient entered into the study should be assured of access to the best proven prophylactic, diagnostic and therapeutic methods identified by the study.

31. The physician should fully inform the patient which aspects of the care are related to the research. The refusal of a patient to participate in a study must never interfere with the patient-physician relationship.

32. In the treatment of a patient, where proven prophylactic, diagnostic and therapeutic methods do not exist or have been ineffective, the physician, with informed consent from the patient, must be free to use unproven or new prophylactic, diagnostic and therapeutic measures, if in the physician's judgement it offers hope of saving life, reestablishing health or alleviating suffering. Where possible, these measures should be made the object of research, designed to evaluate their safety and efficacy. In all cases, new information should be recorded and, where appropriate, published. The other relevant guidelines of this Declaration should be followed.

FOOTNOTE:

Note of Clarification on Paragraph 29 of the WMA Declaration of Helsinki

The WMA hereby reaffirms its position that extreme care must be taken in making use of a placebocontrolled trial and that in general this methodology should only be used in the absence of existing proven therapy. However, a placebo-controlled trial may be ethically acceptable, even if proven therapy is available, under the following circumstances:
- Where for compelling and scientifically sound methodological reasons its use is necessary to determine the efficacy or safety of a prophylactic, diagnostic or therapeutic method; or
- Where a prophylactic, diagnostic or therapeutic method is being investigated for a minor condition and the patients who receive placebo will not be subject to any additional risk of serious or irreversible harm.

All other provisions of the Declaration of Helsinki must be adhered to, especially the need for appropriate ethical and scientific review.

17.C
The Declaration of Helsinki (Document 17.C) is an official policy document of the World Medical Association, the global representative body for physicians. It was first adopted in 1964 (Helsinki, Finland) and revised in 1975 (Tokyo, Japan), 1983 (Venice, Italy), 1989 (Hong Kong), 1996 (Somerset-West, South Africa) and 2000 (Edinburgh, Scotland). Note of clarification on Paragraph 29 added by the WMA General Assembly, Washington 2002.

. . .
6.10.2002

APPENDIX III

INSURANCE CERTIFICATE

APPENDIX IV

SKIN PHYSIOLOGY MEASUREMENTS

MANON 05 - CUTAVAC

**Sponsor Representative:**	Christine KATLAMA, MD
**Clinical Study Coordinator**:	Ulrike BLUME-PEYTAVI, MD PhD
**Immunology Study**: **Coordinator:**	Brigitte AUTRAN MD, PhD
**Project Leader:**	Norma WINCKER, PhD
**Sponsor:**	ORVACS

SOP Skin Physiological Parameters measurements

**Autor:**	**Dr. A. Vogt**	Date: 20.07.05	Signature
**Co-Authors:**			
**Approved by:**	**Prof. Dr. U. Blume-Peytavi**	Date:22.07.2005	Signature

Index

1. **Scientific Background**
2. **Methods**
3. **References**
4. **Enclosures**

1. Scientific Background

Transcutaneous vaccination strategies aim to target antigen-presenting cells of the skin in their natural environment, and various studies have shown that transcutaneously applied vaccine preparations induce specific and robust immune responses. The aim of this study ís to assess the immune response against the transcutaneously applied test vaccine Tetagrip® (0.5 ml) compared to conventional i.m. injection of 0.5 ml Tetagrip® vaccine.
The condition of the skin, e.g. the hydration state, the production of sebum, etc. is an important factor for the percutaneous penetration of topically applied compounds (Lotte 1987, Lademann 2001, Schaefer 2001). The measurement of the skin physiological parameters TEWL, SCH, Skin pH and sebum production is therefore included in the study.

2. Methods

Transepidermal water loss (TEWL), stratum corneum hydration, skin pH and sebum production will be assessed using a Multi Probe Adapter MPA® (Courage-Khazaka, Cologne, Germany). We use a portable system which consists of the different probes, the Multi-Probe-Adapter and a laptop which is connected to the device. The measurements will be performed in the Clinical Research Center for Hair and Skin Physiology, Department of Dermatology, Charité- Universitätsmedizin Berlin or in the HIV Treatment & Research Unit, Department of Internal Medicine II, University Frankfurt am Main, respectively. Room-temperature and air humidity will be documented. All skin measurements will be performed according to the manufacturer’s recommendation (see Appendix) after an adjustment period of 30 minutes. The measurements will be performed on Day 0, Day 1, Day 7, Day 14 and Day 28 of the study.
All the skin measurements performed in this study are based on non-invasive methods. The results can be obtained within seconds by placing the probes on the skin surface.

The following probes will be used on the Multi Probe Adapter:

Tewameter® TM 300

The Tewameter is used to measure the rate of water loss through the skin. Since the skin barrier regulates the rate of water loss from the body, the rate of transepidermal water loss is a measure of the condition of the skin barrier. When skin is damaged, its barrier function is impaired resulting in high water loss.

The Corneometer® CM 825

The corneometer measures the amount of moisture in the outer layer of the skin (skin hydration) and the ability of the skin to retain moisture (skin moisture capacity
The measurement of the skin moisture is based on the internationally recognized Corneometer®-method (a capacitance method).

The Skin-pH-Meter® PH 905

The skin pH will also be measured directly on the skin surface. This is possible because the excretions of the skin are almost an aqueous solution.

Sebumeter® SM 815

The sebumeter method is based on a photometric method (grease spot photometer) which allows to directly measure the sebum secretion of the skin.

3. References

Lademann J, Otberg N, Richter H, Weigmann HJ, Lindemann U, Schaefer H, Sterry W. Investigation of follicular penetration of topically applied substances. Skin Pharmacol Appl Skin Physiol 14 Suppl 1:17-22, 2001

Lotte C, Rougier A, Wilson DR, Maibach HI. In vivo relationship between transepidermal water loss and percutaneous penetration of some organic compounds in man: effect of anatomic site. Arch Dermatol Res. 1987;279(5):351-6

Schaefer H, Lademann J. The role of follicular penetration. A differential view. Skin Pharmacol Appl Skin Physiol 14 (Suppl 1):23-27, 2001

4. Enclosures

MPA General Information®

APPENDIX V

TRANSCUTANEOUS APPLICATION OF TETAGRIP® VACCINE

MANON 05 - CUTAVAC

Sponsor Representative: Christine KATLAMA MD

Clinical Study Coordinator: Ulrike BLUME-PEYTAVI, MD PhD

Immunology Study: **Coordinator:** Brigitte AUTRAN MD, PhD

Project Leader: Norma WINCKER, PhD

Sponsor: ORVACS

SOP Epicutaneous application of Tetagrip® Vaccine

**Autor:**	**Dr. A. Vogt**	Date: 20.07.05	Signature
**Co-Authors:**	**Dr. G. Schäfer**<br>**S. Hadam**	Date:<br>20.07.2005<br>Date:	
**Approved by:**	**Prof. Dr. U. Blume-Peytavi**	Date:20.07.2005	Signature

Index

1. **Material**
2. **Scientific Background**
3. **Methods**
4. **References**

1. Material

- Permanent Skin Marker *(Skin Marker H7003, Falc)*
- Plastic template *(2 Squares 4 x4 cm)*
- Disposable Shaver *(Disposable Hospital Razor, Art-Nr.: 182 H, Wilkinson Sword GmbH, Solingen, Germany)*
- Cyanacrylate Glue *(Superglue, UHU GmbH & Co. KG, Buehl/Baden, Germany)*
- Adhesive Tape *(Nr. 57176-00000, 66m x 50mm, tesa® Beiersdorf,Beiersdorf,Germany)*
- Sheet of Paper *(21x7cm)*
- Rubber Roll
- Window-Colour-Konturpaste
  (Nr. 4469/ko,Max-Bringmann GmbH&Co.,Wendelstein, Germany)
- Tetagrip® Vaccine *(Sanofi, France)*
- Glove *(care & serve®)*
- Comfeel Plus *(Comfeel® Plus Transparent 9x 14cm Art.-Nr.: 3542, Coloplast A/S, Denmark)*

2. Scientific Background

This study aims to assess the immune response against the epicutaneously applied test vaccine Tetagrip® (0.5 ml) compared to conventional i.m. injection of 0.5 ml Tetagrip® vaccine. Tetagrip® vaccine is approved and commercially available in France, where it is routinely used for vaccination against tetanus and influenza. Vaccination against influenza is recommended for individuals > 60 years of age, individuals who suffer from chronic dieases and for medical personal only, which means that a large population is influenza-naive. Tetagrip® vaccine therefore represents a safe and approved test vaccine for these proof of concept investigations. Kenney et al. recently demonstrated that the intradermal administration of only one fifth of the standard i.m. dose of an influenza vaccine elicited immunogenicity that was similar or better than that elicited by common i.m. injection (Kenney 2004). We therefore hypothesize that the epicutaneous application of Tetagrip® vaccine in the commercially available standard preparation of 0.5 ml should be sufficient to induce an immune response.

3. Methods

General information

This SOP applies to volunteers who receive Tetagrip® vaccine transcutaneously. The application will be performed at room temperature. Prior to the application the volunteer will be seated in the test room for 30 minutes to adjust to the conditions. During the incubation periods between the different steps of application the volunteer remains seated. He will have the possibility to watch TV/Video. After the application of Tetagrip® vaccine, the volunteer will be instructed not to take a shower or bath and to avoid any activity which causes sweating or mechanical stress to the investigational site, e.g. physical exercise, during the following 24 hours.

1. Positioning of the arm

The left arm will be abducted to 90° and positioned horizontally on a plastic pad with the external part of the upper arm facing upwards. The arm will be kept in this position for the whole duration of the application. It may only be lowered during the drying period after the application of cyanacrylate glue (see “4. CSSS”).

2. Demarcation of the investigational site

Two squares of 4 x 4 cm will be delimitated on the external part of the left upper arm using a plastic template. Adhesive tape strips will be used to tape the template on the skin. The investigational sites will be delimitated using a permanent skin marker (skin marker H7003, Falc).

3. Shaving

The hair will be shaved on the investigational sites as well as on the surrounding skin (2 cm on the top and the bottom of each investigational site and 1 cm on both sides) using a dry razor (Disposable Hospital Razor, Art-Nr.: 182 H, Wilkinson Sword GmbH, Solingen, Germany).

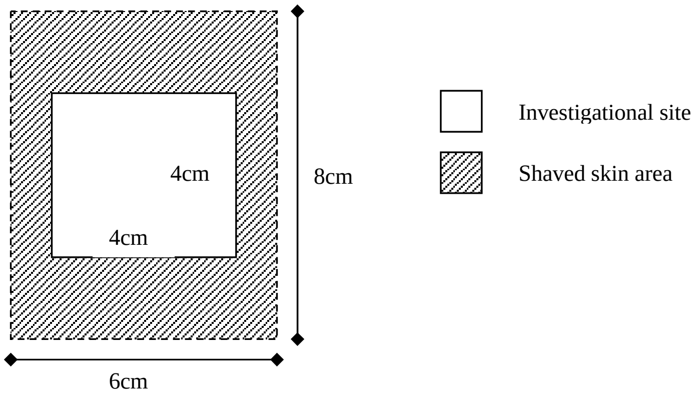

Fig. 1: The two investigational sites of 4x4 cm will be deliminated on the external portion of the upper left arm. To avoid binding of topically applied vaccine to hair

fibres and to avoid pain during the following cyanacrylate skin surface stripping, the investigational site and the surrounding skin will be shaved.

Cyanacrylate Skin Surface Stripping (CSSS)
After the shaving, cyanacrylate glue (superglue, UHU GmbH & Co. KG, Buehl/Baden, Germany) will be applied drop-wise (9 drops per investigational site = ca. 190 mg) on each investigational site. The edge of a glass microscope slide will be used to spread the glue evenly on the investigational sites. Immediately after the spreading, adhesive tape (6x5 cm, Nr. 57176-00000, 66m x 50mm, Tesa® Beiersdorf, Beiersdorf, Germany) will be placed on the investigational site. A sheet of paper (21x7cm) will be placed on top of the tape and a rubber roll will be rolled uniformly 10 times across the investigational sites to spread the glue evenly on the skin surface and to eliminate air bubbles.
During this waiting period, the left arm may be lowered. The volunteer remains seated. After 20 minutes, the arm will be repositioned as described in “1. Positioning of the arm”, the tape and the adhering glue will be removed quickly from the skin surface.

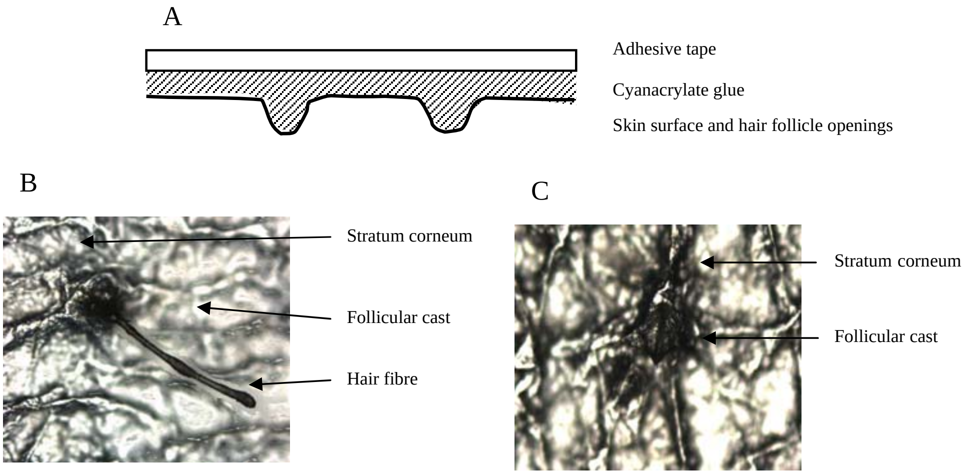

Fig.2: Cyanacrylate Skin Surface Stripping (CSSS) removes keratinized material, lipids and other cell debris from the follicular openings and approx. 30% of the stratum corneum. Occasionally hair fibres, especially vellus hairs, are removed as well (Mills 1983). The remaining stratum corneum and the viable epidermis are left intact. This technique is routinely used in our laboratory and also by other groups to improve the percutaneous penetration of topically applied compounds and to determine the amount of substance which enters the follicular reservoir (Hueber 1994, Schaefer 2001).
After spreading of the cyanacrylate glue on the skin surface, adhesive tape is placed on top of the glue layer. When the glue is hardened, removal of the adhesive tape also removes the glue, the adherent stratum corneum and follicular casts (A-C). In our preliminary studies, one CSSS on the upper arm of human volunteers removed the follicular casts of all hair follicles and approx. 10% of the hair fibres.

4. Demarcation of the investigational site by a silicone barrier

After the CSSS, the investigational sites will be deliminated with a silicone barrier (Window-Colour-Konturpaste, Nr. 4469/ko,Max-Bringmann GmbH&Co.,Wendelstein, Germany) to prevent spreading of the subsequently applied vaccine. The silicone barrier has to dry for 20 minutes. During this incubation time, the volunteer remains seated and the left arm remains in its position (see “1 Positioning of the Arm”).

5. Application of Tetagrip® vaccine

250 µl of Tetagrip® vaccine are applied drop-wise from the original syringe as provided by the manufacturer onto the skin surface of each investigational site (16 drops per investigational site, each drop approx. 16 µl). A soft massage will be applied by moving a gloved finger tip (care & serve®) presaturated with Tetagrip® vaccine for ca. 1 minute in circles on the investigational site to spread the vaccine evenly on the skin surface and to improve the percutaneous penetration (Genina 2002, McElnay 1993). This procedure is followed by an incubation time of 20 minutes.

6. Application of Comfeel bandage

At the end of the application procedure a hydrocolloid bandage (Comfeel® Plus Transparent 9x 14cm Art.-Nr.: 3542, Coloplast A/S, Denmark) will be applied to protect the investigational sites and to provide occlusion which may further enhance the percutaneous penetration of Tetagrip® vaccine. The bandage remains on the skin for 24 hrs. It will be removed by the investigator on Day 1, 24 hrs after the application of Tetagrip® vaccine on Day 0. The volunteers will be instructed not to take a shower or bath and to avoid any activity which causes sweating or mechanical stress to the investigational site, e.g. physical exercise, during these 24 hours.

References

Genina EA, Bashkatov AN, Sinichkin YP, Kochubey VI, Lakodina NA, Attshuler GB, Tuchin W (2002) I vitro and in vivo study of dye diffusion into the human skin and hair follicles. *J Biomed Opt* **7**: 471-477.

Hueber F, Schaefer H, Wepierre J (1994) Role of transepidermal and transfollicular routes in percutaneous absorption of steroids: In vitro studies on human skin. *Skin Pharmacol* **7**: 237-244.

Kenney RT, Frech SA, Muenz LR, Villar CP, Glenn GM (2004) Dose sparing with intradermal injection of influenza vaccine. *N Engl J Med* 351: 2295-2301.

McElnay LC, Benson HA, Harland R, Hadgraft J (1993) Phonophoresis of methylnicotinate: A preliminary study to elucidate the mechanism of action. *Pharm Res* **10**: 1726-1731.

Mills OH Jr, Kligman AM (1983) The follicular biopsy. *Dermatologica* **167**: 57-63.

Otberg N, Richter H, Schaefer H, Blume-Peytavi U, Sterry W, Lademann J (2003) Variations of hair follicle size and distribution in different body sites. *J Invest Dermatol* **122**:14-19.

APPENDIX VI

BLOOD COLLECTION AND SHIPMENT

MANON 05 - CUTAVAC

Sponsor Representative: Christine KATLAMA MD

Clinical Study Coordinator: Ulrike BLUME-PEYTAVI, MD PhD

Immunology Study: **Coordinator:** Brigitte AUTRAN MD, PhD

Project Leader: Norma WINCKER, PhD

Sponsor: ORVACS

SOP Blood collection and shipment

**Author:**	**Dr. N.Wincker**	Date: 29.07.05	Signature
**Co-Authors:**	**Cécile Gameiro**	Date: 29.07.05	Signature
**Approved by:**	**Prof. Dr. Brigitte Autran**	Date: 05.08.05	Signature

Index

1. Material
2. Methods
3. Appendices

1. Material

- ACD tubes
- Dry tubes
- Labels

2. Methods

The purpose of this procedure is to ensure the adequate collection, shipment and retrieval of blood

Before all manipulations, please pay attention, wear gloves and lab coats.

Blood samples transportation to the immunology laboratory in Paris will be done at room temperature.

Immunological determinations on fresh blood samples need to be done within 24 h maximum after drawn, therefore it is mandatory that blood samples transportation time does not exceed 22h h.

2.1 Blood Collection

Four blood samples (10 ml/tube) are planned in the protocol for immunological determinations at the following time points : D–7, D0, D7, D14 and D28.

Blood samples should be obtained as : 1 dry tube
3 ACD tubes (10ml)

All tubes should be identified with patient code before blood withdrawn Blood samples should be maintained at room temperature while waiting for shipment.

2.2 Blood samples shipment

Blood samples should be shipped to the immunology laboratory in Paris.

The shipment will be carried out by the « World Courier » (WC) company located in Germany following instructions were given them by ORVACS

At the scheduled times (D–7, D0, D7, D14 and D28) the fresh blood samples will be picked-up by a representative of WC. It is strongly recommended to request the samples pick-up by the transporter only once the scheduled patient is on site. This is with the aim of avoiding additional fees in case the patient could not come to have the visit.

To request the pick-up of samples, the investigator will call to WC – Germany to the following phone number 0049 30 243 14 xxx, by e-mail to jstreit@worldcourier.de or tglatzer@worldcourier.de, or by fax to 0049 30 243 14 219.A preprinted fax form is included below for each participating center.

Fresh blood samples will be shipped as diagnostic specimens packed with *IATA* packing instruction 650. The packaging PI650 will be supplied by WC.

A form “list of contents” (see below) should be filled at each shipment. In this form the consignee address as well as the nature of the samples will be indicated.

Note : To guarantee that all immunological determinations will be done within 24 hours after drawn, it is strongly recommended to schedule blood withdrawal of volunteers/patients between 10 and 14 hours, Monday through Thursday.

The contact address at ORVACS is :

Contact name:	Norma WINCKER
Telephone :	00 33 1 44 24 23 98
Fax:	00 33 1 44 24 17 96
E-mail	norma.wincker@psl.ap-hop-paris.fr

The contact address at World Courier Germany is :

tglatzer@worldcourier.de

3. Appendices

World Courier France
30, rue Mozart
92110 Clichy La Garenne
Tél.: 01 55 21 20 00
Fax: 01 55 21 20 15

PICK UP REQUEST

Can be sent by e-mail to: jstreit@worldcourier.de + tglatzer@worldcourier.de + nbeschke@worldcourier.de or by fax to : 0049 30 243 14 219

Client : **ORVACS** | Account number : **5647**

Quotation number: FF 06 28 2005-01 | **Study reference:** MANON 05 CUTAVAC

Date and time of pick up: ……. / ……. /2005 between ……. H …….

Date and time of delivery : Within 22 hours

Draw time :

No pick up or delivery allowed on week ends or days off.

1/ Nature of samples: **Plasma Blood Cells DNA Other (specify)……**

2/ Samples are from: Humans Animals (type of animal):

3/ Is the shipment containing dangerous goods Yes No

4/ **Temperature of transport**: Ambient Frozen -80°C (dry ice)

Frozen -160°C (nitrogen) Refrigerated (between+2°C & +8°C)

5/ Packaging PI650 supplied by: World Courier Shipper – Type & dimensions (cm):

6/ **Packaging**: Number of vials: ………………… Quantity per vial (ml): ……………..

**Shipper :**	**Charité Universitätsmedizin** Berlin	**Consignee:**	Laboratoire d'Immunologie Cellulaire et Tissulaire
Building / Floor:	Clinical research center for hair and skin physiology	Building / Floor:	Bâtiment CERVI
Street:	Dept of dermatology Schumannstrasse 20-21	Street:	83 boulevard de l'Hôpital
Postcode / City:	10117 BERLIN	Postcode / City:	75013 PARIS
Country:	GERMANY	Country:	FRANCE
Contact name :	Ulrike BLUME	Contact name:	Cécile GAMEIRO
Telephone :	0049 30 450 518 122	Telephone :	0033 1 42 17 74 81
E-mail :	Annika.vogt@charite.de	E-mail :	cecile.gameiro@psl.ap-hop-paris.fr

Name of the person who is responsible of this shipment and who will receive the proof of delivery

Contact name: Norma Wincker	Telephone : 0033 44 24 23 98
Fax: 0033 44 24 17 96	e-mail :norma.winckerpsl.ap-hop-paris.fr

World Courier France
30, rue Mozart
92110 Clichy La Garenne
Tél.: 01 55 21 20 00
Fax: 01 55 21 20 15

PICK UP REQUEST

Can be sent by e-mail to: jstreit@worldcourier.de + tglatzer@worldcourier.de + nbeschke@worldcourier.de or by fax to : 0049 30 243 14 219

Client : **ORVACS**	Account number : **5647**
**Quotation number:** FF 06 28 2005-01	**Study reference:** MANON 05 CUTAVAC
Date and time of pick up:	……. / ……. /2005 between ……. H …….
Date and time of delivery :	Within 22 hours
Draw time :	
**No pick up or delivery allowed on week ends or days off.**	

1/ Nature of samples: **Plasma Blood Cells DNA Other (specify)……**

2/ Samples are from: Humans Animals (type of animal):

3/ Is the shipment containing dangerous goods Yes No

4/ **Temperature of transport**: Ambient Frozen -80°C (dry ice)
Frozen -160°C (nitrogen) Refrigerated (between+2°C & +8°C)

5/ Packaging PI650 supplied by: World Courier Shipper – Type & dimensions (cm):

6/ **Packaging**:
Number of vials: ………………… Quantity per vial (ml): ……………..

**Shipper :**	**Klinikum Johann Wolfgang Goethe** Universitat	**Consignee:**	Laboratoire d'Immunologie Cellulaire et Tissulaire
Building / Floor:	HIV treatment and research unit, Internal medicine 2	Building / Floor:	Bâtiment CERVI
Street:	Theodor Stern Kai 7	Street:	83 boulevard de l'Hôpital
Postcode / City:	60590 FRANKFURT AM MAIN	Postcode / City:	75013 PARIS
Country:	GERMANY	Country:	FRANCE
Contact name :	Schlomo Staszweski<br>Stephan Merten	Contact name:	Cécile GAMEIRO
Telephone :	00 49 69 63 01 46 88 / 57 12	Telephone :	0033 1 42 17 74 81
E-mail :		E-mail :	cecile.gameiro@psl.ap-hop-paris.fr

Name of the person who is responsible of this shipment and who will receive the proof of delivery	
Contact name: Norma Wincker	Telephone : 0033 44 24 23 98
Fax: 0033 44 24 17 96	e-mail :norma.winckerpsl.ap-hop-paris.fr

Charité Universitätsmedizin Berlin
Clinical research center for hair and skin physiology
Dept of dermatology and allergy
Schumannstrasse 20-21
10117 BERLIN
GERMANY
Contact name: Ulrike BLUME/Annika VOGT
Telephone : 0049 30 450 518 122

LIST OF CONTENTS / LISTE DETAILLEE DU CONTENU

Echantillons diagnostiques emballés d'après l'instruction d'emballage 650
Diagnostic specimens packed in compliance with IATA packing instruction 650

Nous confirmons que les échantillons sont à destination de :
We confirm that samples will be delivered to

ADRESSE DU DESTINATAIRE / CONSIGNEE'S ADDRESS

Laboratoire d'immunologie cellulaire et tissulaire
INSERM U543
Hôpital Pitié-Salpêtrière

83, boulevard de l'hôpital, Bâtiment CERVI – 4ème étage
75651 paris Cedex 13

Nature des échantillons/*Nature of samples* :

☐ Sérum / *Serum*	.... vials x	☐mL	☐mg	Total Quantity : ....
☐ Plasma / *Plasma*	.... vials x	☐mL	☐mg	Total Quantity : ....
☐ Sang / *Blood*	.... vials x	☐mL	☐mg	Total Quantity : ....
☐ Urine / *Urine*	.... vials x	☐mL	☐mg	Total Quantity : ....
☐ Autre / *Other :*	.... vials x	☐mL	☐mg	Total Quantity : ....

Nom/Titre & Signature Date :
Name/Title & Signature Date :

Hospital of the Johann Wolfgang Goethe University Frankfurt

HIV treatment and research unit

Internal medicine 2

Theodor Stern Kai 7

60590 FRANKFURT AM MAIN

GERMANY

Contact name: Schlomo Staszewski/Stefan Merten
Telephone : 00 49 69 63 01 46 88

LIST OF CONTENTS / LISTE DETAILLEE DU CONTENU

Echantillons diagnostiques emballés d'après l'instruction d'emballage 650

Diagnostic specimens packed in compliance with IATA packing instruction 650

Nous confirmons que les échantillons sont à destination de :
We confirm that samples will be delivered to

ADRESSE DU DESTINATAIRE / CONSIGNEE'S ADDRESS

Laboratoire d'immunologie cellulaire et tissulaire
INSERM U543
Hôpital Pitié-Salpêtrière

83, boulevard de l'hôpital, ***Bâtiment CERVI – 4ème étage***
75651 paris Cedex 13

Nature des échantillons/*Nature of samples* :

☐ Sérum / *Serum*	.... vials x	☐mL	☐mg	Total Quantity : ....
☐ Plasma / *Plasma*	.... vials x	☐mL	☐mg	Total Quantity : ....
☐ Sang / *Blood*	.... vials x	☐mL	☐mg	Total Quantity : ....
☐ Urine / *Urine*	.... vials x	☐mL	☐mg	Total Quantity : ....
☐ Autre / *Other :*	.... vials x	☐mL	☐mg	Total Quantity : ....

Nom/Titre & Signature Date :
Name/Title & Signature Date :

APPENDIX VII

IMMUNOLOGICAL ANALYSIS OF THE IMMUNE RESPONSE AGAINST THE TETAGRIP® VACCINE

MANON 05 - CUTAVAC

Sponsor Representative: Christine KATLAMA MD

Clinical Study Coordinator: Ulrike BLUME-PEYTAVI, MD PhD

Immunology Study: **Coordinator:** Brigitte AUTRAN MD, PhD

Project Leader: Norma WINCKER, PhD

Sponsor: ORVACS

SOP Immunological analysis of the immune responses against Tetagrip® vaccine

**Author:**	**Sophie Hilpert**	Date: 29.07.05	Signature
**Co-Authors:**	**Cécile Gameiro**<br>**Dr. N.Wincker**	Date: 29.07.05	Signature
**Approved by:**	**Prof. Dr. Brigitte Autran**	Date: 05.08.05	Signature

Index

1. Material
2. Methods for specific immunology
   A. Serum storage for specific antibody measurement
   B. Specific Antibody measurement
   C. Peripheral Blood Mononuclear Cell (PBMC) separation.
   D. Quantification of PBMC producing IFNγ

3. References

1. MATERIAL

Specific marerials are specified for each procedure before technique explanation.

2. METHODS FOR SPECIFIC IMMUNOLOGY

This study will be performed in the core laboratory (Laboratoire d'Immunologie Cellulaire – Paris) using standard operating procedure.

A. SERUM STORAGE FOR SPECIFIC ANTIBODY MEASUREMENT

Ten ml of blood collected in a dry tube will be spun, 10 minutes at 2 000rpm, to obtain at least 2 ml of serum. Make 4 aliquots each containing 1 ml of serum, and store immediatly at - 80 °C.

B. SPECIFIC ANTIBODY MEASUREMENT

Antibodies will be measured by specific sub-contractors with expertise in each field

- Antibodies against Influenza will be measured at the Centre National de Référence de la Grippe - I. Pasteur, according to a standard operating procedure for evaluation of titers of neutralizing antibodies (inhibition of haemaglutination).
- Antibodies against Tetanus Toxoïd will be measured by ELISA at the Laboratoire de Microbiologie, Hôpital Henri Mondor.

C. PERIPHERAL BLOOD MONONUCLEAR CELL (PBMC) SEPARATION.

- PBMC will be isolated from fresh blood collected in ACD tubes, after centrifugation over a Ficoll-Hypaque gradient at 2200rpm for 30 minutes and washed twice in PBS before use.

D. QUANTIFICATION OF PBMC PRODUCING IFNg

D-1. HUMAN IFNγ ELISPOT TECHNIQUE

a. Intended Use

The ELISPOT assay is designed to enumerate cytokine producing CD8 and/or CD4 T cells in a single cell suspension. This method has the advantage of requiring a minimum of in-vitro manipulations allowing cytokine production analysis as close as possible to in-vivo conditions. This technique is designed to determine the frequency of cytokine producing cells under a given stimulation, and the follow-up of such frequency during a treatment and/or a pathological state.

b. Principle of the method

After cell stimulation, locally produced cytokines are captured by a specific monoclonal antibody. After cell removal, trapped cytokine molecules are revealed by a secondary biotinylated detection antibody, which is in turn recognized by

streptavidin conjugated to alkaline phosphatase. PVDF-bottomed-well plates are then incubated with NBT/BCIP substrate. Colored "purple" spots indicate cytokine production by individual cells.

A response is considered positive when the number of spots obtain after deduction of the background is above 50 spot/million of cells. It is feasible on frozen cells with a good reproducibility compared to fresh cells as shown in the Manon 01 study (to be published).

c. Materials

- 96 PVDF-bottomed-well plates. 5Millipore MultiScreen plates cat # MAIPS4510, Immobilon p membrane)
- Cell culture medium : RPMI 1640 and 2 mM glutamine, 1% sodium pyruvate, 1% Antibiotics (penicillin, streptomycin) and 10% FCS (Gibco, France)
- Tween 20. Store at room temperature.
- Streptavidin alkaline phosphatase conjugate (Amersham, RPN, 1234). Store at +4°C.
- Bovine serum albumin. Store at +4°C (Sigma)
- Capture antibody : anti-IFN-□ (Diaclone, IgG1, B-B1) (concentration 1mg/ml ready to use). Store at +4°C.
- Phosphate buffered saline (PBS) 1X (If stock solution in 10X concentration prepare PBS 1X
- for the whole procedure). Store at +4°C.
- Biotinylated detection antibody : anti IFN-g biotinylated (Diaclone, B-G1) (concentration 100 µg/ml). Store at +4°C.
- Buffer PBS + Tween 0,5 ml/l PBS (= 0,05%). Store at +4°C.
- Buffer PBS + BSA 5 g/l (= 0,5%). Store at +4°C.
- Buffer Tris pH = 9.5 = 0,9g NaCl (Sigma) in 100 ml distilled water and 1g $MgCl_2$ (Merck) and 1.21g Tris (Sigma). Store at +4°C.
- Dimethylformamide : DMF (Sigma). Store at room temperature.
- 4-Nitro-Bleu Tetrazolium Chloride (NBT, Sigma)
- 5-Bromo-4 Chloro-3- Indolyl Phosphate (BCIP, Sigma) 50 mg per 1 ml DMF. Store in aliquots at -20°C.
- PHA-p (Murex) : stock solution 1 mg/ml. Store in aliquots at -20°C.

d. Antigens

CD4 and CD8 T cell responses against tetanos and Influenza Neuraminidase will be evaluated in triplicate experiments with :

- Tetanus Toxoïd antigen
- Influenza Neuraminidase (Sigma)
- Tetagrip vaccine (Sanofi Pasteur)
- PHA from Murex
- All aliquots are stored at -20°C

Over all 18 wells per time point done for all time points and for all patients.

e. ELISPOT Procedure

- Wash wells three times with 100 µl of sterile PBS 1X.

- Dilute capture antibody at 1/1000 in PBS. Mix strongly and dispense 100 µl into each well, cover the plate and incubate 2 hours at 37°C in a CO2 incubator or overnight at +4°C (the plate can be prepared 5 days in advance).
- Wash wells three times with 100 µl of sterile PBS.
- Dispense 100 µl of culture medium into wells, cover and incubate for 30 minutes at 37°C. Empty wells by flicking the plate over a sink and tapping it on absorbent paper.
- Cell preparation : dilute PBMC in culture media at 106/ml, 105/ml

Do not wash wells.

- Dispense into wells 100 µl of sterile cell suspension containing the appropriate number of cells and adequate concentration of stimulator in triplicates. Leave one line with medium alone for negative control. Add the peptide in appropriate concentration or the mitogen (positive control: PHA-p = 0,5 µg/ml). Cover the plate with a standard 96-well plate plastic lid and incubate cells at 37°C in a CO2 (5%) incubator for 20 hours.

During this period do not agitate or move the plate.

- Wash wells three times with PBS (200 µl)
- Wash again three times with PBS-0,05% -Tween 20.
- Wash again 3 times with PBS.
- This washing procedure is better performed on an automated washer alternatively it could be done manually.
- Dilute reconstituted detection antibody at 1/500 into 10 ml of PBS-0,5% BSA. This step has to be prepared extemporaneously! Distribute 100 µl in wells, cover the plate and incubate 4 hours at 37°C, 5% C02.
- Wash wells three times with PBS.
- Dilute streptavidin-alkaline phosphatase conjugate at 1/1000 into PBS-0,5% BSA extemporaneously! Distribute 100 µl of the dilution in wells. Seal the plate and incubate for 1 hour at 37°C, 5% C02.
- After three washes with PBS, empty wells by repeated tapping on absorbent paper. At this stage it is important to remove all residual buffer.
- Prepare the dye (for one plate): 10 ml Buffer Tris + 45 µl NBT + 35 µl BCIP **(respect this order)** extemporaneously!
- Distribute 100 µl of NBT/BCIP buffer in wells.
- Let the reaction go for 10 min, **no longer that 15 min**, at room temperature. (Do not cover the plate)
- Rinse wells three times with distilled water.
- Dry wells. Read spots the day after, or store the plate at room temperature away from direct light until lecture which can be post when plates are kept in a dark dry atmosphere.
- Read out on dried plates either immediately after assay or after storage in dark and dry condition, on an automated ELISpot reader. A response is considered positive when the number of spots obtain after deduction of the background is above 50 spot/million of cells

D-2. ICC INTRA CELLULAR CYTOKINE CYTOMETRY DETECTION

a. Intended use

The nature of cell producing IFNγ upon specific antigen stimulation will be defined in a second step after results of ELISPOT on total PBMC have shown which antigens induce positive responses (above 50 SFC/million PBMC). It is chosen for practicability and accuracy of analysis to define the CD4 and CD8 cell origin by intra-cellular flow cytometry (ICC) IFNγ detection after stimulation with the positive antigen. This assay will be performed on cryopreserved cells.

b. Principle of the method

The ICC assay allows to quantify simultaneously CD4 and CD8 T cells producing IFNγ and provide results equivalent to those obtained with ELISPOT (Y. Sun, JIM, 2003) although the threshold of detection for ICC is higher: it detects ELISPOT

values above 500 SFC/million PBMC. Therefore, ICC will be performed as a second step for all antigens or pool of peptides inducing IFNγ in the first ELISPOT test. Each antigen will be tested individually if SFC values above 500 SFC/million PBMC.

c. Materials

- 96 conic well plates
- PBS-BSA 0.5%-Saponin 1%
- Cell culture medium (R+): RPMI 1640 and 2 mM glutamine, 1% sodium pyruvate, 1% Antibiotics (penicillin, streptomycin)
- Fetal calf serum
- BFA 10mg/ml, final concentration 5 µg/ml
- PBS-BSA 0.5%
- PBS-BSA 0.5%-Saponin 0.1%
- Paraformaldehyde 4% Ph 7.2-7.4
- *Monoclonal antibodies:*

CD8 APC	Becton-Dickinson	345775
CD3 PerCP Cy5.5	Becton-Dickinson	340949
IFNγ FITC	Becton-Dickinson	340449
CD69 PE (3/8/69)		
CD45/14		
Peptide X	1 mg/ml final 5 µg/ml	
PHA-P	1 mg/ml	

d. Antigens

CD4 and CD8 T cell response against Tetanos Toxoïd and Influenza will be evaluated in triplicate experiments.

- Tetanus Toxoïd antigen :
- Influenza Neuraminidase
- Tetagrip vaccine
- PHA from Murex

e. Procedure

- PBMC are isolated by Ficoll-Hypaque gradient centrifugation, washed in RPMI twice, and used to 10.10^6 cell/ml in RPMI 20% SVF.
- Distribute 100 µl of cell suspension to 10.10^6 cell/ml (i.e:1 million live PBMC per tube)
- Distribute 100 µl of peptides in each corresponding well.
- Incubate 2 hours at 37°C in 5 % CO_2
- Distribute 20 µl of BFA in each well
- Incubate for 4 hours at 37°C in 5 % CO_2

This preparation could be performed the same day as ICC, or the day before if the plate is stored overnight at 4°C.

- *Membrane staining:*

- Collect each well in haemolyse tube qsp 4 ml of PBS/BSA 0.5% at 4°C.
- Centrifuge 10 min at 1300 rpm
- Aspirate supernatant
- Add 10 µl anti-CD3 PerCP Cy5.5 and 10 µl anti-CD8 APC and 10 µl anti-CD69
- Incubate 30 min at 4°C and in the dark
- Wash with 2 ml of PBS/BSA 0.5% (centrifuge 5 min at 1300 rpm)
- Aspirate supernatant
- Add 200 µl of paraform-aldehyde 4%

- Resuspend the cells
- Incubate 15 minutes a room temperature
- Add with 2 ml of PBS/BSA 0.5%/Saponin 0.1%
- Centrifuge 5 min at 1300 rpm

Aspirate supernatant

Add 100 µl of PBS/BSA 0.5%/Saponin 0.1%

- *Intracellular staining*

- Add 10 µl of anti-IFNγ FITC
- Incubate 20 minutes at room temperature
- Wash with 2 ml PBS/BSA 0.5%/Saponin 0.1% (centrifuge 5 min at 1300 rpm)
- Aspirate supernatant
- Wash with 2 ml of PBS/BSA 0.5% (centrifuge 5 min at 1300 rpm)
- Resuspend in 300 µl of PBS/BSA 0.5%
- Run immediately with a minimum of 50 000 lymphocytes CD8+ CD3+ acquired and an optimum of 1 million acquired.

E. CRYOPRESERVATION OF CELLS AND THAWING PROCEDURES

Cryopreservation: The PBMCs will be frozen in 10 % of DMSO (Dimethylsulfoxide) and 90% of FCS (Fetal calf serum) in at least 4 vials containing a minimum of 10x 10^6PBMC, within 24 hours of blood harvest.
Cells will be prepared in a sterile way on ice then immediately placed in a freezing box (Nalgene cryo 1°C freezing container), previously placed at 4°C; then immediately at -80°C for 18 hours, then transferred to nitrogen liquid for storage. The viability of the cryopreserved cells should be above 80%.

Thawing procedure : Before thawing the cells, prepare a 15 ml tube (A) containing 10 ml of RPMI-50 % FCS. Put the vial in the heat bath until obtaining an ice pea. With 5 ml of medium of the tube A harvest the cells and rinse the vial in order to obtain the maximum of cells. Spin the tube 8 minutes at 1400 rpm, resuspend the pellet in 10 ml of RPMI-20% FCS. Spin again the tube 8 minutes at 1400 rpm, then resuspend the pellet in 4 ml of RPMI –5% FCS. Count the cells (% of alive with exclusion dye for dead cells and dead cells), and adjust at 1.106 million live cells/ml of medium. Leave the cells in a culture flask overnight in incubator.
The next morning, transfer the cells in a 15 ml tube, rinse the flask with a small volume of 20% FCS and transfer in the 15ml tube. Centrifuge the tube at 1400 rpm for 8 minutes and resuspend the pellet in 4 ml 20%-FCS. Measure the volume, count the cells and adjust the volume to obtain 1 million cells/ml.

APPENDIX VIII

RANDOMIZATION FORM

STUDY MANON 05-CUTAVAC **Page 1/1**

**|__|__|__|**	**|__|__||__|__|**	**Investigator's name**	**Baseline**
**Subjects's Initials**	**Subject N°**	**--------------------------------**	

RANDOMIZATION FORM

Please now register the volunteer/patient by using the randomization form and fax it with pages 1, 2, 3, 4, 5 of the screening visit to:

INSERM U 720

Fax: 01 42 16 42 61

Please wait for your patient number before Tetagrip® administration

Date: |__||__| |__||__| |__|__|__|__|

Center name and address:____

Phone number: ______________________ **Fax number:** _________________

Comments (if appropriate):____

Name of sender: ______________________ **Signature:** _________________

To be completed by the methodology/data management department representative responsible for the randomization of the subject:

Name of study manager: Norma Wincker

Name of the responsible for randomization: Tchadie BOMMENEL

The volunteer/patient respect all the criteria of eligibility and he is definitively included in the study.

Comments (if appropriate) : ____

Signature of study manager:_____________________________

Signature of randomization responsible: ________________________

The volunteer/patient will receive Tetagrip® vaccine:

MODE OF TETAGRIP® VACCINE ADMINISTRATION ALLOCATION

- ❑ **ARM A: TRANSCUTANEOUS MODE**
- ❑ **ARM B: INTRAMUSCULAR MODE**

The VOLUNTEER/PATIENT code is: |__|__|__| |__|__||__|__|

Subjects's Initials **Subject N°**

Date of Randomization |__||__| |__||__| |__|__|__|__|

APPENDIX IX

SEVERITY OF VACCINE REACTIONS

Severity of Vaccine Reactions

**Erythema** – abnormal redness of the skin		
None	0	No erythema
Mild	1	Slight pinkness present
Moderate	2	Definite redness, easily recognized
Severe	3	Intense redness
**Pruritus** - itching sensation		
None	0	No itching,
Mild	1	Slight itching, not really bothersome,
Moderate	2	Definite itching that is somewhat bothersome; without loss of sleep,
Severe	3	Intense itching that has caused pronounced discomfort; night rest interrupted. Excoriations of the skin from scratching may be present.

**Burning** – prickling pain sensation immediately after (within 5 minutes) dosing		
None	0	No stinging/burning
Mild	1	Slight warm, tingling/stinging sensation, not really bothersome
Moderate	2	Definite warm, tingling/stinging sensation that is somewhat bothersome,
Severe	3	Hot, tingling/stinging sensation that has caused definite discomfort.

**Desquamation** – abnormal shedding of the stratum corneum		
None	0	No scaling
Mild	1	Barely perceptible shedding, noticeable only on light scratching or rubbing,
Moderate	2	Obvious not profuse shedding,
Severe	3	Heavy scale production.

APPENDIX X

SERIOUS ADVERSE EVENTS GRADING TABLE

Appendix D: Grading of Adverse Events

APPENDIX D1: LABORATORY TEST ABNORMALITIESTABLE FOR GRADING SEVERITY OF ADVERSE EVENTS FOR DOSE MODIFICATION PURPOSES AND DATA ANALYSIS

ITEM	GRADE 1 TOXICITY	GRADE 2 TOXICITY	GRADE 3 TOXICITY	GRADE 4 TOXICITY
**HEMATOLOGY**				
Haemoglobin	8.0-9.4 gm/dL	7.0-7.9 gm/dL	6.5-6.9 gm/dL	<6.5 gm/dL
Absolute Neutrophil Count	1000-1500/mm^3	750-999/mm^3	500-749/mm^3	<500/mm^3
Absolute WBC Count	2001-3000/mm^3	1501-2000/mm^3	1001-1500/mm^3	<1000/mm^3
Platelets	75,000-99,000/mm^3	50,000-74.999/mm^3	20,000-49,999/mm^3	<20,000/mm^3 or diffuse petechiae
Prothrombin Time (PT)	1.0-1.25 X upper normal limit	>1.25-1.5 X upper normal limit	>1.5-3.0 X upper normal limit	>3 X upper normal limit
Activated Partial Thromboplastin Time (APTT)	>1.0-1.66 X upper normal limit	>1.66-2.33 X upper normal limit	>2.33-3 X upper normal limit	>3 X upper normal limit
Fibrinogen	0.75-0.99 X lower normal limit	0.50-0.74 X lower normal limit	0.25-0.49 X lower normal limit	<0.25 X lower normal limit
Fibrin Split Product	20-40 µg/ml	41-50 µg/ml	51-60 µg/ml	>60 µg/ml
Methemoglobin	5-10.0%	10.1-15%	15.1-20%	>20%
**ENZYMES**				
AST (SGOT)	1.25-2.5 X upper normal limit	>2.5-5 X upper normal limit	>5.0-10 X upper normal limit	>10 X upper normal limit
ALT (SGPT)	1.25-2.5 X upper normal limit	>2.5-5 X upper normal limit	>5.0-10 X upper normal limit	>10 X upper normal limit
GGT	1.25-2.5 X upper normal limit	>2.5-5 X upper normal limit	>5.0-10 X upper normal limit	>10 X upper normal limit
Alkaline Phosphatase	1.25-2.5 X upper normal limit	>2.5-5 X upper normal limit	>5.0-10 X upper normal limit	>10 X upper normal limit
Amylase	1.1-1.5 X upper normal limit	>1.5-2.0 X upper normal limit	>2.0-5.0 X upper normal limit	>5.1 X upper normal limit
Creatine phosphokinase (CPK)	>1.0-2.0 X upper normal limit	>2.0-4.0 X upper normal limit	>4.0-6.0 X upper normal limit	>6.1 X upper normal limit
**CHEMISTRIES**				
Hyponatremia	130-135 meq/L	123-129 meq/L	116-122 meq/L	<116 meq/L OR mental status changes OR seizures
Hypernatremia	146-150 meq/L	151-157 meq/L	158-165 meq/L	>165 meq/L OR mental status changes OR seizures
Hypokalemia	3.0-3.4 meq/L	2.5-2.9 meq/L OR replacement Rx req	2.0-2.4 meq/L OR intensive replacement Rx req. OR hospitalisation req.	<2.0 meq/L OR paresis OR ileus OR life-threatening arrhythmia
Hyperkalemia	5.6-6.0 meq/L	6.1-6.5 meq/L	6.6-7.0 meq/L	>7.0 meq/L OR life-threatening arrhythmia

APPENDIX D1: LABORATORY TEST ABNORMALITIES (CONTINUED)

ITEM	GRADE 1 TOXICITY	GRADE 2 TOXICITY	GRADE 3 TOXICITY	GRADE 4 TOXICITY
**CHEMISTRIES (Cont.)**				
Hypoglycemia	55-64 mg/dL	40-54 mg/dL	30-39 mg/dL	<30 mg/dL OR mental status changes OR coma
Hyperglycemia: (nonfasting and no prior diabetes)	116-160 mg/dL	161-250 mg/dL	251-500 mg/dL	>500 mg/dL OR ketoacidosis OR seizures
Hypocalcemia - correct for albumin	7.8-8.4 mg/dL	7.0-7.7 mg/dL	6.1-6.9 mg/dL	<6.1 mg/dL OR life-threatening arrhythmia OR tetany
Hypercalcemia - correct for albumin	10.6-11.5 mg/dL	11.6-12.5 mg/dL	12.6-13.5 mg/dL	>13.5 mg/dL OR life-threatening arrhythmia
Hypomagnesemia	1.2-1.4 meq/dL	0.9-1.1 meq/L OR replacement Rx req.	0.6-0.8 meq/L OR intensive Rx req. OR hospitalisation	<0.6 meq/L OR life-threatening arrhythmia
Hypophosphatemia	2.0-2.4 mg/dL	1.5-1.9 mg/dL OR replacement Rx req.	1.0-1.4 mg/dL OR intensive Rx req. OR hospitalisation req.	<1.0 mg/dL OR life-threatening arrhythmia
Hyperbilirubinemia	>1.0-1.5 X upper normal limit	>1.5-2.5 X upper normal limit	>2.5-5 X upper normal limit	>5 X upper normal limit
Blood Urea Nitrogen (BUN)	>1.25-2.5 X upper normal limit	>2.5-5 X upper normal limit	>5.0-10 X upper normal limit	>10 X upper normal limit
Creatinine	>1.0-1.5 X upper normal limit	>1.5-3.0 X upper normal limit	>3.0-6 X upper normal limit	>6 X upper normal limit OR dialysis required
Serum Lactate	>1.0-2.0 X upper normal limit	>2.0-3.0 X upper normal limit	>3.0-3.9 X upper normal limit	>4.0 X upper normal limit
Albumin	>1.0-1.5X upper normal limit	>1.5-2.0 X upper normal limit	>2.0-5.0 X upper normal limit	>5.0 X upper normal limit
Triglycerides	ULN -399 mg/dL (ULN - 4.50 mmol/L)	400 - 750 mg/dL (4.51 - 8.50 mmol/L)	751 - 1200 mg/dL (8.51 - 13.5 mmol/L)	>1200 mg/dL >13.50 mmol/L
Cholesterol	>1.0-1.3 X upper normal limit	>1.3-1.6 X upper normal limit	>1.6-2.0 X upper normal limit	>2.0 X upper normal limit
**URINALYSIS**				
Proteinuria	1+ OR <0.3% OR <3 g/L OR 200 mg-1 gm loss/day	2-3+ OR 0.3-1.0% OR 3-10 g/L OR 1-2 gm loss/day	4+ OR >1.0% OR >10 g/L OR 2-3.5 gm loss/day	>3.5 gm loss/day OR nephrotic syndrome
Hematuria	microscopic only	gross, no clots	gross+clots	requires transfusion OR obstructive

APPENDIX D2: CLINICAL ADVERSE EVENTS

Table for Grading Severity of Adverse Events for Dose Modification Purposes

ITEM	GRADE 1 TOXICITY	GRADE 2 TOXICITY	GRADE 3 TOXICITY	GRADE 4 TOXICITY
**CARDIAC DYSFUNCTION**				
Cardiac Arrhythmia	- -	asymptomatic, transient dysrhythmia, no Rx required	recurrent/persistent; dysrhythmia; symptomatic Rx required	unstable dysrhythmia, hospitalisation and Rx required
Hypertension	transient inc. >20 mm/Hg; no Rx	recurrent, chronic, >20 mm/Hg, Rx req.	requires acute therapy; outpatient hospitalisation possible	requires hospitalisation
Hypotension	transient orthostatic hypotension; No Rx	symptoms correctable with oral fluid Rx	requires IV fluids no. hosp. required	requires hospitalisation
Pericarditis	minimal effusion	mild/mod asymp. effusion, no Rx	symptomatic effusion, pain, EKG changes	tamponade; OR pericardiocentesis OR surgery required
Haemorrhage, Blood Loss	- -	mildly symptomatic or Rx required	gross blood loss; OR 1-2 units transfused	massive blood loss OR >2 units transfused
**RESPIRATORY**				
Cough - for aerosol studies	transient - no Rx	treatment associated cough; inhaled bronchodilator	uncontrolled cough systemic Rx required	- -
Bronchospasm acute	transient; no Rx; <80%->70% FEV_1 (or peak flow)	req. Rx; normalises with bronchodilator FEV_1 50%-70% (or peak flow)	no normalisation w/ bronchodilator FEV_1 25%-50% (or peak flow), retractions	cyanosis FEV_1 <25% (or peak flow) OR intubated
**NEUROLOGIC**				
Neuro-Cerebellar	slight incoordination OR dysdiadokinesia	intention tremor, OR dysmetria, or slurred speech; OR nystagmus	Ataxia requiring assistance to walk or arm incoordination interfering with ADLS	unable to stand
Neuro-psych/mood	- -	- -	severe mood changes requiring medical intervention	acute psychosis require hospitalisation

APPENDIX D2: CLINICAL ADVERSE EVENTS

ITEM	GRADE 1 TOXICITY	GRADE 2 TOXICITY	GRADE 3 TOXICITY	GRADE 4 TOXICITY
**NEUROLOGIC**	**(continued)**			
Paresthesia (burning, tingling, etc)	mild discomfort; no Rx req	mod discomfort; non-narcotic analgesia req	severe discomfort; OR narcotic analgesia req with symptomatic improvement	incapacitating; OR not responsive to narcotic analgesia
Neuro-motor	mild weakness in muscle of feet but able to walk and/or mild increase or decrease in reflexes	Mod weakness in feet (unable to walk on heels and/or toes), mild weakness in hands, still able to do most hand tasks and/or loss of previously present reflex or development of hyperreflexia and/or unable to do deep knee bends due to weakness	Marked distal weakness (unable to dorsiflex toes or foot drop), and mod proximal weakness e.g., in hands interfering with ADLs and/or requiring assistance to walk and/or unable to rise from chair unassisted	confined to bed or wheel chair because of muscle weakness
Neuro-sensory	Mild impairment (dec sensation, e.g., vibratory, pinprick, hot/cold in great toes) in focal area or symmetrical distribution	Mod impairment (mod dec sensation, e.g., vibratory, pinprick, hot/cold to ankles) and/or joint position or mild impairment that is not symmetrical	severe impairment (dec or loss of sensation to knees or wrists) or loss of sensation of at least mod degree in multiple different body areas (i.e., upper and lower extremities)	sensory loss involves limbs and trunk.
**GASTROINTESTINAL**				
Nausea	mild discomfort; maintains reasonable intake	mod. discomfort; intake dec. for < 3 days	severe discomfort; minimal intake for 3 days	hospitalisation required
Constipation	mild	moderate	severe	distension with vomiting
Abdominal pain	mild discomfort; no limits on activity	mild-moderate discomfort; no Rx required	moderate pain; Rx required	severe pain; hospitalisation required
Vomiting	mild or transient; 2-3 episodes per day OR mild vomiting lasting < 1 week	mod or persistent; 4-5 episodes per day OR vomiting lasting 1 week	severe vomiting of all food/fluids in 24 hrs OR orthostatic hypotension OR IV Rx req.	hypotensive shock OR hospitalisation req. for IV Rx req.
Diarrhoea	mild or transient; 3-4 loose stools per day OR mild diarrhoea lasting < 1 week	mod OR persistent; 5-7 loose stools per day OR diarrhoea lasting 1 week	bloody diarrhoea, OR orthostatic hypotension OR > 7 loose stools/day OR IV Rx req.	hypotensive shock OR hospitalisation req.
Oral dyscomfort/ dysphagia	mild discomfort, no difficulty swallowing	difficulty swallowing but able to eat and drink	unable to swallow solids	unable to drink fluids; IV fluids req.

APPENDIX D2: CLINICAL ADVERSE EVENTS

ITEM	GRADE 1 TOXICITY	GRADE 2 TOXICITY	GRADE 3 TOXICITY	GRADE 4 TOXICITY
**OTHER PARAMETERS**				
Fever oral, >12 hours	37.7-38.5C OR 100.0-101.5F	38.6-39.5C OR 101.6-102 F	39.6-40.5C OR 103-105F	>40.5C OR >105F
Headache	mild, no Rx therapy	mod; OR non-narcotic analgesia Rx	severe OR responds to initial narcotic therapy	intractable, OR req. repeated narcotic therapy
Fatigue	normal activity reduced <25%	normal activity dec. 25-50%	normal activity dec. >50%; can't work	unable to care for self
Allergic Reaction	pruritus w/o rash	localised urticaria	generalised urticaria angioedema	anaphylaxis
Cutaneous/Rash/ Dermatitis	erythema, pruritus	diffuse, maculopapular rash, OR dry desquamation	vesiculation, OR moist desquamation, OR ulceration	exfoliative dermatitis, OR mucous membrane involvement, OR erythema, multiforme OR suspected Stevens-Johnson OR necrosis requiring surgery
Local Reaction (2° parenteral Rx not vaccination or skin test.	Erythema	induration <10 mm OR inflammation OR phlebitis	induration >10 mm OR ulceration	necrosis of skin
Clinical symptoms not otherwise specified in this table	No therapy; monitor condition	May require minimal intervention and monitoring	Requires medical care and possible hospitalisation	Requires active medical intervention, hospitalisation, or hospice care
Laboratory values not otherwise specified in this table	Abnormal, but requiring no immediate intervention; follow	Sufficiently abnormal to require evaluation as to causality and perhaps mild therapeutic intervention, but not of sufficient severity to warrant immediate changes in study drug	Sufficiently severe to require evaluation and treatment, including at least temporary suspension of study drug	Life-threatening severity. Requires immediate evaluation, treatment, and usually hospitalisation. Study drug must be stopped immediately and should not be restarted until the abnormality is clearly felt to be caused by some other mechanism than study drug

Appendix D: Grading of Adverse Events

APPENDIX D1: LABORATORY TEST ABNORMALITIESTABLE FOR GRADING SEVERITY OF ADVERSE EVENTS FOR DOSE MODIFICATION PURPOSES AND DATA ANALYSIS

ITEM	GRADE 1 TOXICITY	GRADE 2 TOXICITY	GRADE 3 TOXICITY	GRADE 4 TOXICITY
**HEMATOLOGY**				
Haemoglobin	8.0-9.4 gm/dL	7.0-7.9 gm/dL	6.5-6.9 gm/dL	<6.5 gm/dL
Absolute Neutrophil Count	1000-1500/mm^3	750-999/mm^3	500-749/mm^3	<500/mm^3
Absolute WBC Count	2001-3000/mm^3	1501-2000/mm^3	1001-1500/mm^3	<1000/mm^3
Platelets	75,000-99,000/mm^3	50,000-74.999/mm^3	20,000-49,999/mm^3	<20,000/mm^3 or diffuse petechiae
Prothrombin Time (PT)	1.0-1.25 X upper normal limit	>1.25-1.5 X upper normal limit	>1.5-3.0 X upper normal limit	>3 X upper normal limit
Activated Partial Thromboplastin Time (APTT)	>1.0-1.66 X upper normal limit	>1.66-2.33 X upper normal limit	>2.33-3 X upper normal limit	>3 X upper normal limit
Fibrinogen	0.75-0.99 X lower normal limit	0.50-0.74 X lower normal limit	0.25-0.49 X lower normal limit	<0.25 X lower normal limit
Fibrin Split Product	20-40 µg/ml	41-50 µg/ml	51-60 µg/ml	>60 µg/ml
Methemoglobin	5-10.0%	10.1-15%	15.1-20%	>20%
**ENZYMES**				
AST (SGOT)	1.25-2.5 X upper normal limit	>2.5-5 X upper normal limit	>5.0-10 X upper normal limit	>10 X upper normal limit
ALT (SGPT)	1.25-2.5 X upper normal limit	>2.5-5 X upper normal limit	>5.0-10 X upper normal limit	>10 X upper normal limit
GGT	1.25-2.5 X upper normal limit	>2.5-5 X upper normal limit	>5.0-10 X upper normal limit	>10 X upper normal limit
Alkaline Phosphatase	1.25-2.5 X upper normal limit	>2.5-5 X upper normal limit	>5.0-10 X upper normal limit	>10 X upper normal limit
Amylase	1.1-1.5 X upper normal limit	>1.5-2.0 X upper normal limit	>2.0-5.0 X upper normal limit	>5.1 X upper normal limit
Creatine phosphokinase (CPK)	>1.0-2.0 X upper normal limit	>2.0-4.0 X upper normal limit	>4.0-6.0 X upper normal limit	>6.1 X upper normal limit
**CHEMISTRIES**				
Hyponatremia	130-135 meq/L	123-129 meq/L	116-122 meq/L	<116 meq/L OR mental status changes OR seizures
Hypernatremia	146-150 meq/L	151-157 meq/L	158-165 meq/L	>165 meq/L OR mental status changes OR seizures
Hypokalemia	3.0-3.4 meq/L	2.5-2.9 meq/L OR replacement Rx req	2.0-2.4 meq/L OR intensive replacement Rx req. OR hospitalisation req.	<2.0 meq/L OR paresis OR ileus OR life-threatening arrhythmia
Hyperkalemia	5.6-6.0 meq/L	6.1-6.5 meq/L	6.6-7.0 meq/L	>7.0 meq/L OR life-threatening arrhythmia

APPENDIX D1: LABORATORY TEST ABNORMALITIES (CONTINUED)

ITEM	GRADE 1 TOXICITY	GRADE 2 TOXICITY	GRADE 3 TOXICITY	GRADE 4 TOXICITY
**CHEMISTRIES (Cont.)**				
Hypoglycemia	55-64 mg/dL	40-54 mg/dL	30-39 mg/dL	<30 mg/dL OR mental status changes OR coma
Hyperglycemia: (nonfasting and no prior diabetes)	116-160 mg/dL	161-250 mg/dL	251-500 mg/dL	>500 mg/dL OR ketoacidosis OR seizures
Hypocalcemia - correct for albumin	7.8-8.4 mg/dL	7.0-7.7 mg/dL	6.1-6.9 mg/dL	<6.1 mg/dL OR life-threatening arrhythmia OR tetany
Hypercalcemia - correct for albumin	10.6-11.5 mg/dL	11.6-12.5 mg/dL	12.6-13.5 mg/dL	>13.5 mg/dL OR life-threatening arrhythmia
Hypomagnesemia	1.2-1.4 meq/dL	0.9-1.1 meq/L OR replacement Rx req.	0.6-0.8 meq/L OR intensive Rx req. OR hospitalisation	<0.6 meq/L OR life-threatening arrhythmia
Hypophosphatemia	2.0-2.4 mg/dL	1.5-1.9 mg/dL OR replacement Rx req.	1.0-1.4 mg/dL OR intensive Rx req. OR hospitalisation req.	<1.0 mg/dL OR life-threatening arrhythmia
Hyperbilirubinemia	>1.0-1.5 X upper normal limit	>1.5-2.5 X upper normal limit	>2.5-5 X upper normal limit	>5 X upper normal limit
Blood Urea Nitrogen (BUN)	>1.25-2.5 X upper normal limit	>2.5-5 X upper normal limit	>5.0-10 X upper normal limit	>10 X upper normal limit
Creatinine	>1.0-1.5 X upper normal limit	>1.5-3.0 X upper normal limit	>3.0-6 X upper normal limit	>6 X upper normal limit OR dialysis required
Serum Lactate	>1.0-2.0 X upper normal limit	>2.0-3.0 X upper normal limit	>3.0-3.9 X upper normal limit	>4.0 X upper normal limit
Albumin	>1.0-1.5X upper normal limit	>1.5-2.0 X upper normal limit	>2.0-5.0 X upper normal limit	>5.0 X upper normal limit
Triglycerides	ULN -399 mg/dL (ULN - 4.50 mmol/L)	400 - 750 mg/dL (4.51 - 8.50 mmol/L)	751 - 1200 mg/dL (8.51 - 13.5 mmol/L)	>1200 mg/dL >13.50 mmol/L
Cholesterol	>1.0-1.3 X upper normal limit	>1.3-1.6 X upper normal limit	>1.6-2.0 X upper normal limit	>2.0 X upper normal limit
**URINALYSIS**				
Proteinuria	1+ OR <0.3% OR <3 g/L OR 200 mg-1 gm loss/day	2-3+ OR 0.3-1.0% OR 3-10 g/L OR 1-2 gm loss/day	4+ OR >1.0% OR >10 g/L OR 2-3.5 gm loss/day	>3.5 gm loss/day OR nephrotic syndrome
Hematuria	microscopic only	gross, no clots	gross+clots	requires transfusion OR obstructive

APPENDIX D2: CLINICAL ADVERSE EVENTS

Table for Grading Severity of Adverse Events for Dose Modification Purposes

ITEM	GRADE 1 TOXICITY	GRADE 2 TOXICITY	GRADE 3 TOXICITY	GRADE 4 TOXICITY
**CARDIAC DYSFUNCTION**				
Cardiac Arrhythmia	- -	asymptomatic, transient dysrhythmia, no Rx required	recurrent/persistent; dysrhythmia; symptomatic Rx required	unstable dysrhythmia, hospitalisation and Rx required
Hypertension	transient inc. >20 mm/Hg; no Rx	recurrent, chronic, >20 mm/Hg, Rx req.	requires acute therapy; outpatient hospitalisation possible	requires hospitalisation
Hypotension	transient orthostatic hypotension; No Rx	symptoms correctable with oral fluid Rx	requires IV fluids no. hosp. required	requires hospitalisation
Pericarditis	minimal effusion	mild/mod asymp. effusion, no Rx	symptomatic effusion, pain, EKG changes	tamponade; OR pericardiocentesis OR surgery required
Haemorrhage, Blood Loss	- -	mildly symptomatic or Rx required	gross blood loss; OR 1-2 units transfused	massive blood loss OR >2 units transfused
**RESPIRATORY**				
Cough - for aerosol studies	transient - no Rx	treatment associated cough; inhaled bronchodilator	uncontrolled cough systemic Rx required	- -
Bronchospasm acute	transient; no Rx; <80%->70% FEV_1 (or peak flow)	req. Rx; normalises with bronchodilator FEV_1 50%-70% (or peak flow)	no normalisation w/ bronchodilator FEV_1 25%-50% (or peak flow), retractions	cyanosis FEV_1 <25% (or peak flow) OR intubated
**NEUROLOGIC**				
Neuro-Cerebellar	slight incoordination OR dysdiadokinesia	intention tremor, OR dysmetria, or slurred speech; OR nystagmus	Ataxia requiring assistance to walk or arm incoordination interfering with ADLS	unable to stand
Neuro-psych/mood	- -	- -	severe mood changes requiring medical intervention	acute psychosis require hospitalisation

APPENDIX D2: CLINICAL ADVERSE EVENTS

ITEM	GRADE 1 TOXICITY	GRADE 2 TOXICITY	GRADE 3 TOXICITY	GRADE 4 TOXICITY
**NEUROLOGIC**	**(continued)**			
Paresthesia (burning, tingling, etc)	mild discomfort; no Rx req	mod discomfort; non-narcotic analgesia req	severe discomfort; OR narcotic analgesia req with symptomatic improvement	incapacitating; OR not responsive to narcotic analgesia
Neuro-motor	mild weakness in muscle of feet but able to walk and/or mild increase or decrease in reflexes	Mod weakness in feet (unable to walk on heels and/or toes), mild weakness in hands, still able to do most hand tasks and/or loss of previously present reflex or development of hyperreflexia and/or unable to do deep knee bends due to weakness	Marked distal weakness (unable to dorsiflex toes or foot drop), and mod proximal weakness e.g., in hands interfering with ADLs and/or requiring assistance to walk and/or unable to rise from chair unassisted	confined to bed or wheel chair because of muscle weakness
Neuro-sensory	Mild impairment (dec sensation, e.g., vibratory, pinprick, hot/cold in great toes) in focal area or symmetrical distribution	Mod impairment (mod dec sensation, e.g., vibratory, pinprick, hot/cold to ankles) and/or joint position or mild impairment that is not symmetrical	severe impairment (dec or loss of sensation to knees or wrists) or loss of sensation of at least mod degree in multiple different body areas (i.e., upper and lower extremities)	sensory loss involves limbs and trunk.
**GASTROINTESTINAL**				
Nausea	mild discomfort; maintains reasonable intake	mod. discomfort; intake dec. for < 3 days	severe discomfort; minimal intake for 3 days	hospitalisation required
Constipation	mild	moderate	severe	distension with vomiting
Abdominal pain	mild discomfort; no limits on activity	mild-moderate discomfort; no Rx required	moderate pain; Rx required	severe pain; hospitalisation required
Vomiting	mild or transient; 2-3 episodes per day OR mild vomiting lasting < 1 week	mod or persistent; 4-5 episodes per day OR vomiting lasting 1 week	severe vomiting of all food/fluids in 24 hrs OR orthostatic hypotension OR IV Rx req.	hypotensive shock OR hospitalisation req. for IV Rx req.
Diarrhoea	mild or transient; 3-4 loose stools per day OR mild diarrhoea lasting < 1 week	mod OR persistent; 5-7 loose stools per day OR diarrhoea lasting 1 week	bloody diarrhoea, OR orthostatic hypotension OR > 7 loose stools/day OR IV Rx req.	hypotensive shock OR hospitalisation req.
Oral dyscomfort/ dysphagia	mild discomfort, no difficulty swallowing	difficulty swallowing but able to eat and drink	unable to swallow solids	unable to drink fluids; IV fluids req.

APPENDIX D2: CLINICAL ADVERSE EVENTS

ITEM	GRADE 1 TOXICITY	GRADE 2 TOXICITY	GRADE 3 TOXICITY	GRADE 4 TOXICITY
**OTHER PARAMETERS**				
Fever oral, >12 hours	37.7-38.5C OR 100.0-101.5F	38.6-39.5C OR 101.6-102 F	39.6-40.5C OR 103-105F	>40.5C OR >105F
Headache	mild, no Rx therapy	mod; OR non-narcotic analgesia Rx	severe OR responds to initial narcotic therapy	intractable, OR req. repeated narcotic therapy
Fatigue	normal activity reduced <25%	normal activity dec. 25-50%	normal activity dec. >50%; can't work	unable to care for self
Allergic Reaction	pruritus w/o rash	localised urticaria	generalised urticaria angioedema	anaphylaxis
Cutaneous/Rash/ Dermatitis	erythema, pruritus	diffuse, maculopapular rash, OR dry desquamation	vesiculation, OR moist desquamation, OR ulceration	exfoliative dermatitis, OR mucous membrane involvement, OR erythema, multiforme OR suspected Stevens-Johnson OR necrosis requiring surgery
Local Reaction (2° parenteral Rx not vaccination or skin test.	Erythema	induration <10 mm OR inflammation OR phlebitis	induration >10 mm OR ulceration	necrosis of skin
Clinical symptoms not otherwise specified in this table	No therapy; monitor condition	May require minimal intervention and monitoring	Requires medical care and possible hospitalisation	Requires active medical intervention, hospitalisation, or hospice care
Laboratory values not otherwise specified in this table	Abnormal, but requiring no immediate intervention; follow	Sufficiently abnormal to require evaluation as to causality and perhaps mild therapeutic intervention, but not of sufficient severity to warrant immediate changes in study drug	Sufficiently severe to require evaluation and treatment, including at least temporary suspension of study drug	Life-threatening severity. Requires immediate evaluation, treatment, and usually hospitalisation. Study drug must be stopped immediately and should not be restarted until the abnormality is clearly felt to be caused by some other mechanism than study drug

APPENDIX XI

SERIOUS ADVERSE EVENTS DECLARATION FORM

STUDY MANON 05-CUTAVAC **Page 1/3**

|__|__|__| |__|__||__|__| **Investigator's name** **Baseline**

Subjects's Initials **Subject N°** --------------------------------

NOTIFICATION OF SERIOUS ADVERSE EVENT TO THE SPONSOR
TO be transmitted to the ORVACS monitor by FAX within 24 H
Fax n°: 33 (0) 1 42 16 42 61/67 or 33 (0) 1 44 24 17 96

INITIAL REPORT **FOLLOW-UP REPORT N°** |__||__|

TRANSMISSION DATE: |___|___| |___|___| |___|___||___|___| COUNTRY OF ORIGIN -------------------------

SUBJECT DEMOGRAPHICS

Birth date: |__|__||__|__| |__|__|__|__| Height: |___|___|__| cm Weight: |___|___|__| **.** |___| Kg
Gender: **M** ☐ F ☐

1. DESCRIPTION OF THE EVENT

Date of occurrence |___|___| d |___|___| m |____|____|____|___|y

The serious event:

- ☐ Results in death (whatever might be the cause)
- ☐ Requires hospitalization (*) or extension thereof
- ☐ Is life threatening
- ☐ Results in persistent or significant disability or incapacity

Other facts to be notified

- ☐ Pregnancy or congenital abnormally
- ☐ Overdosage
- ☐ Cancer
- ☐ Others (any other medical or biological relevant event according to the investigator or the protocol)

Nature of the event (if clinical nature, indicate the diagnosis or the major symptom):

Summary description (if clinical cause : significant medical history immediate outcome, available biological results, etc…):

Blood sample (intended for diagnosis) available? *Yes* ☐ *No* ☐

(*) Except hospitalization which duration and principle are planned by the protocol

Investigator's name: **Signature:**

STUDY MANON 05-CUTAVAC **Page 2/3**

**|__|__|__|**	**|__|__||__|__|**	**Investigator's name**	**Baseline**
**Subjects's Initials**	**Subject N°**	**--------------------------------**	

INITIAL REPORT FOLLOW-UP REPORT N° |__||__|

2. INFORMATION ON STUDY PRODUCT

Name	Dose	Total dose	Treatment Schedule	Allocated Arm	Date of administration (dd/mm/yy)
**Tetagrip®vaccine**	0.5 ml	0.5 ml	Once at D0		____/ ____/____

Site of injection: .... Route of administration: ....

Left ☐ Right ☐

3. CONCOMITANT MEDICATION FROM TRIAL INITIATION TO THE OCURRENCE OF THE SAE

INN or trade name	Primary booster (1) or daily dose	Start date (dd/mm/yy)	Ongoing at ocurrence of the event	Stop date (dd/mm/yy)	Route of administration (2) or Site of injection (3)	Indication
		____/ ____/____	☐	____/ ____/____		
		____/ ____/____	☐	____/ ____/____		
		____/ ____/____	☐	____/ ____/____		
		____/ ____/____	☐	____/ ____/____		

Comments: ....
....

(1) For Primary immunization, indicate P1,P2,P3, for Booster indicate B1, B2, B3, if not a vaccine NA (2) IV / IM / SC / ID / PO
(3) Arm / Back / Thigh / Buttock or NA

4. MEASURES TAKEN FOLLOWING THE EVENT

- **Regarding the study treatment**

1 No modification

2 Dosage modification, specify: ………………………………… Date of modification ____/ ____/____

☐ 3 Course delay, specify : ………………………………………… Date of administration ____/ ____/____

☐ 4 Permanent discontinuation Date of discontinuation ____/ ____/____

The event leads to:

- **The prescription of corrective or symptomatic treatments**, specify (names, dosages)*:

Product INN or trade name	daily dose	Start date (dd/mm/yy)	Stop date (dd/mm/yy)	Route of administration	Indication
		____/ ____/____	____/ ____/____		
		____/ ____/____	____/ ____/____		
		____/ ____/____	____/ ____/____		

Investigator's name: **Signature:**

STUDY MANON 05-CUTAVAC **Page 3/3**

|__|__|__| |__|__||__|__| **Investigator's name**
Baseline

Subjects's Initials **Subject N°** --------------------------------

INITIAL REPORT **FOLLOW-UP REPORT N°** |__||__|

5. DESCRIPTION OF LOCAL AND SYSTEMIC SYMPTOMS RELATED TO THE SERIOUS ADVERSE EVENT

Symptoms	Onset date (dd/mm/yy)	Stop date (dd/mm/yy)	Duration	Severity
	___/ ___/___	___/ ___/___		
	___/ ___/___	___/ ___/___		
	___/ ___/___	___/ ___/___		
	___/ ___/___	___/ ___/___		
	___/ ___/___	___/ ___/___		

1 : mild - 2 : moderate - 3 : severe

6. DESCRIPTION OF RELEVANT ON-GOING ILLNESS / MEDICAL HISTORY / RISK FACTORS

....
....
....
....

7. OUTCOME

☐ Recovery ☐ Worsening ☐ Improvement ☐ Sequelae ☐ Stabilization
☐ Ongoing ☐ Unknown ☐ Death Date of death |__|__|dd |__|__|mm |__|__|yy
In case of death, has an autopsy been conducted? Yes ☐ NO ☐

8. INVESTIGATOR OPINION AS REGARDS THE RELATIONSHIP BETWEEN THE EVENT AND THE STUDY TREATMENT

☐ Very likely ☐ Likely ☐ Possible ☐ Dubious ☐ No relationship ☐ Insufficient data

COMMENTS:

*** Enclose in this notification the results of examinations carried out and the hospitalization reports, etc.**

Investigator's name: **Signature:**
