# Supplementary material for: Preferential Amplification of CD8 Effector-T Cells after Transcutaneous Application of an Inactivated Influenza Vaccine: A Randomized Phase I Trial
Source: PLoS One. 2010 May 26;5(5):e10818. doi: 10.1371/journal.pone.0010818 (PMC2877091; doi:10.1371/journal.pone.0010818)

The Consort E-Flowchart Aug. 2005

Cohorte I: Healthy Volunteers

Assessed for eligibility
Healthy volunteers (n=24)

Enrollment

Excluded (n=   )
Not meeting inclusion criteria
(n=   )
Refused to participate
(n=0)
Other reasons
(n=   )

Randomized (n=24)

Allocation

Allocated to intervention (TC)
Healthy volunteers (n=12)
Received allocated intervention
Healthy volunteers (n=12)
Did not receive allocated intervention
(n=0/ 0)
Give reasons

Allocated to intervention (IM)
Healthy volunteers (n=12)
Received allocated intervention
Healthy volunteers (n=12)
Did not receive allocated intervention
(n=0/ 0)
Give reasons

Follow-Up

Lost to follow-up (n=0)
Give reasons

Discontinued intervention
(n=   )
Give reasons

Lost to follow-up (n= 0)
Give reasons

Discontinued intervention
(n=   )
Give reasons

Analysis

Analyzed
Healthy volunteers (n=12)
Excluded from analysis (n=0/ 0)
Give reasons

Analyzed
Healthy volunteers (n= 12)
Excluded from analysis (n= 0/ 0)
Give reasons

The Consort E-Flowchart Aug. 2005

Cohorte II: HIV-Infected Patients

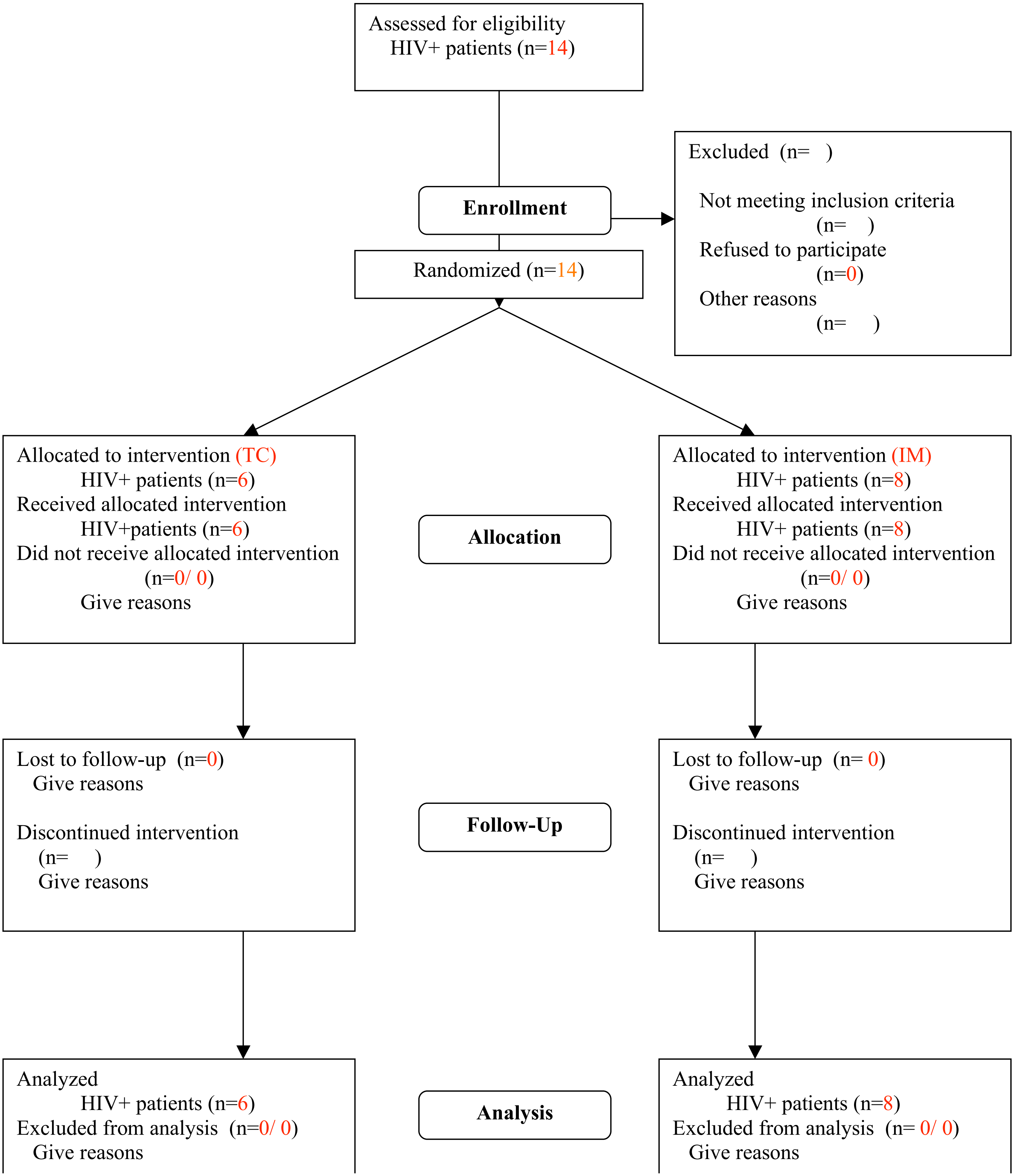

Supplement: Flowchart S1 — Flowchart cohort I and cohort II. (0.11 MB PDF) [file pone.0010818.s005.pdf]
